# Immune landscape and a promising immune prognostic model associated with TP53 in early‐stage lung adenocarcinoma

**DOI:** 10.1002/cam4.3655

**Published:** 2020-12-12

**Authors:** Chengde Wu, Xiang Rao, Wei Lin

**Affiliations:** ^1^ Department of Thoracic Surgery Affiliated Haikou Hospital of Xiangya Medical College Central South University Haikou Hainan China; ^2^ Department of Pathology Affiliated Haikou Hospital of Xiangya Medical College Central South University Haikou Hainan China

**Keywords:** CIBERSORT, early‐stage lung adenocarcinoma, immune prognostic model, prognosis, TP53

## Abstract

**Purpose:**

TP53 mutation, one of the most frequent mutations in early‐stage lung adenocarcinoma (LUAD), triggers a series of alterations in the immune landscape, progression, and clinical outcome of early‐stage LUAD. Our study was designed to unravel the effects of TP53 mutation on the immunophenotype of early‐stage LUAD and formulate a TP53‐associated immune prognostic model (IPM) that can estimate prognosis in early‐stage LUAD patients.

**Materials and methods:**

Immune‐associated differentially expressed genes (DEGs) between TP53 mutated (TP53^MUT^) and TP53 wild‐type (TP53^WT^) early‐stage LUAD were comprehensively analyzed. Univariate Cox analysis and least absolute shrinkage and selection operator (LASSO) analysis identified the prognostic immune‐associated DEGs. We constructed and validated an IPM based on the TCGA and a meta‐GEO composed of GSE72094, GSE42127, and GSE31210, respectively. The CIBERSORT algorithm was analyzed for assessing the percentage of immune cell types. A nomogram model was established for clinical application.

**Results:**

TP53 mutation occurred in approximately 50.00% of LUAD patients, stimulating a weakened immune response in early‐stage LUAD. Sixty‐seven immune‐associated DEGs were determined between TP53^WT^ and TP53^MUT^ cohort. An IPM consisting of two prognostic immune‐associated DEGs (risk score = 0.098 * ENTPD2 expression + 0.168 * MIF expression) was developed through 397 cases in the TCGA and further validated based on 623 patients in a meta‐GEO. The IPM stratified patients into low or high risk of undesirable survival and was identified as an independent prognostic indicator in multivariate analysis (HR = 2.09, 95% CI: 1.43–3.06, *p* < 0.001). Increased expressions of PD‐L1, CTLA‐4, and TIGIT were revealed in the high‐risk group. Prognostic nomogram incorporating the IPM and other clinicopathological parameters (TNM stage and age) achieved optimal predictive accuracy and clinical utility.

**Conclusion:**

The IPM based on TP53 status is a reliable and robust immune signature to identify early‐stage LUAD patients with high risk of unfavorable survival.

## Graphical abstract

An immune prognostic model based on TP53 status is a hopeful biomarker to optimize the prognosis of early‐stage lung adenocarcinoma.

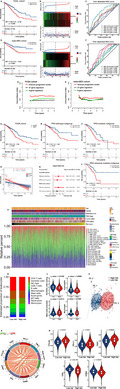

## 1 INTRODUCTION

Lung cancer is the leading reason for tumor‐associated death, and it is histologically categorized into two major subtypes: small‐cell lung carcinoma (SCLC) and non‐small‐cell lung carcinoma (NSCLC), accounting for approximately 15% and 85% of all cases, respectively.^1^, ^2^ Lung adenocarcinoma (LUAD), the most frequent subtype of NSCLC, comprises over 40% of all patients, which is derived from mucus‐secreting type II alveolar cells in small airway epithelium.^3^, ^4^ High rate of metastasis and invasiveness, one of the most striking characteristics of LUAD, yields a 5‐year survival rate of merely 19%.^5^ Moreover, a staggering proportion (57%) of LUAD cases initially diagnosed with metastatic neoplasm achieves a 5‐year survival rate of merely 5%.^6^ Conversely, low‐dose computerized tomography (CT) is conducive to the rapid screening of early‐stage LUAD.^7^, ^8^ Those with localized tumor exhibit a relatively desirable 5‐year survival rate of up to 83% (TNM stage IA) and 71% (TNM stage IB).^9^ Based on several large and randomized clinical trials, a majority of early‐stage LUAD cases are not recommended to receive adjuvant systemic chemotherapy following surgical intervention, which is partly ascribed to chemotherapy‐associated toxic effects far exceeding the potential survival advantages for these individuals.^10^, ^11^ Therefore, it is essential to identify the subset of early‐stage LUAD patients (TNM I and Ⅱ stage) with high possibility of recurrence and death, for whom additional systemic chemotherapy is required.^8^, ^12^

Immune escape has been considered as an emerging hallmark of lung cancer.^13^ Novel and promising immune checkpoint inhibitors' (ICIs) treatment targeted programmed death 1(PD‐1)/programmed death ligand 1 (PD‐L1) has been revealed a prominent and durable response in approximately 20% NSCLC patients.^14^ The ideal method of patient selection for the optimal improvement in the effectiveness of frontline immunotherapy for NSCLC remains to be determined.^15^ The levels of immunosuppressive molecules (such as PD‐L1, PD‐1, and indoleamine 2,3‐dioxygenase [IDO]), mutational landscape, and burden as well as mismatch repair deficiency have been identified as potential predictors of patient's response to ICIs therapy.^16^, ^17^ For example, a significantly increased progression‐free survival (PFS) was revealed in anti‐PD‐1 monotherapy (Nivolumab) combined with anti‐CTLA‐4 monotherapy (Ipilimumab) versus chemotherapy alone in NSCLC individuals with a high tumor mutational burden (TMB) rather than in those with a low TMB.^18^, ^19^ The subset of NSCLC cases with strongly PD‐L1‐positive neoplasms is the primary driver of clinical benefit from anti‐PD‐1 monotherapy (Pembrolizumab) in the whole research population.^20^ Additionally, certain immune‐associated clinicopathological parameters, such as enhanced levels of tumor‐infiltrating cytotoxic lymphocytes (CTLs), seem to be prognostic biomarkers and are significantly correlated with better prognosis in early‐stage LUAD, further highlighting the significance of multifarious components of the immune system during the initiation and progression of lung cancer.^21^ Thus, comprehensive exploration of the complex cross‐talk between tumor and immune microenvironment will be instrumental in evaluating their prognostic potential in early‐stage LUAD and optimizing tumor‐associated immunotherapy strategies.^22^

Tumor suppressor p53, a transcription factor, exerts its tumor suppressive function primarily via its transcriptional modulation of its downstream target genes.^23^ The products of p53 target genes are proved to participate in a sequence of crucial biological pathways, including cell proliferation and apoptosis, DNA damage repair, anti‐oxidant function, metabolism and angiogenesis as well as immunoreaction, thus making contributions to the tumor‐suppression effect of p53.^24^, ^25^, ^26^ TP53, the encoding gene of p53 protein, is the most commonly mutated gene in multiple cancers and is subsistent in approximately 37%‐50% LUAD cases.^27^, ^28^, ^29^ Mutant p53 potentially triggers chromosomal/genomic instability and further results in a high TMB, which is frequently related to more aggressive malignancy and unsatisfactory prognosis in LUAD.^30^, ^31^, ^32^ Nevertheless, several studies have highlighted that TP53‐mutated LUAD is characterized with higher PD‐L1 expression by malignant cells, boosted T‐cell infiltration and tumor immunogenicity, leading to an increased response to ICIs.^7^, ^32^, ^33^, ^34^

Therefore, TP53 mutation burden is a pivotal determinant and biomarker for targeted therapy response and prognosis of patients.

In our report, we combined TP53 mutation status information with mRNA expression profiles to investigate the association between TP53 mutation and immune landscape in early‐stage LUAD. An individualized IPM on the basis of immune‐related gene whose expression is influenced by TP53 mutation status was developed and validated in different populations and platforms, which can be served as a promising prognostic signature to improve early‐stage LUAD patient management.

## 2 MATERIALS AND METHODS

### 2.1 Data source and patient selection

**Figure 1:**
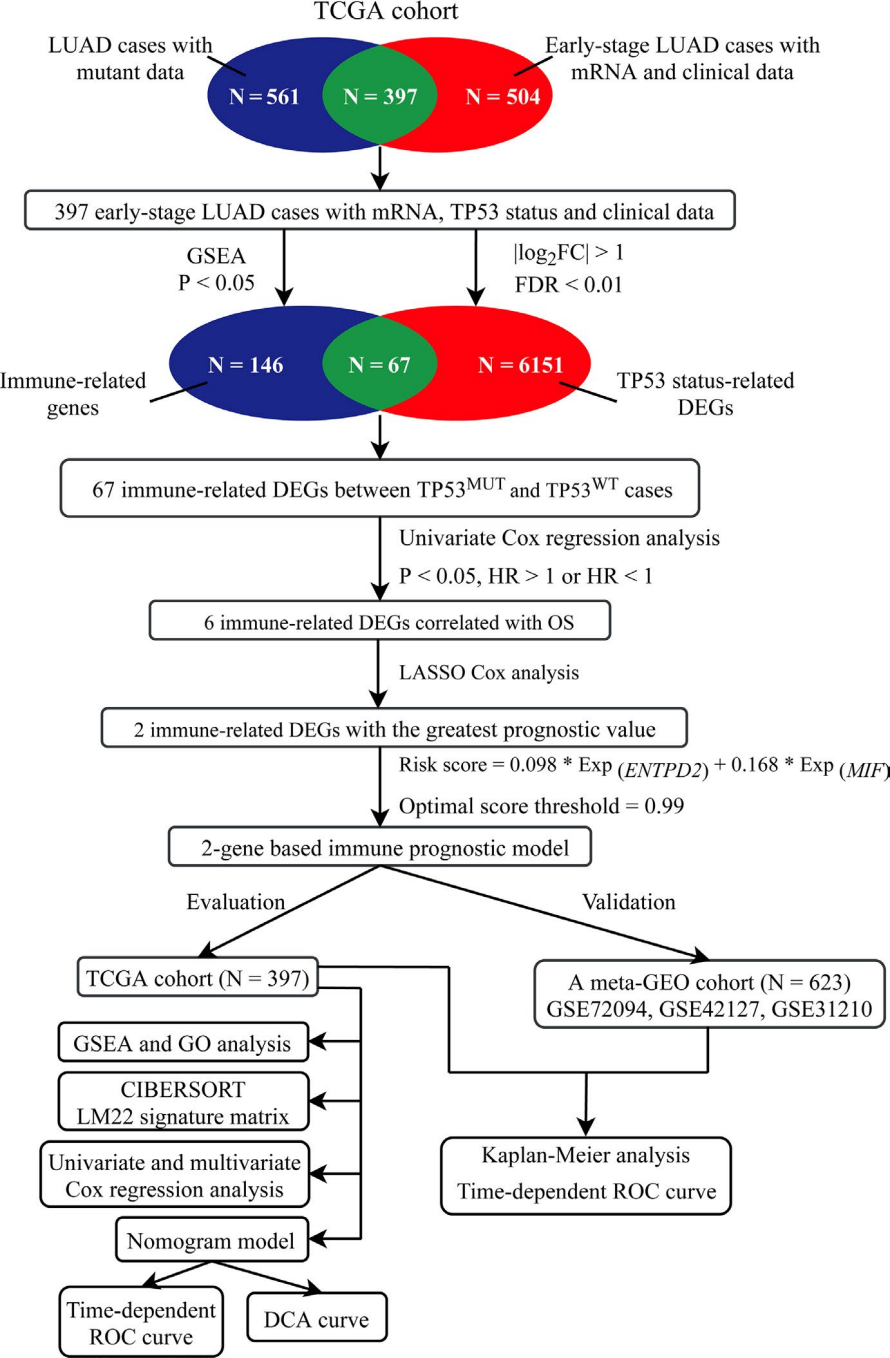
The flow diagram of our study. DEGs, differently expressed genes; FC, fold change; FDR, false discovery rate; LASSO, least absolute shrinkage and selection operator; ROC, receiver operating characteristic; OS, overall survival

We retrospectively analyzed the raw data of somatic single nucleotide mutation, mRNA expression, and corresponding clinical information of early‐stage LUAD patients from four public datasets, including The Cancer Genome Atlas (TCGA) database and three independent datasets retrieved from Gene Expression Omnibus (GEO; GSE72094 based on platform GPL15048, GSE42127 based on platform GPL6884 and GSE31210 based on platform GPL570). Only early‐stage LUAD cases with sufficient clinical annotation were incorporated into our study. Certain clinicopathological variables, such as age at diagnosis, gender, race, TP53 mutant information, status of residual tumor, TNM stage, survival time, and survival status, were extracted for each patient. A total of 1020 early‐stage LUAD patients were included, consisting of 397 cases from TCGA, 336 cases from GSE72094, 111 cases from GSE42127, and 176 cases from GSE31210. We selected the TCGA dataset as independent training cohort. The remaining three GSE datasets were merged into one meta‐GEO as validation cohort. Both gene expression data and clinicopathological information for early‐stage LUAD are publicly available. All analyses in our study were performed strictly followed to the guidelines and regulations of above databases. The flow chart of overall study design was illustrated in Figure 1.

Specifically, the somatic mutation status of 561 LUAD cases (workflow type: SomaticSniper Variant Aggregation and Masking) and RNA sequencing (RNA‐seq) data and the corresponding clinicopathologic parameters of 504 early‐stage LUAD samples were extracted from the TCGA (https://tcga‐data.nci.nih.gov/tcga/; up to May 30th, 2020).^35^ The gene symbols were annotated using “clusterProfiler” R package.^36^ Of above early‐stage LUAD patients, a total of 397 cases with mRNA expression profiles, TP53 mutant status and clinical data were selected for subsequent analysis in our study. The RNA‐seq data were handled through log2‐scale transformation and was further applied to trimmed mean of M values (TMM) normalization via “edgeR” R package.^37^ Average expression value was estimated and utilized when multiple expression values of the same gene were detected.^35^

Microarray data and the corresponding clinical information of early‐stage LUAD individuals from GSE72094, GSE42127, and GSE31210 were downloaded from Gene Expression Omnibus (GEO) database (http://www.ncbi.nlm.nih.gov/geo/) through “GEOquery” R package.^38^ To accord with standard normal distribution, these data were eliminated batch effects via “sva” R package and normalized by scale function of “limma” R package, which were further merged into a meta‐GEO cohort (including 623 early‐stage LUAD patients) to externally validate the practicability of the IPM.^35^

### 2.2 Gene set enrichment analysis

To investigate the underlying association between TP53 mutant status and immune‐associated biological pathways in early‐stage LUAD, gene set enrichment analysis (GSEA) was conducted between early‐stage LUAD cases without (*n* = 218) and with (*n* = 179) TP53 mutation in the TCGA cohort through GSEA software downloaded from https://www.broadinstitute.org/gsea/.^39^ *p* < 0.05 was considered statistically significant.

### 2.3 Differentially expressed genes analysis based on TP53 status

DEGs between TP53^MUT^ and TP53^WT^ early‐stage LUAD were analyzed by “limma” R package.^40^ |log_2_^fold change (FC)^| > 1 and false discovery rate (FDR) < 0.01 were considered as the cutoff values to identify DEGs. All TP53‐related DEGs and above immune‐related genes obtained from GSEA were overlapped to acquire immune‐associated DEGs between TP53^WT^ and TP53^MUT^ early‐stage LUAD individuals.

### 2.4 Construction and validation of an immune prognostic model

A total of 397 early‐stage LUAD patients in the TCGA database had sufficient information, including mRNA expression profiles, TP53 mutant status, survival time and survival status. All TP53‐related immune DEGs and the survival data of 397 cases were analyzed by univariate Cox regression analysis utilizing “survival” R package.^41^ When *p* value was below 0.05 and |hazard ratio (HR)|was not equal to 1, the corresponding DEGs were considered as prognostic immune‐related genes, which were all incorporated into subsequent investigation.

LASSO Cox analysis was implemented through “glmnet” R package, further determining the most significantly prognostic immune‐related DEGs.^42^ The penalization coefficient was used for evaluating the weight of model parameters. Nonsignificant indicators were shrunk to zero, while residual DEGs were applied for establishing a prognostic risk score model. Subsequently, an IPM was constructed based on corresponding coefficients of the prognostic DEGs: risk score = β_mRNA1_ * Expr_mRNA1_ + β_mRNA2_ * Expr_mRNA2_ + ⋯ + β_mRNAn_ * Expr_mRNAn_, where Expr is the DEG expression level and β is the LASSO Cox regression coefficient. All early‐stage LUAD cases in the TCGA dataset were stratified into the high‐ or low‐risk group according to the optimal cutoff value identified by X‐tile 3.6.1 software.^43^

To determine the distinguishing capability of the IPM for patients' prognosis, the differences of overall survival (OS) between high‐ or low‐risk group were compared through Kaplan–Meier curves with log‐rank test using “survival” R package.^41^ To evaluate the predictive efficiency of the IPM, the time‐dependent receiver operating characteristic (ROC) curve with area under the curve (AUC) value were developed through “survival ROC” R package.^44^ Similarly, the identical median value and formula of risk score based on the TCGA cohort were further implemented in a meta‐GEO validation group to identify the robustness of our IPM.

### 2.5 Estimation of immune cell type fractions

CIBERSORT, a deconvolution algorithm described by Alizadeh et al, has the capability to quantify cell fractions from abundant tissue gene expression profiles. To detect the relative fractions of 22 infiltrating immune cell types in the early‐stage LUAD samples, the CIBERSORT (http://cibersort.stanford.edu/) and the LM22 were utilized.^45^ LM22, the leukocyte gene signature matrix with 547 genes, is an approach to accurately differentiate 22 human hematopoietic cell phenotypes, including B cells, T cells, natural killer (NK) cells, macrophages, dendritic cells (DCs), and myeloid subsets. CIBERSORT generates a *p* value for the deconvolution of each specimen through Monte Carlo sampling, thus measuring the confidence of the results. When *p* value is below 0.05, the outcome of the concluded fractions of immune cell populations generated through CIBERSORT is considered receivable.^45^ Therefore, in our report, CIBERSORT integrated with LM22 were applied for quantifying the percentage of immune cells in TP53^MUT^ and TP53^WT^ early‐stage LUAD samples in the TCGA database. Patients with a CIBERSORT *p* < 0.05 were selected for subsequent analysis.

### 2.6 Independence of the IPM from conventional clinicopathological factors

A total of 397 early‐stage LUAD cases with sufficient clinicopathological data such as age, gender, race, TP53 mutant status, residual tumor status, TNM stage, and survival data were subjected to subsequent analysis. Univariate and multivariate Cox regression analysis were applied for exploring whether the predictive capacity of an IPM was independent of conventional clinicopathological variables.

### 2.7 Development and validation of nomogram model

We incorporated all statistically significant clinicopathological parameters identified via multivariate Cox analysis and further established a visualized nomogram model through “rms” and “survival” R package, thus predicting the 3‐,5‐,10‐year OS probability of patients.^46^, ^47^ The predictive capability of the nomogram was evaluated through measuring discrimination and calibration utilizing a bootstrap approach under 1000 resampling. Discrimination was assessed via Concordance index (C‐index). The C‐index is closer to 1, implying more accurate predictive ability of the nomogram model.^48^ Calibration curves were applied for evaluating the consistency between the predicted survival probability of nomogram and the actual observed possibility. The 45 reference line indicated the optimal predictive performance.^46^ The ROC curve with AUC value and the decision curve analysis (DCA) were generated through using “survival ROC” and “rmda” R package, respectively, which can evaluate the predictive precision and clinical utility of nomogram.^44^, ^49^

## 3 RESULTS

### 3.1 Correlation between TP53 mutations and immune phenotype in early‐stage LUAD

**Figure 2:**
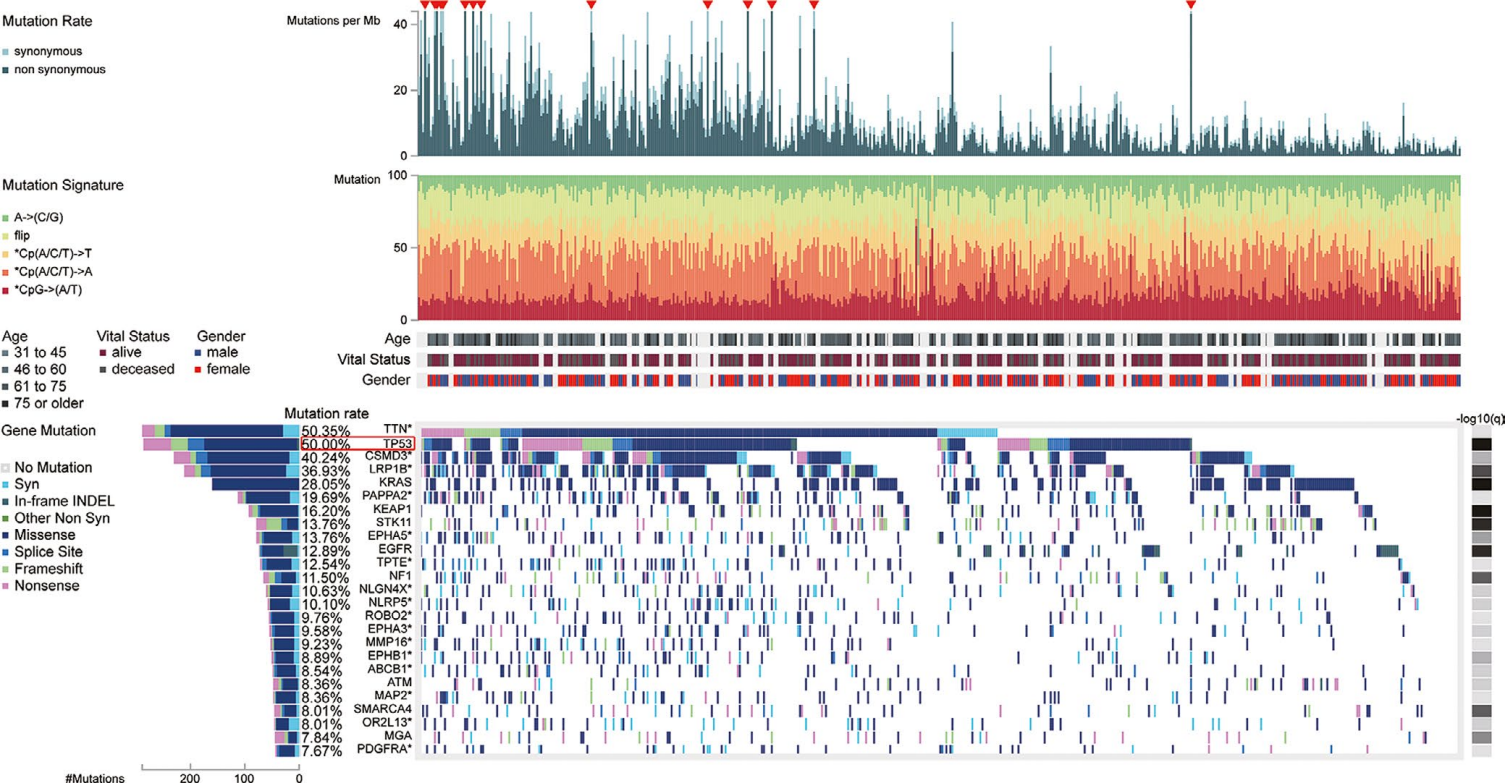
Somatic mutation landscape of lung adenocarcinoma (LUAD) patients in the TCGA database, which was obtained from the Fire Browse platform (http://firebrowse.org/)

As revealed in Figure 2, TTN had the highest mutant frequency (50.35%) in LUAD patients. TP53 was the second most common mutant gene, accounting for approximately 50.00% of LUAD cases, followed by CSMD3 (40.24%), LRP1B (36.93%), KRAS (28.05%) and PAPPA2 (19.69%). TP53 mutations predominantly consisted of missense mutation, nonsense mutation, splice site mutation, and frameshift.

**Figure 3:**
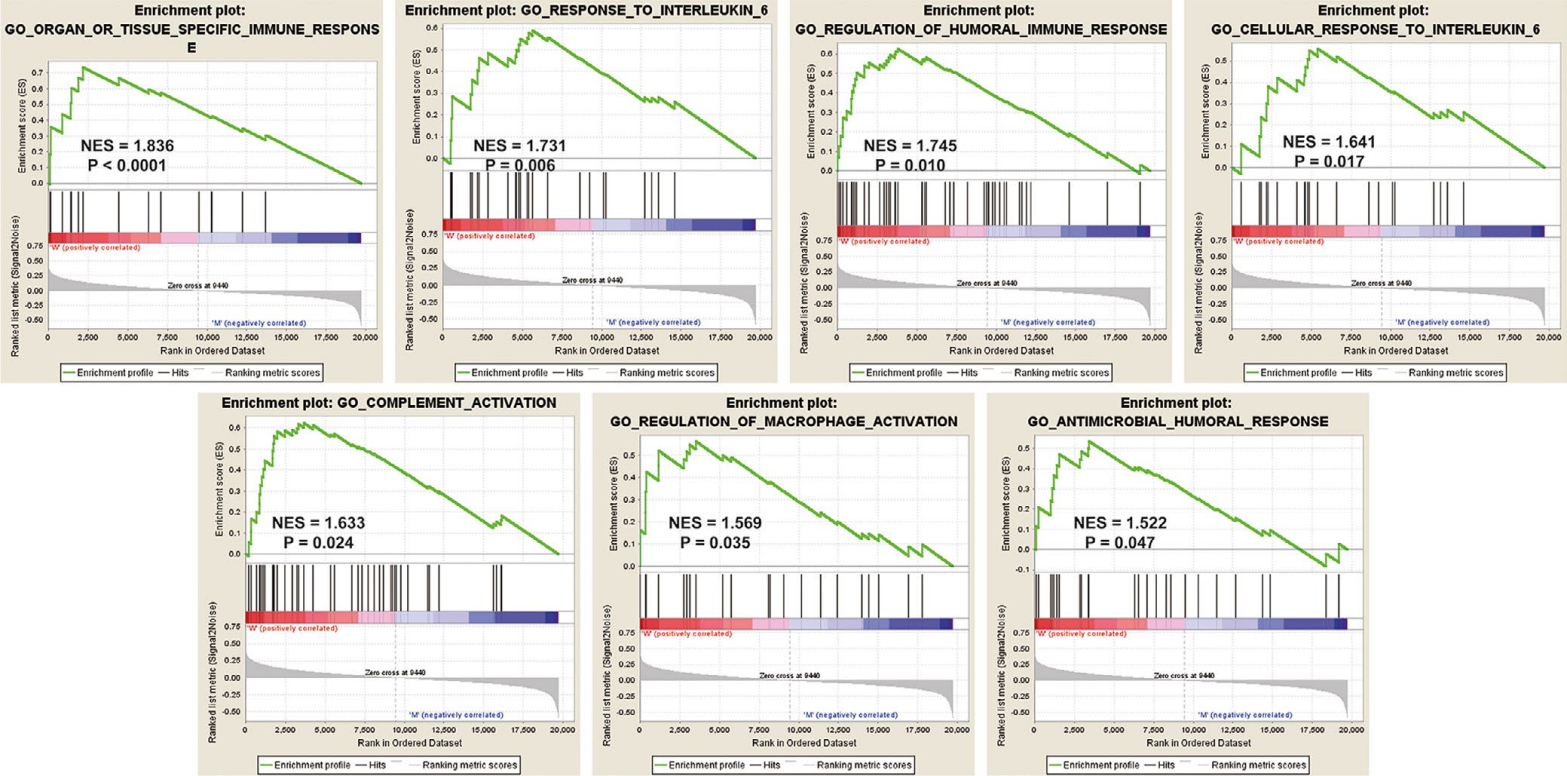
Gene set enrichment analysis (GSEA) results of significant immune‐associated biological processes enriched in TP53^WT^ early‐stage lung adenocarcinoma (LUAD) individuals. TP53 mutated, TP53^MUT^

We further illuminated how the immune‐associated biological processes and genes differ between TP53^MUT^ (*n* = 179) and TP53^WT^ (*n* = 218) early‐stage LUAD patients based on their mRNA expression profiles and clinical data extracted from the TCGA database. GSEA results revealed that seven immune‐associated pathways were significantly enriched in TP53^WT^ early‐stage LUAD, including organ or tissue specific immune response (normalized enrichment score (NES) = 1.836, size = 15, *p* < 0.0001), regulation of humoral immune response (NES = 1.745, size = 47, *p* = 0.010), response to interleukin 6 (IL‐6; NES = 1.731, size = 25, *p* = 0.006), cellular response to IL‐6 (NES = 1.641, size = 21, *p* = 0.017), complement activation (NES = 1.633, size = 44, *p* = 0.024), regulation of macrophage activation (NES = 1.569, size = 24, *p* = 0.035), antimicrobial humoral response (NES = 1.522, size = 25, *p* = 0.047; Figure 3; Table S1). Conversely, GSEA results based on hallmark gene sets further demonstrated that there were no statistically significant immune‐associated biological processes enriched in TP53^MUT^ early‐stage LUAD patients. Several cell cycle‐associated signaling pathways were significantly enriched in early‐stage LUAD with mutated TP53, such as chromosome segregation, mitotic nuclear division, DNA replication, cell cycle G2/M phase transition and DNA repair (*p* < 0.05; Table S2).

### 3.2 Immune‐associated DEGs between TP53^WT^ and TP53^MUT^ early‐stage LUAD

A total of 146 immune‐associated genes were acquired from above seven immune‐related pathways. Simultaneously, there were 6151 TP53 status‐associated DEGs, including 1856 downregulated DEGs and 4295 upregulated DEGs in TP53^WT^ early‐stage LUAD (FDR < 0.01 and |log_2_FC| > 1). Consequently, of the 6151 DEGs investigated, 67 immune‐associated DEGs between TP53^MUT^ and TP53^WT^ early‐stage LUAD were determined through the overlapping analysis (Table S3).

### 3.3 Establishment of an IPM based on the TCGA database

To investigate the prognostic role of 67 immune‐associated DEGs in patients, we performed univariate Cox analysis and further found that six of 67 DEGs, including C8B, C6, PLA2G1B, Ectonucleoside triphosphate diphosphohydrolase family member 2 (ENTPD2), Macrophage migration inhibitory factor (MIF) and C8A, were significantly related to patients' OS (*p* < 0.05; Table 1). Moreover, LASSO Cox analysis was conducted to identify two immune‐associated DEGs (ENTPD2 and MIF) with the optimal prognostic efficiency in patients. In the TCGA cohort, ENTPD2 (HR = 1.12, 95% CI: 1.02–1.24, *p* = 0.0243) and MIF (HR = 1.21, 95% CI: 1.02–1.43, *p* = 0.0277) were considered as risky genes in early‐stage LUAD and were ultimately chosen to establish an IPM (Table 1). The following formula can be used to calculate the risk score of each patient:IPM risk score = 0.098 * ENTPD2 expression value +0.168 * MIF expression value. We estimated the risk score of every patient and identified optimal cutoff value via X‐tile software.

**Table 1:** Univariate Cox analysis of immune‐associated DEGs between TP53^WT^ and TP53^MUT^ early‐stage LUAD

Gene	HR (95% CI)	*p* value
*C8B*	0.92 (0.86–0.98)	0.0105
*C6*	0.91 (0.85–0.98)	0.0138
*PLA2G1B*	0.92 (0.86–0.99)	0.019
***ENTPD2***	**1.12 (1.02–1.24)**	**0.0243**
***MIF***	**1.21 (1.02–1.43)**	**0.0277**
*C8A*	0.91 (0.84–0.99)	0.0358

Bold highlights the significant genes or parameters which are emphasized and discussed in the main manuscript.

Abbreviations: TP53^MUT^, TP53 mutated; TP53^WT^, TP53 wild‐type.

**Figure 4:**
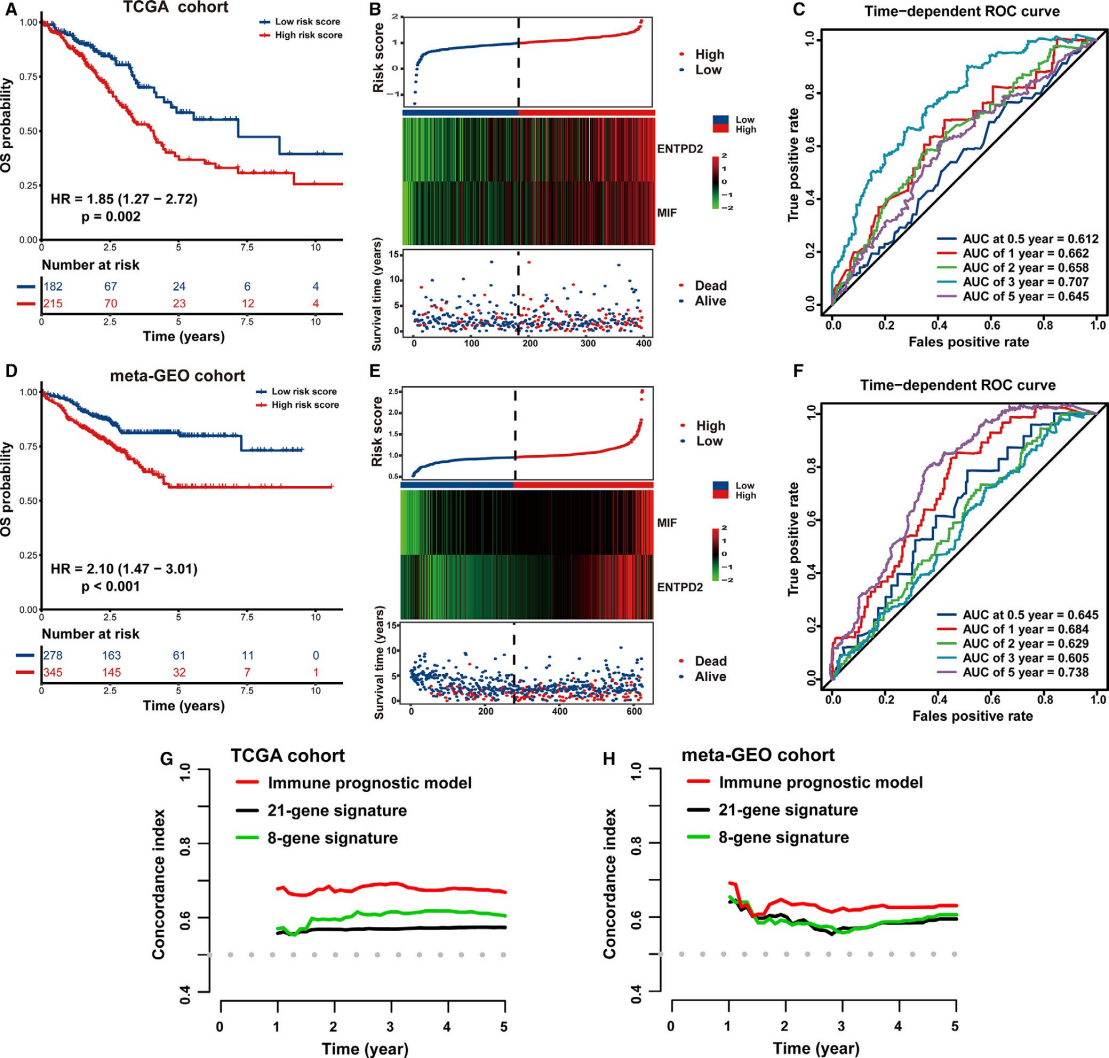
Prognostic analysis of an immune prognostic model (IPM). All early‐stage lung adenocarcinoma (LUAD) cases were stratified into high‐ and low‐risk groups based on median risk score in (A–C) the TCGA training cohort and (D–F) the meta‐GEO testing cohort. (A and D) Kaplan–Meier curves revealed that individuals with high risk score displayed remarkably diminished overall survival (OS) than those with low risk score. (B and E) The risk score distribution was consistent with the heatmap of two immune‐related prognostic genes' expression and survival status of cases. The black dotted line signals the cutoff of IPM risk score to stratify cases into low‐ and high‐risk groups. (C and F) Time‐dependent ROC curves of the IPM demonstrated the relatively satisfactory predictive performance. (G and H) Prognostic prediction efficiency was evaluated between an IPM and 21‐gene signature as well as 8‐gene signature through estimating the C‐index in both cohorts

All 397 early‐stage LUAD cases were stratified into high‐ or low‐risk group in accordance with the optimal cutoff point (0.99). Survival analysis revealed that a higher risk score was correlated with significantly worse prognosis of cases in the TCGA training database (HR = 1.85, 95% CI: 1.27–2.72, *p* = 0.002; Figure 4A). The risk score distribution, heatmap concerning two immune genes’ expression, and survival status for every patient were illustrated in Figure 4B. There was a high uniformity between the expression levels of ENTPD2 and MIF and the IPM risk score. Specifically, the high‐risk cases displayed significantly enhanced levels of ENTPD2 and MIF compared with their low‐risk counterparts. The time‐dependent receiver operating characteristic (ROC) curve and the corresponding area under the ROC curves (AUC) was applied for evaluating the prognostic accuracy of our IPM. The AUC values for OS of early‐stage LUAD were 0.612 (95% CI: 0.466–0.736) at 0.5 years, 0.662 (95% CI: 0.594–0.751) at 1 years, 0.658 (95% CI: 0.598–0.721) at 2 years, 0.707 (95% CI: 0.646–0.763) at 3 years and 0.645 (95% CI: 0.543–0.738) at 5 years (Figure 4C).

### 3.4 Validation of an IPM in a meta‐GEO cohort

A meta‐GEO cohort composed of 623 early‐stage LUAD cases was utilized to externally validate the robustness of our IPM. The expression values of ENTPD2 and MIF gene in the meta‐GEO validation cohort were normalized, with an average value of 0 and a standard deviation (SD) of 1.^50^ On the basis of the identical score formula and cutoff point in the TCGA training group, all cases in a meta‐GEO group were categorized into high‐ or low‐risk groups. As revealed in Figure 4D, cases with high risk score were characterized with an inferior prognosis compared with those in the low‐risk group (HR = 2.10, 95% CI: 1.47–3.01, *p* < 0.001), which was roughly consistent with the results in the TCGA group. Figure 4E illustrated the IPM score, gene expression and survival status of early‐stage LUAD cases in the meta‐GEO validation cohort. Moreover, the diagnostic performance of the IPM attained an AUC of 0.645 (95% CI: 0.552–0.715) at 0.5 years, 0.684 (95% CI: 0.612–0.749) at 1 years, 0.629 (95% CI: 0.515–0.633) at 2 years, 0.605 (95% CI: 0.539–0.645) at 3 years and 0.738 (95% CI: 0.637– 0.758) at 5 years (Figure 4F). Therefore, these findings highlight that the IPM is robust and applicable in varying populations and platforms.

Recently, Zuo et al. established a novel 8‐gene prognostic signature for predicting the long‐term OS of patients with early‐stage non‐small‐cell lung cancer (NSCLC).^51^ Firstly, they investigated four lung cancer datasets to estimate DEGs between early‐stage NSCLC and normal adjacent lung tissue. Furthermore, Cox proportional hazards models was performed on those DEGs to determine the candidate prognostic genes. Ultimately, a risk score model for these hub genes was developed in the TCGA cohort. Similarly, in 2020, Liang et al also proposed a promising 21‐gene‐based prognostic immune prediction model for early‐stage LUAD patients in the TCGA cohort.^52^ The C‐index, a frequently utilized evaluation indicator for survival models, was estimated through “pec” R package utilizing a bootstrap manner under 1000 resampling, thus comparing the predictive performance of the previously published prognostic signatures and our IPM. A higher C‐index value indicates the more favorable predictive performance of the model.^53^ The C‐index of our IPM to predict 1 to 5‐year OS surpassed that of the 8‐gene and 21‐gene prognostic signatures in TCGA and meta‐GEO group, highlighting that the IPM displayed relatively desirable performance to predict the prognosis of early‐stage LUAD patients (Figure 4G,H).

### 3.5 Survival analysis of the IPM based on TP53 status

**Figure 5:**
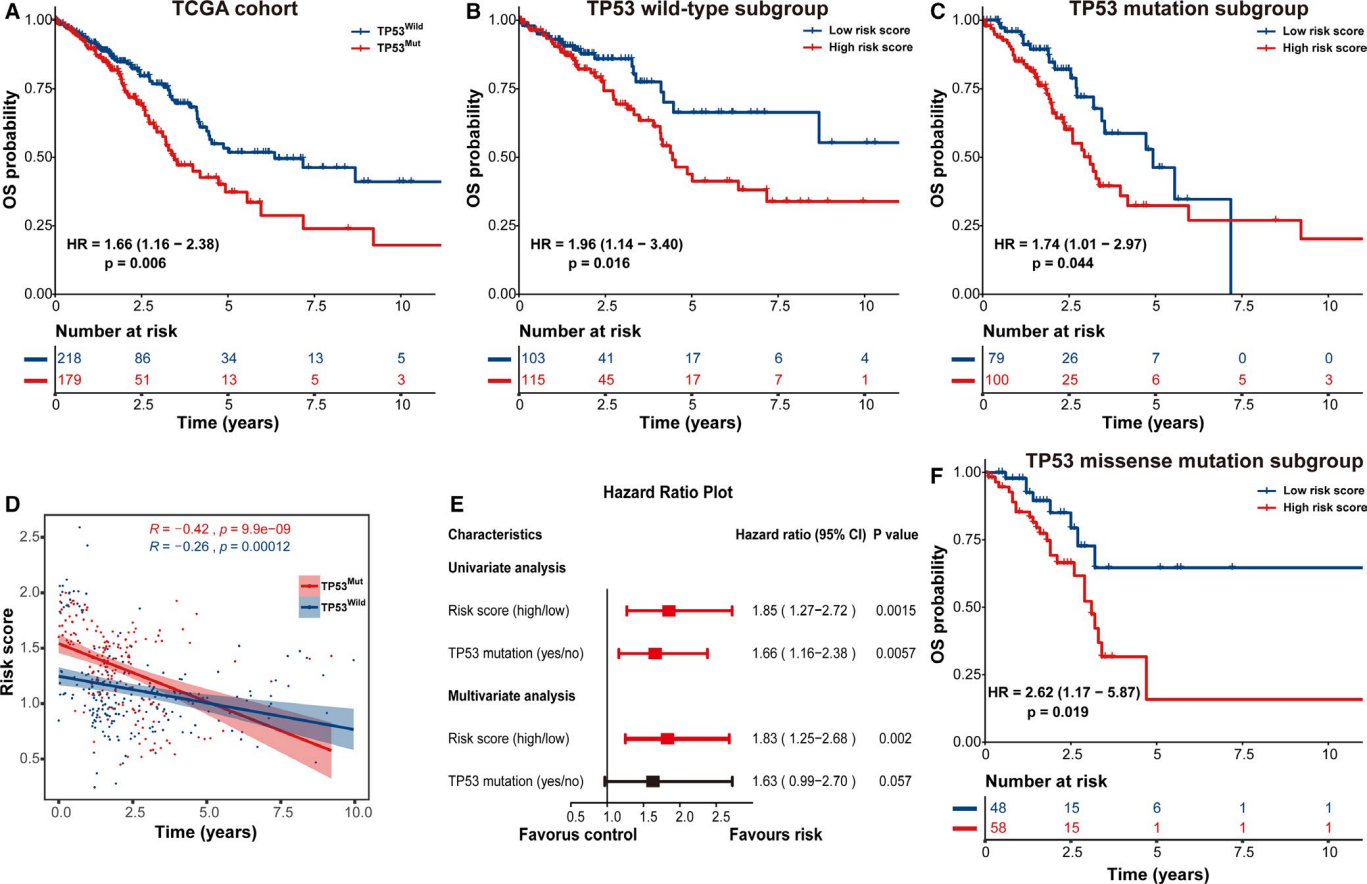
Prognostic analysis of early‐stage lung adenocarcinoma (LUAD) patients in multiple subgroups stratified by TP53 status. Kaplan–Meier curves of the overall survival (OS) difference between high‐ and low‐risk cases in (A) the whole TCGA cohort, (B) TP53^WT^ subgroup and (C) TP53^MUT^ subgroup. (D) Correlation analysis between immune prognostic model (IPM) risk score and patient's OS. (E) Univariate and multivariate Cox regression analysis of the prognostic association between an IPM and TP53 status. (F) Kaplan–Meier curves of the OS difference between high‐ and low‐risk patients in the TP53 missense mutation subgroup. TP53^MUT^, TP53 mutated; TP53^WT^, TP53 wild‐type

As revealed in Figure 5A, TP53 mutant early‐stage LUAD patients exhibited a 1.66‐fold higher risk (HR = 1.66, 95% CI: 1.16–2.38, *p* = 0.006) than patients without TP53 mutation. We stratified all cases in the TCGA group into two subgroups based on TP53 status, thus uncovering whether the predictive power of the IPM was independent of TP53 status. The results indicated that high risk score exerted a significantly negative effect on the prognosis of early‐stage LUAD patients in TP53^WT^ subgroup (HR = 1.96, 95% CI: 1.14–3.40, *p* = 0.016; Figure 5B) and TP53^MUT^ subgroup (HR = 1.74, 95% CI: 1.01–2.97, *p* = 0.044; Figure 5C), respectively. Similarly, correlation analysis also demonstrated that there was a negative association between IPM risk score and survival time in both TP53^WT^ (*R* = −0.26, *p* = 0.00012) and TP53^MUT^ (*R* = −0.42, *p* = 9.9e‐09) subgroups (Figure 5D). Furthermore, the predictive performance of our IPM was independent of TP53 status via univariate and multivariate Cox analysis (Figure 5E). Concerning that a majority of TP53 alterations were missense mutation (106/179) in our report, we then investigated whether the IPM influenced the prognosis of patients in different TP53 mutation subtypes. As revealed in Figure 5F, cases with high risk score were correlated with diminished survival in the TP53 missense mutation subgroup (HR = 2.62, 95% CI: 1.17–5.87, *p* = 0.019).

### 3.6 Strengthened immune response in early‐stage LUAD patients with low IPM risk score

Gene set enrichment analysis was further conducted to disclose immune‐related processes between low‐risk (*n* = 182) and high‐risk (*n* = 215) early‐stage LUAD cases in the TCGA training cohort. Cases with low risk score were significantly related to several immunological pathways, including regulation of adaptive immune response (NES = 1.576, size = 33, *p* = 0.041), cytokine production involved in immune response (NES = 1.573, size = 16, *p* = 0.034), regulation of production of molecular mediator of immune response (NES = 1.554, size = 26, *p* = 0.037), and thymic T‐cell selection (NES = 1.549, size = 19, *p* = 0.048; Table S4). Conversely, there was no statistically significant immune‐associated biological processes enriched in patients with high risk score (Table S5). Thus, low IPM risk score potentially portends an enhanced immune phenotype while attenuated immune response is prone to occur in those cases with high risk score.

### 3.7 Immune landscapes between early‐stage LUAD patients with low and high risk score

**Figure 6:**
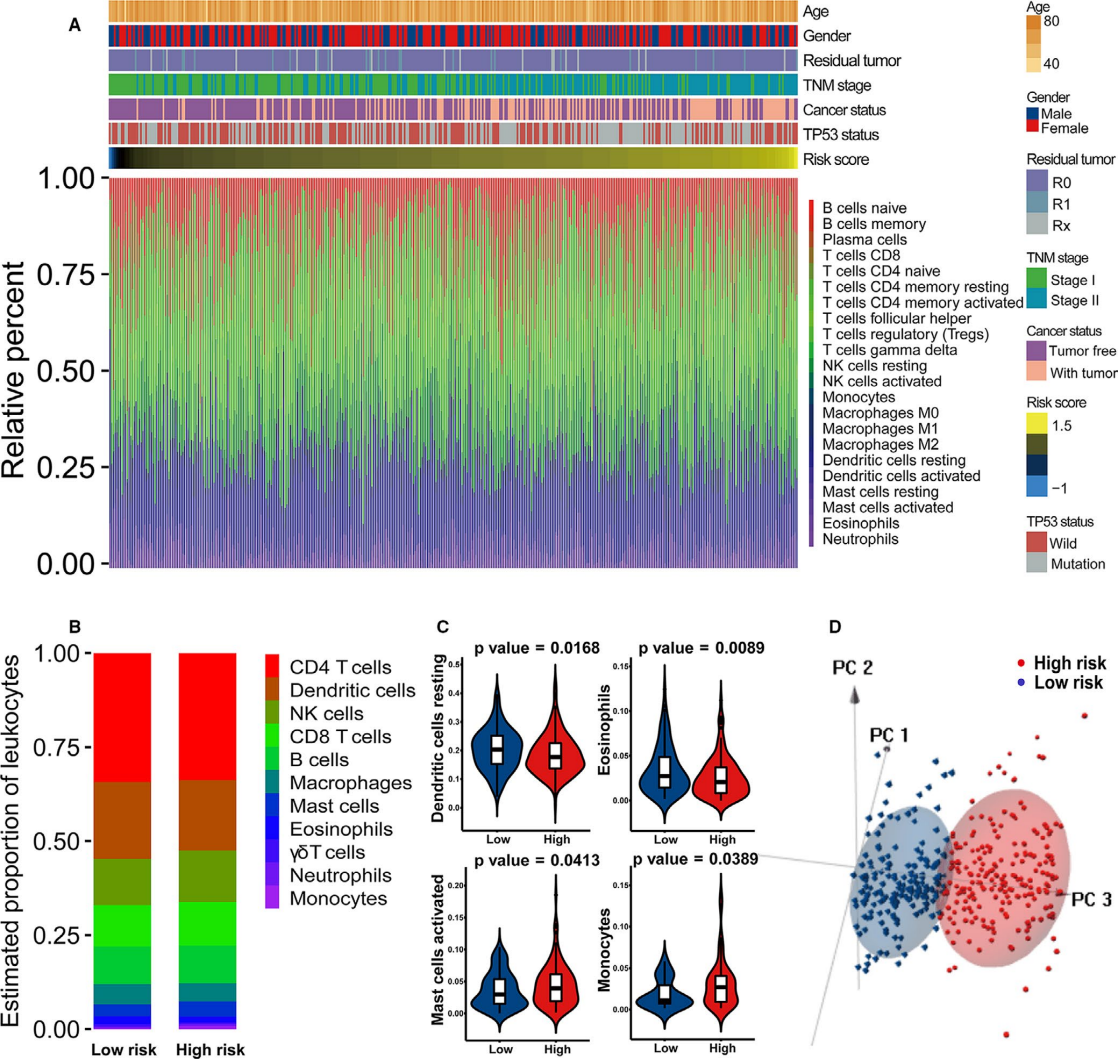
Distribution of the immune prognostic model (IPM) risk score, clinical characteristics and immune cell subpopulations of early‐stage lung adenocarcinoma (LUAD) patients in the TCGA cohort. (A) Heatmaps summarizing the fraction of intra‐tumoral immune cells and clinical characteristics between high‐ and low‐risk patients. (B) Bar charts of immune cell subset proportions between high‐ and low‐risk patients. (C) Violin plots of significantly different fractions of immune cells between high‐ and low‐risk patients. (D) The principal component analysis (PCA) based on immune cells subpopulations presenting a significant distinction between high‐ and low‐risk cases

The discrepancies concerning the compositions of 22 tumor‐infiltrating immune cells between low‐ and high‐risk early‐stage LUAD cases were evaluated through CIBERSORT combined with LM22 signature matrix. The distribution of the risk score, clinicopathologic characteristics and immune cell composition were evaluated in the TCGA cohort. A higher risk score was significantly correlated with more advanced TNM stage and the status of with tumor (Figure 6A). Additionally, the proportions of immune cells were diverse among different early‐stage LUAD samples, highlighting that alterations of intra‐tumoral immune cells potentially serve as inherent characteristics to represent individual variations (Figure 6A). Generally, the five most common immune cell fractions in early‐LUAD tissues were CD4^+^ T cells, DCs, NK cells, CD8^+^ T cells and B cells, and the sum of their average proportions was over 50% (Figure 6B). As revealed in Figure 6C, patients with low risk score were characterized with an increased proportion of resting DCs (*p* = 0.0168) and eosinophils (*p* = 0.0089). Conversely, cases in the high‐risk group had higher infiltration of activated mast cells (*p* = 0.0413) and monocytes (*p* = 0.0389). We performed principal components analysis (PCA) to identify the difference between low‐ and high‐risk early‐stage LUAD patients on the basis of above‐identified immune cell subpopulations. As showed in Figure 6D, immune cell subpopulations stratified high‐risk and low‐risk cases into two discrete sections, emphasizing that the immune landscape of early‐stage LUAD cases in the low‐risk group was greatly different from those with high risk score. The heterogeneity of immune microenvironment in early‐stage LUAD potentially exerts significant implications for identifying prognosis.

**Figure 7:**
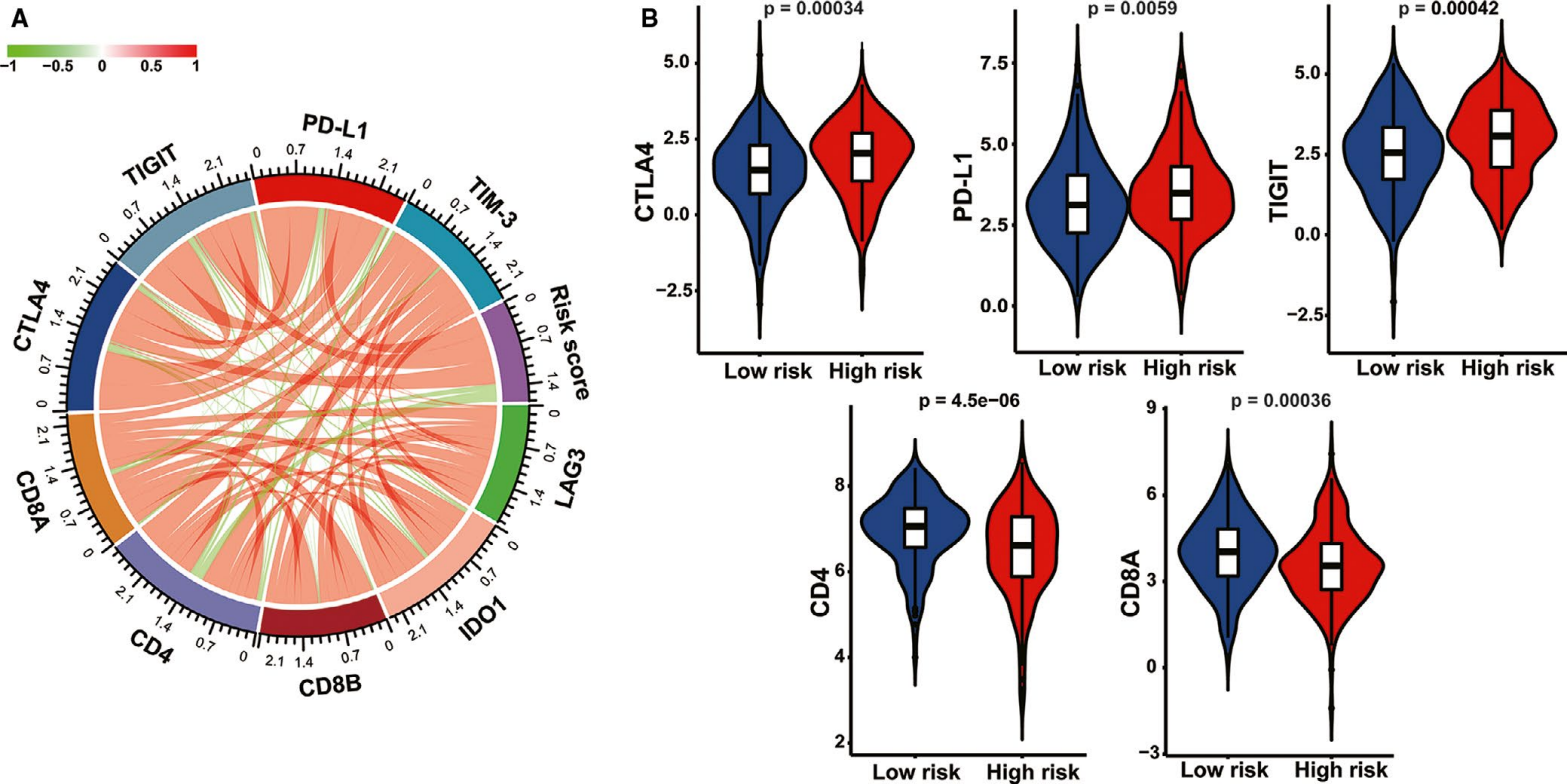
Correlation analysis between the immune prognostic model (IPM) and pivotal immunotherapy‐associated moleculars. (A) Circular plot of the correlation between the IPM risk score and the expression of immune checkpoint regulators. (B) Violin plots of immunotherapy‐associated gene levels between high‐ and low‐risk early‐stage lung adenocarcinoma (LUAD) patients

In order to unravel the associations between the IPM risk score and responses to immunotherapy of early‐stage LUAD patients, we specially focused on the relationship between IPM risk score and the levels of crucial immunotherapy‐related genes. There was a significantly negative correlation between risk score and the expression of CD8A (Pearson correlation coefficient [PCC] = −0.138, *p* = 0.0058) and CD4 (PCC = −0.258, *p* = 1.823e‐07). In contrast, the IPM risk score was significantly positively associated with PD‐L1 (also known as CD274, PCC = 0.163, *p* = 0.0011), CTLA‐4 (PCC = 0.141, *p* = 0.005) and TIGIT (PCC = 0.109, *p* = 0.031; Figure 7A; Table 2), whereas there was no significant correlation between risk score and the levels of TIM‐3, LAG3, IDO1, and CD8B (Table 2). Similarly, violin plots depicted in Figure 7B illustrated that cases with low IPM risk score had significantly increased levels of CD4 and CD8A while significantly diminished expression of PD‐L1, CTLA‐4, and TIGIT compared with the high‐risk patients (*p* < 0.05). Thus, the expression levels of pivotal immunotherapy‐associated genes are potentially correlated with the risk stratification of early‐stage LUAD patients.

**Table 2:** Correlation of immunotherapy‐related genes and IPM risk score in early‐stage LUAD patients

Variable 1	Variable 2	Pearson correlation coefficient	*p* value
**Risk score**	***CTLA4***	**0.1406689**	**0.004986**
**Risk score**	***PD‐L1***	**0.1628738**	**0.001127**
Risk score	*TIM−3*	0.1874968	0.1511717
Risk score	*LAG3*	0.0623279	0.2153
**Risk score**	***TIGIT***	**0.1085566**	**0.03058**
Risk score	*IDO1*	−0.1572216	0.061676
**Risk score**	***CD4***	**−0.2581735**	**1.823e‐07**
**Risk score**	***CD8A***	**−0.1381627**	**0.005826**
Risk score	*CD8B*	−0.06999592	0.1639

Bold highlights the significant genes or parameters which are emphasized and discussed in the main manuscript.

Abbreviations: IPM, immune prognostic model; LUAD, lung adenocarcinoma.

### 3.8 Analysis of IPM‐associated biological function and pathway

**Figure 8:**
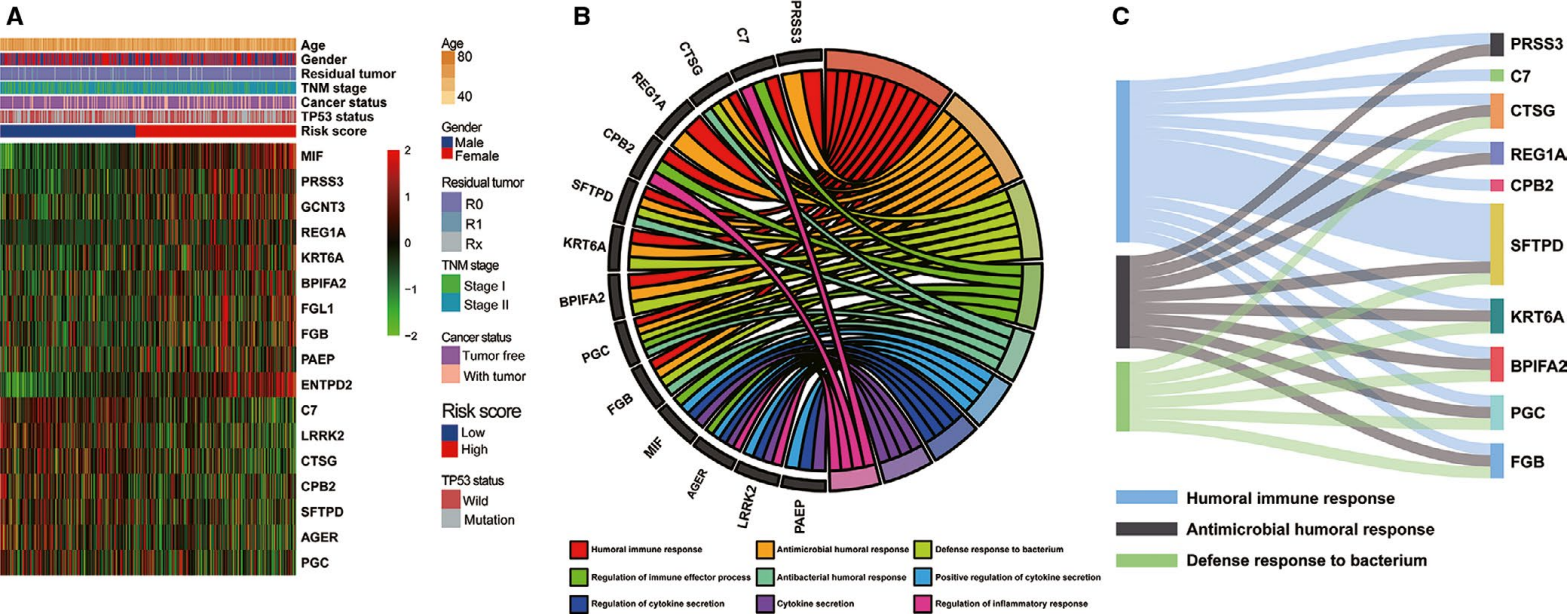
Functional analysis of the immune prognostic model (IPM). (A) Heatmap of risk score‐associated immune DEGs in early‐stage lung adenocarcinoma (LUAD) patients in the TCGA cohort. (B) Circular plot and (C) Sankey plot of the significantly biological pathways enriched in risk score‐associated immune DEGs. DEGs, differently expressed genes

GO analysis was performed to predict the potential biological significance of immune‐associated DEGs between the high‐ and low‐risk groups. A total of 17 immune‐associated DEGs were obtained through overlapping 178 risk score‐associated DEGs and 94 IPM‐related immune genes, which were considered as risk score‐associated immune DEGs (Figure 8A). Those genes were submitted for GO analysis to reveal the underlying biological functions and pathways (Figure 8B,C). These results demonstrated that risk score‐associated immune DEGs in the TCGA cohort were primarily enriched in the humoral immune response, antimicrobial humoral response and positive regulation of cytokine secretion (Table S6).

### 3.9 The IPM is independent of traditional clinicopathological factors

**Figure 9:**
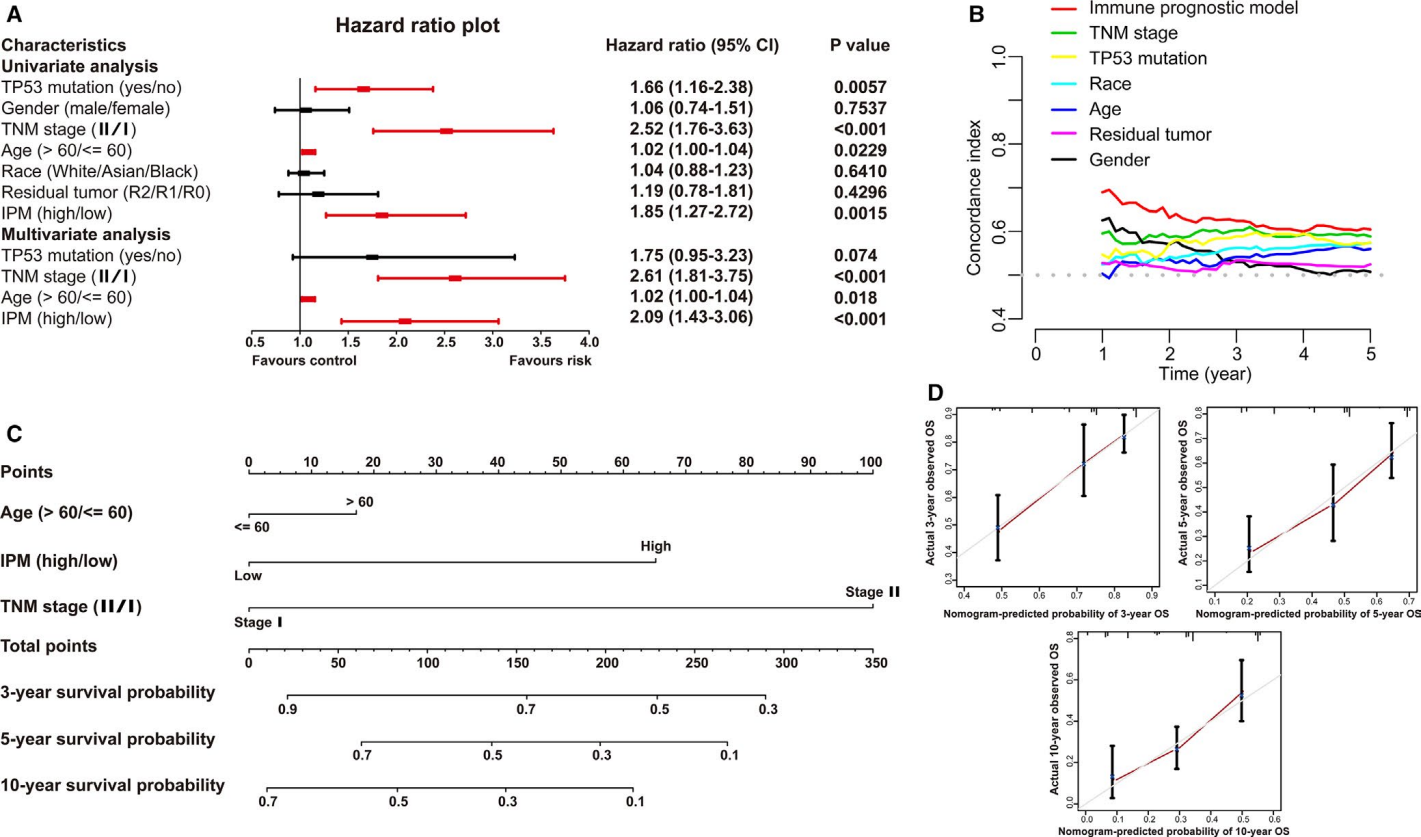
Association between the immune prognostic model (IPM) and other clinicopathologic variables of early‐stage lung adenocarcinoma (LUAD) patients in the TCGA cohort. (A) Forest plot of univariate and multivariate Cox regression analysis to assess the association between IPM and conventional prognostic factors. (B) The C‐indexes were estimated to compare the prognostic efficiency between the IPM and other traditional clinical variables. (C) Nomogram for predicting 3‐, 5‐, and 10‐year OS for early‐stage LUAD patients. (D) Calibration curve of nomogram concerning consistency between predicted and observed 3‐, 5‐, and 10‐year outcomes in the TCGA cohort. Gray line at 45° represents perfect prediction, and red line represents the actual performances of the nomogram. C‐index, Concordance index

Univariate and multivariate Cox regression analysis were applied for assessing the contribution of an IPM as an independent prognostic parameter to the OS of early‐stage LUAD cases in the TCGA cohort. As revealed in Figure 9A, univariate Cox analysis demonstrated that several clinicopathologic parameters, such as TP53 status, TNM stage, age and IPM risk score, exerted an effect on the prognosis of early‐stage LUAD cases. Above significant variables were incorporated into the following multivariate Cox regression analysis. The findings demonstrated that TNM Ⅱ stage (HR = 2.61, 95% CI: 1.81–3.75, *p* < 0.001), high risk score (HR = 2.09, 95% CI: 1.43–3.06, *p* < 0.001) and age over 60 years (HR = 1.02, 95% CI: 1.00–1.04, *p* = 0.018) were adverse prognostic factors in early‐stage LUAD (*p* < 0.05). Altogether, these results indicated that the risk score was correlated with OS and the predictive capability of the IPM was independent of traditional clinicopathological variables for the prognosis of early‐stage LUAD cases.

Moreover, we made a comparison of the C‐index between our IPM and traditional clinicopathological factors. Among six prognosis‐predictive parameters (including TP53 mutant status, gender, TNM stage, age, race and residual tumor), our IPM displayed the greatest average C‐index (0.625) than other traditional clinicopathological parameters (0.509 to 0.579; Figure 9B). Thus, above findings suggested a more satisfactory capability of the IPM to estimate the OS of early‐stage LUAD cases.

### 3.10 Construction and identification of a nomogram model based on the IPM

A nomogram model was further developed through integrating all significant parameters (the IPM, TNM stage and age) identified via multivariate Cox analysis, which can confer physicians with a quantitative method to estimate the survival of early‐stage cases. In the nomogram model, above parameters were assigned scores based on a point scale with a range of 0 to 100. The score of each parameter was identified through plotting upward a straight line. The scores of the parameters of each patient were added up and considered as the total points. For each early‐stage LUAD patient, the survival rate of 3, 5, and 10 years was estimated through plotting downward a perpendicular line from the total point axis to the result axis. Specifically, one early‐stage LUAD patient at TNM stage Ⅱ (100 points), with high risk (66 points) and age over 60 years (17 points) obtained a total point of 183. The perpendicular line plotted from the total point axis at a numeric value of 183 to the result axis revealed that the survival probability of 3, 5, and 10 years was 63%, 35% and 19%, respectively. Thus, TNM stage made the greatest contributions to risk points with a range of 0 to 100, followed by IPM risk score (ranging from 0 to 66) and age (ranging from 0 to 17; Figure 9C).

**Figure 10:**
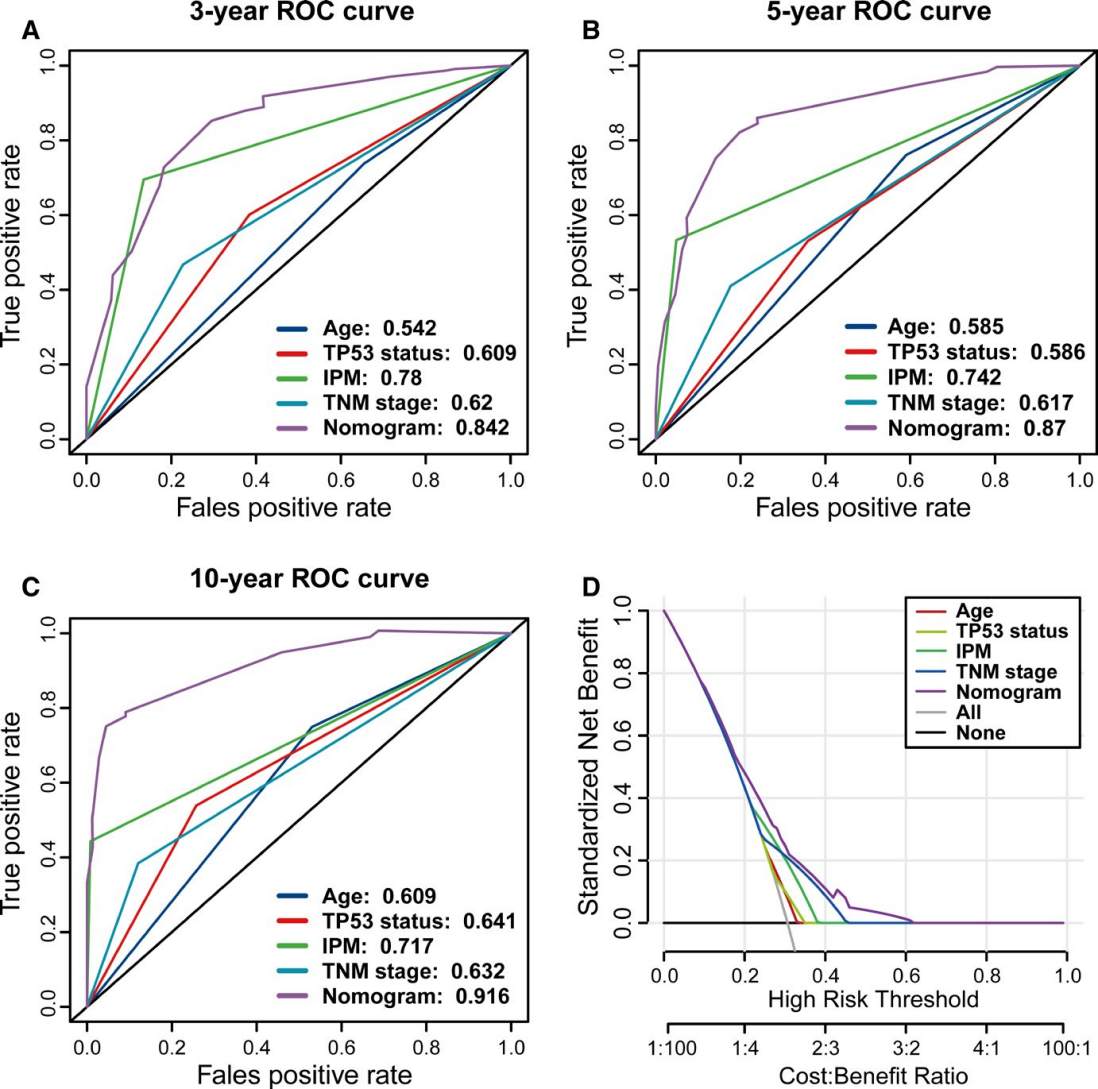
The predictive efficiency and clinical utility were compared between nomogram and other clinicopathologic characteristics of early‐stage lung adenocarcinoma (LUAD) patients in the TCGA cohort. The time‐dependent receiver operating characteristic (ROC) curves of the nomogram model for (A) 3‐year, (B) 5‐year, and (C) 10‐year overall survival (OS) of patients. (D) The decision curve analysis (DCA) curves of the nomogram model for patients' survival

The nomogram displayed a relatively desirable C‐index of 0.709 (95% CI: 0.615–0.779). The deviation correction line was close to the 45‐degree reference line in the calibration curves, indicating the satisfactory consistency between model prediction and practical observation in 3‐, 5‐, and 10‐year OS of patients (Figure 9D). We made a contrast of the predictive efficiency of nomogram model and additional clinical variables (such as age, TP53 status and IPM as well as TNM stage) through establishing time‐dependent ROC curves. Concerning the ROC curve of 3‐year OS, nomogram model achieved the highest AUC of 0.842, followed by IPM (AUC = 0.78), TNM stage (AUC = 0.62), TP53 status (AUC = 0.609) and age (AUC = 0.542; Figure 10A). The nomogram to predict 5‐year OS achieved an AUC value of 0.87, which was more satisfactory than that of IPM (0.742), TNM stage (0.617), TP53 status (0.586) and age (0.585; Figure 10B). The AUC for the nomogram, IPM, TNM stage, TP53 status and age to predict 10‐year OS were 0.916, 0.717, 0.632, 0.641 and 0.609, respectively (Figure 10C). Furthermore, DCA curve was established to evaluate the clinical utility and benefit of the nomogram and additional parameters. The DCA of the nomogram displayed the greatest net benefits, followed by TNM stage, IPM, TP53 status and age (Figure 10D). Collectively, above findings highlighted that the nomogram, constituted by IPM, TNM stage and age, is an optimal model with relatively desirable clinical utility to predict long‐term OS of early‐stage LUAD.

## 4 DISCUSSION

A growing number of publications have demonstrated the complicated interaction between the immune microenvironment and the malignant transformation and development of LUAD.^54^ TP53 mutant status is associated with the percentages of immune cell infiltration and the expression levels of immune checkpoints, which potentially serves as an indicator for evaluating the effectiveness of immunotherapy in LUAD.^55^, ^56^ Thus, reliable prognostic biomarkers associated with the tumor immune landscape and TP53 status potentially hold great promise for recognizing promising molecular targets and strengthening patient management in the era of immunotherapy.

In our report, we focused on the effect of TP53 mutation on modulating the immune landscape in early‐stage LUAD based on bioinformatic approaches. Initially, we demonstrated that TP53^WT^ early‐stage LUADs displayed a distinctly intensive local immune response compared with TP53^MUT^ counterparts through GSEA analysis. Our conclusion is supported by another report where revealed that TP53^WT^ female LUADs rather than TP53^MUT^ cases were correlated with the longest survival and the increased percentages of certain immune infiltrates, such as INF‐γ, TNF and macrophages monocytes. While immunosuppressive molecule PD‐L1 levels were greater in TP53^MUT^ LUAD compared with TP53^WT^ counterpart.^57^, ^58^ Furthermore, we established a prognostic signature based on two immune‐associated DEGs (MIF and ENTPD2) whose expression was related to the TP53 status and the prognosis of early‐stage LUAD. Our IPM can further classify clinically defined groups of early‐stage LUAD patients into subgroups with different survival outcomes. High expression levels of MIF and ENTPD2 signified high IPM risk score, further indicating the undesirable prognosis in early‐stage LUAD.

Migration inhibitory factor is structurally conservative homotrimer in mammals and is considered as well‐acknowledged functional immune cytokine with a crucial effect on host immune system and whole inflammatory cascade. MIF is primarily derived from multiple immune cells, such as macrophages monocytes, T and B cells, eosinophils, neutrophils and mast cells.^59^, ^60^, ^61^, ^62^ MIF is initially proved to impede the directionless migration of macrophages in vitro to characterize delayed type hypersensitivity.^63^, ^64^ MIF can also stimulate the secretion of pro‐inflammatory mediators (including TNF‐α, IL‐1β, IL‐6, COX2, and IFN‐γ).^65^ MIF has been demonstrated to be significantly upregulated in various tumors, including colon cancer,^66^ prostate cancer,^67^ malignant melanoma,^68^ head and neck cancer,^69^ glioblastoma^70^ and breast cancer^71^ as well as lung cancer.^72^, ^73^, ^74^, ^75^, ^76^, ^77^ High expression level of MIF usually indicates advanced cancer phenotype and unsatisfactory prognosis in above tumors. Specifically, the lung cancer cells with resistance to cisplatin facilitates M2 polarization of tumor‐associated microphages (TAMs) through secreting MIF, thus accelerating angiogenesis and epithelial‐mesenchymal transition (EMT) as well as distant metastasis of lung cancer.^74^ Overexpression of MIF also activates NF‐κB and further upregulates HIF‐1α, thus amplifying proliferation and Warburg effect in lung cancer.^75^ CXCR4/MIF axis positively modulates tumor growth and EMT interaction in NSCLC.^76^ MiR‐608 mitigates tumor migration and invasion via directly targeting MIF in LUAD.^77^ Thus, MIF can develop into an attractive target for pharmacological intervention for the therapy of lung cancer.

ENTPD2 (also named CD39L1 or NTPDase 2), is considered as a pivotal ectoenzyme involved in extracellular ATP hydrolysis.^78^, ^79^ AMP is generated via ENTPD2‐mediated hydrolyzation of extracellular ATP and is further hydrolyzed by CD73 to adenosine that stimulates tumor proliferation and metastasis as well as drug resistance.^79^ ENTPD2 upregulation is demonstrated in papillary thyroid carcinoma‐derived cells, esophageal cancer cells, gliomas cells, and liver cancer cells in contrast to normal cells.^80^, ^81^, ^82^, ^83^ Overexpression of ENTPD2 is also revealed in HCC clinical samples, which also indicates undesirable prognosis for HCC. The hypoxic microenvironment stimulates ENTPD2 overexpression mediated by hypoxia‐inducible factor‐1 (HIF‐1) in liver cancer cells. ENTPD2 transforms extracellular ATP into 5′‐AMP and further suppresses the differentiation of myeloid‐derived suppressor cells (MDSCs), thus facilitating the maintenance of MDSCs and the development of tumor immunosuppressive microenvironment. Depletion of ENTPD2 can impede tumor growth and strengthen the efficiency of immune checkpoint inhibitors.^82^ Thus, ENTPD2 is harnessed by tumor cells to shun immune‐mediated demolition.

We also demonstrated an enhanced immune landscape in the low‐risk group through functional enrichment analysis. Specifically, increased proportions of resting DCs and eosinophils while decreased abundance of activated mast cells and monocytes were observed in early‐stage LUAD cases with low risk score. Notably, there were several contradictory findings concerning the effect of mast cells on the prognosis of LUAD. A report demonstrated that mast cells was associated with angiogenesis and unsatisfactory prognosis in stage I LUAD.^84^ Mast cells could facilitate growth and metastasis by generating IL‐1β in LUAD progression.^85^ Conversely, certain studies revealed that greater abundance of mast cells was related to better prognosis and prolonged survival in early‐stage LUAD cases.^54^, ^86^ We also found relatively enhanced expression of CD4 and CD8A indicative of a strengthened immune response in the low‐risk group.^7^ Conversely, patients with high risk score had increased levels of immunosuppressive molecules (such as PD‐L1, CTLA‐4 and TIGIT). Notably, in several reports on the anti‐PD‐1/PD‐L1 treatment for NSCLC, PD‐L1 expression in tumors has been considered as the criteria and a predictive biomarker for inferior prognosis. Interestingly, PD‐L1‐expressing NSCLC individuals have a higher possibility to obtain clinical benefit from immunotherapy.^15^, ^87^ Based on the aforementioned results, dysregulation of immune contexture is a potential reason for the OS differences between patient subgroups stratified by the IPM. The immunosuppressive microenvironment is one of the reasons why high‐risk subgroup was characterized with unsatisfactory clinical outcome.

In benchmark comparisons, our IPM achieved more satisfactory prognostic performance than a novel 21‐gene‐based immune‐related gene signature and an 8‐gene prognostic signature that have been published for early‐stage LUAD. Additionally, we conducted Cox analysis to identify that IPM risk score was an independent prognostic parameter. ROC curve with AUC values revealed that our IPM had more satisfactory predictive power than traditional prognostic variables. We further developed a novel nomogram model through leveraging the complementary value of the IPM and clinicopathologic characteristics (including age and TNM stage), further highlighting that combining both could confer an intuitionistic and accurate scoring system to estimate the OS of early‐stage LUAD in clinical practice.

Several limitations are deserved to be discussed in our research. Initially, our report was retrospective and these public databases were devoid of certain crucial clinicopathologic information (such as smoking and drinking status, family history, whether performed by surgical intervention and specific surgery types, whether receiving neoadjuvant chemoradiotherapy).^46^, ^48^ Thus, further multicenter clinical trials of larger sample size and more detailed clinical data are required to externally validate our findings. Furthermore, our IPM was constructed based on two immune genes. The molecular functions and biological effects of above genes are warranted to be investigated individually and conjunctively, further favoring their clinical utilization. Ultimately, our IPM risk score was estimated in accordance with the gene expression values. The intra‐tumor heterogeneity potentially resulted in sampling bias.

## 5 CONCLUSION

In conclusion, we established and validated a two immune gene‐based and TP53 status‐associated IPM through analyzing the TP53 mutation data, RNA expression, and clinical information of early‐stage LUADs in multiple independent datasets across different platforms. The IPM was considered as an independent prognostic biomarker for early‐stage LUAD. A nomogram model including the IPM and other clinicopathologic parameters was formulated to quantitatively predict the long‐term prognosis of early‐stage LUAD individuals, thus assisting physicians to make better clinical decisions.

## CONFLICT OF INTEREST

The authors have declared that no competing interest exists.

## AUTHOR CONTRIBUTIONS

Wei Lin and Chengde Wu designed/planned the study and wrote the paper. Chengde Wu acquired and analyzed data and performed computational modeling. Chengde Wu and Xiang Rao performed imaging analysis. Wei Lin, Chengde Wu, and Xiang Rao participated in discussion of related data.

## ETHICAL STATEMENT

The TCGA and GEO database is publicly available, and our study was performed based on the guideline of these databases. All patient information was anonymized and de‐identified in the TCGA and GEO database. Thus, our study was exempted from the ethics committee approval and patients' informed consent.

## Supporting information

Table S1

Table S2

Table S3

Table S4

Table S5

Table S6

## Data Availability

Publicly available datasets were analyzed in this study. These data can be found here: https://tcga‐data.nci.nih.gov/tcga/ and http://www.ncbi.nlm.nih.gov/geo/.

## References

[1] Rami‐Porta R , Asamura H , Travis WD , et al. Lung cancer ‐ major changes in the American Joint Committee on Cancer eighth edition cancer staging manual. CA Cancer J Clin. 2017;67(2):138‐155. 28140453 10.3322/caac.21390

[2] Mcgranahan N , Rosenthal R , Hiley CT , et al. Allele‐specific HLA loss and immune escape in lung cancer evolution. Cell. 2017;171(6):1259‐1271.e11. 29107330 10.1016/j.cell.2017.10.001 PMC5720478

[3] Zhou B , Flodby P , Luo J , et al. Claudin‐18‐mediated YAP activity regulates lung stem and progenitor cell homeostasis and tumorigenesis. J Clin Invest. 2018;128(3):970‐984. 29400695 10.1172/JCI90429 PMC5824875

[4] Zhang Z , Tang H , Chen P , et al. Demystifying the manipulation of host immunity, metabolism, and extraintestinal tumors by the gut microbiome. Signal Transduct Target Ther. 2019;4:41. 31637019 10.1038/s41392-019-0074-5 PMC6799818

[5] Siegel RL , Miller KD , Jemal A . Cancer statistics, 2020. CA Cancer J Clin. 2020;70(1):7‐30. 31912902 10.3322/caac.21590

[6] Doll KM , Rademaker A , Sosa JA . Practical guide to surgical data sets: surveillance, epidemiology, and end results (SEER) database. JAMA Surg. 2018;153(6):588‐589. 29617544 10.1001/jamasurg.2018.0501

[7] Kadara H , Choi M , Zhang J , et al. Whole‐exome sequencing and immune profiling of early‐stage lung adenocarcinoma with fully annotated clinical follow‐up. Ann Oncol. 2017;28(1):75‐82. 27687306 10.1093/annonc/mdw436 PMC5982809

[8] Zhang Z , Chen P , Xie H , et al. Using circulating tumor DNA as a novel biomarker to screen and diagnose hepatocellular carcinoma: A systematic review and meta‐analysis. Cancer Med. 2020;9(4):1349‐1364. 31876977 10.1002/cam4.2799 PMC7013058

[9] Peters S , Gettinger S , Johnson ML , et al. Phase II trial of atezolizumab as first‐line or subsequent therapy for patients with programmed death‐ligand 1‐selected advanced non‐small‐cell lung cancer (BIRCH). J Clin Oncol. 2017;35(24):2781‐2789. 28609226 10.1200/JCO.2016.71.9476 PMC5562171

[10] Liu ZJ , Huang Y , Wei L , et al. Combination of LINE‐1 hypomethylation and RASSF1A promoter hypermethylation in serum DNA is a non‐invasion prognostic biomarker for early recurrence of hepatocellular carcinoma after curative resection. Neoplasma. 2017;64(5):795‐802. 28592132 10.4149/neo_2017_519

[11] Liu S , Lai W , Shi Y , et al. Annotation and cluster analysis of long noncoding RNA linked to male sex and estrogen in cancers. NPJ Precis Oncol. 2020;4:5. 32195358 10.1038/s41698-020-0110-5 PMC7054536

[12] Liu X , Wu S , Yang Y , et al. The prognostic landscape of tumor‐infiltrating immune cell and immunomodulators in lung cancer. Biomed Pharmacother. 2017;95:55‐61. 28826097 10.1016/j.biopha.2017.08.003

[13] Bao X , Shi R , Zhao T , et al. Immune landscape and a novel immunotherapy‐related gene signature associated with clinical outcome in early‐stage lung adenocarcinoma. J Mol Med (Berl). 2020;98(6):805‐818. 32333046 10.1007/s00109-020-01908-9 PMC7297823

[14] Forde PM , Chaft JE , Smith KN , et al. Neoadjuvant PD‐1 blockade in resectable lung cancer. N Engl J Med. 2018;378(21):1976‐1986. 29658848 10.1056/NEJMoa1716078 PMC6223617

[15] Sui H , Ma N , Wang Y , et al. Anti‐PD‐1/PD‐L1 therapy for non‐small‐cell lung cancer: toward personalized medicine and combination strategies. J Immunol Res. 2018;2018:6984948. 30159341 10.1155/2018/6984948 PMC6109480

[16] Rizvi NA , Hellmann MD , Snyder A , et al. Cancer immunology. Mutational landscape determines sensitivity to PD‐1 blockade in non‐small cell lung cancer. Science. 2015;348(6230):124‐128. 25765070 10.1126/science.aaa1348 PMC4993154

[17] Le DT , Durham JN , Smith KN , et al. Mismatch repair deficiency predicts response of solid tumors to PD‐1 blockade. Science. 2017;357(6349):409‐413. 28596308 10.1126/science.aan6733 PMC5576142

[18] Hellmann MD , Ciuleanu TE , Pluzanski A , et al. Nivolumab plus ipilimumab in lung cancer with a high tumor mutational burden. N Engl J Med. 2018;378(22):2093‐2104. 29658845 10.1056/NEJMoa1801946 PMC7193684

[19] Gridelli C , Casaluce F . Frontline immunotherapy for NSCLC: alone or not alone? Nat Rev Clin Oncol. 2018;15(10):593‐594. 29993034 10.1038/s41571-018-0070-7

[20] Mok TSK , Wu YL , Kudaba I , et al. Pembrolizumab versus chemotherapy for previously untreated, PD‐L1‐expressing, locally advanced or metastatic non‐small‐cell lung cancer (KEYNOTE‐042): a randomised, open‐label, controlled, phase 3 trial. Lancet. 2019;393(10183):1819‐1830. 30955977 10.1016/S0140-6736(18)32409-7

[21] Schalper KA , Brown J , Carvajal‐Hausdorf D , et al. Objective measurement and clinical significance of TILs in non‐small cell lung cancer. J Natl Cancer Inst. 2015;107(3):435 10.1093/jnci/dju435 PMC4565530 25650315

[22] Zhao Z , Zhao D , Xia J , et al. Immunoscore predicts survival in early‐stage lung adenocarcinoma patients. Front Oncol. 2020;10:691. 32457841 10.3389/fonc.2020.00691 PMC7225293

[23] Kastenhuber ER , Lowe SW . Putting p53 in context. Cell. 2017;170(6):1062‐1078. 28886379 10.1016/j.cell.2017.08.028 PMC5743327

[24] Mantovani F , Collavin L , Del Sal G . Mutant p53 as a guardian of the cancer cell. Cell Death Differ. 2019;26(2):199‐212. 30538286 10.1038/s41418-018-0246-9 PMC6329812

[25] Sabapathy K , Lane DP . Therapeutic targeting of p53: all mutants are equal, but some mutants are more equal than others. Nat Rev Clin Oncol. 2018;15(1):13‐30. 28948977 10.1038/nrclinonc.2017.151

[26] Yue X , Zhao Y , Xu Y , et al. Mutant p53 in cancer: accumulation, gain‐of‐function, and therapy. J Mol Biol. 2017;429(11):1595‐1606. 28390900 10.1016/j.jmb.2017.03.030 PMC5663274

[27] Terra SB , Jang JS , Bi L , et al. Molecular characterization of pulmonary sarcomatoid carcinoma: analysis of 33 cases. Mod Pathol. 2016;29(8):824‐831. 27174587 10.1038/modpathol.2016.89

[28] Coudray N , Ocampo PS , Sakellaropoulos T , et al. Classification and mutation prediction from non‐small cell lung cancer histopathology images using deep learning. Nat Med. 2018;24(10):1559‐1567. 30224757 10.1038/s41591-018-0177-5 PMC9847512

[29] Zhao ZR , Lin YB , Ng CSH , et al. Mutation profile of resected EGFR‐mutated lung adenocarcinoma by next‐generation sequencing. Oncologist. 2019;24(10):1368‐1374. 30872465 10.1634/theoncologist.2018-0567 PMC6795151

[30] Li L , Li M , Wang X . Cancer type‐dependent correlations between TP53 mutations and antitumor immunity. DNA Repair (Amst). 2020;88:102785. 32007736 10.1016/j.dnarep.2020.102785

[31] Chen H , Carrot‐Zhang J , Zhao Y , et al. Genomic and immune profiling of pre‐invasive lung adenocarcinoma. Nat Commun. 2019;10(1):5472. 31784532 10.1038/s41467-019-13460-3 PMC6884501

[32] Chen Y , Chen G , Li J , et al. Association of tumor protein p53 and ataxia‐telangiectasia mutated comutation with response to immune checkpoint inhibitors and mortality in patients with non‐small cell lung cancer. JAMA Netw Open. 2019;2(9):e1911895. 31539077 10.1001/jamanetworkopen.2019.11895 PMC6755545

[33] Dong ZY , Zhang JT , Liu SY , et al. EGFR mutation correlates with uninflamed phenotype and weak immunogenicity, causing impaired response to PD‐1 blockade in non‐small cell lung cancer. Oncoimmunology. 2017;6(11):e1356145. 29147605 10.1080/2162402X.2017.1356145 PMC5674946

[34] Dong ZY , Zhong WZ , Zhang XC , et al. Potential predictive value of TP53 and KRAS mutation status for response to PD‐1 blockade immunotherapy in lung adenocarcinoma. Clin Cancer Res. 2017;23(12):3012‐3024. 28039262 10.1158/1078-0432.CCR-16-2554

[35] Long J , Wang A , Bai Y , et al. Development and validation of a TP53‐associated immune prognostic model for hepatocellular carcinoma. EBioMedicine. 2019;42:363‐374. 30885723 10.1016/j.ebiom.2019.03.022 PMC6491941

[36] Yu G , Wang LG , Han Y , et al. clusterProfiler: an R package for comparing biological themes among gene clusters. Omics. 2012;16(5):284‐287. 22455463 10.1089/omi.2011.0118 PMC3339379

[37] Robinson MD , Mccarthy DJ , Smyth GK . edgeR: a bioconductor package for differential expression analysis of digital gene expression data. Bioinformatics. 2010;26(1):139‐140. 19910308 10.1093/bioinformatics/btp616 PMC2796818

[38] Davis S , Meltzer PS . GEOquery: a bridge between the gene expression omnibus (GEO) and BioConductor. Bioinformatics. 2007;23(14):1846‐1847. 17496320 10.1093/bioinformatics/btm254

[39] Reimand J , Isserlin R , Voisin V , et al. Pathway enrichment analysis and visualization of omics data using g: Profiler, GSEA, Cytoscape and EnrichmentMap. Nat Protoc. 2019;14(2):482‐517. 30664679 10.1038/s41596-018-0103-9 PMC6607905

[40] Ritchie ME , Phipson B , Wu D , et al. limma powers differential expression analyses for RNA‐sequencing and microarray studies. Nucleic Acids Res. 2015;43(7):e47. 25605792 10.1093/nar/gkv007 PMC4402510

[41] Rizvi AA , Karaesmen E , Morgan M , et al. gwasurvivr: an R package for genome‐wide survival analysis. Bioinformatics. 2019;35(11):1968‐1970. 30395168 10.1093/bioinformatics/bty920 PMC7963072

[42] Wong WW , Griesman J , Feng ZZ . Imputing genotypes using regularized generalized linear regression models. Stat Appl Genet Mol Biol. 2014;13(5):519‐529. 25029086 10.1515/sagmb-2012-0044

[43] Camp RL , Dolled‐Filhart M , Rimm DL . X‐tile: a new bio‐informatics tool for biomarker assessment and outcome‐based cut‐point optimization. Clin Cancer Res. 2004;10(21):7252‐7259. 15534099 10.1158/1078-0432.CCR-04-0713

[44] Heagerty PJ , Zheng Y . Survival model predictive accuracy and ROC curves. Biometrics. 2005;61(1):92‐105. 15737082 10.1111/j.0006-341X.2005.030814.x

[45] Chen B , Khodadoust MS , Liu CL , et al. Profiling tumor infiltrating immune cells with CIBERSORT. Methods Mol Biol. 2018;1711:243‐259. 29344893 10.1007/978-1-4939-7493-1_12 PMC5895181

[46] Zhang Z , Xie H , Chen P , et al. Development and identification of a nomogram prognostic model for patients with primary clear cell carcinoma of the liver. Med Sci Monit. 2020;26:e919789. 31969554 10.12659/MSM.919789 PMC6996864

[47] Iasonos A , Schrag D , Raj GV , et al. How to build and interpret a nomogram for cancer prognosis. J Clin Oncol. 2008;26(8):1364‐1370. 18323559 10.1200/JCO.2007.12.9791

[48] Zhang Y , Xie H , Zhang Z , et al. The characteristics and nomogram for primary lung papillary adenocarcinoma. Open Med (Wars). 2020;15:92‐102. 32195357 10.1515/med-2020-0014 PMC7070103

[49] Chen P , Zhang Z , Chen X . Overexpression of PKMYT1 facilitates tumor development and is correlated with poor prognosis in clear cell renal cell carcinoma. Med Sci Monit. 2020;26:e926755. 33024069 10.12659/MSM.926755 PMC7549326

[50] Shen S , Wang G , Zhang R , et al. Development and validation of an immune gene‐set based prognostic signature in ovarian cancer. EBioMedicine. 2019;40:318‐326. 30594555 10.1016/j.ebiom.2018.12.054 PMC6412087

[51] He R , Zuo S . A robust 8‐gene prognostic signature for early‐stage non‐small cell lung cancer. Front Oncol. 2019;9:693. 31417870 10.3389/fonc.2019.00693 PMC6684755

[52] Wu P , Zheng Y , Wang Y , et al. Development and validation of a robust immune‐related prognostic signature in early‐stage lung adenocarcinoma. J Transl Med. 2020;18(1):380. 33028329 10.1186/s12967-020-02545-z PMC7542703

[53] Chaudhary K , Poirion OB , Lu L , et al. Deep learning‐based multi‐omics integration robustly predicts survival in liver cancer. Clin Cancer Res. 2018;24(6):1248‐1259. 28982688 10.1158/1078-0432.CCR-17-0853 PMC6050171

[54] Bao X , Shi R , Zhao T , et al. Mast cell‐based molecular subtypes and signature associated with clinical outcome in early‐stage lung adenocarcinoma. Mol Oncol. 2020;14(5):917‐932. 32175651 10.1002/1878-0261.12670 PMC7191192

[55] Biton J , Mansuet‐Lupo A , Pécuchet N , et al. TP53, STK11, and EGFR mutations predict tumor immune profile and the response to anti‐PD‐1 in lung adenocarcinoma. Clin Cancer Res. 2018;24(22):5710‐5723. 29764856 10.1158/1078-0432.CCR-18-0163

[56] Thorsson V , Gibbs DL , Brown SD , et al. The immune landscape of cancer. Immunity. 2018;48(4):812‐830.e14. 29628290 10.1016/j.immuni.2018.03.023 PMC5982584

[57] Freudenstein D , Litchfield C , Caramia F , et al. TP53 status, patient sex, and the immune response as determinants of lung cancer patient survival. Cancers (Basel). 2020;12(6), 1535. 10.3390/cancers12061535 PMC7352604 32545367

[58] Xu JY , Zhang C , Wang X , et al. Integrative proteomic characterization of human lung adenocarcinoma. Cell. 2020;182(1):245‐261.e17. 32649877 10.1016/j.cell.2020.05.043

[59] Tilstam PV , Qi D , Leng L , et al. MIF family cytokines in cardiovascular diseases and prospects for precision‐based therapeutics. Expert Opin Ther Targets. 2017;21(7):671‐683. 28562118 10.1080/14728222.2017.1336227 PMC6130320

[60] Calandra T , Bernhagen J , Mitchell RA , et al. The macrophage is an important and previously unrecognized source of macrophage migration inhibitory factor. J Exp Med. 1994;179(6):1895‐1902. 8195715 10.1084/jem.179.6.1895 PMC2191507

[61] Bacher M , Metz CN , Calandra T , et al. An essential regulatory role for macrophage migration inhibitory factor in T‐cell activation. Proc Natl Acad Sci USA. 1996;93(15):7849‐7854. 8755565 10.1073/pnas.93.15.7849 PMC38837

[62] Rossi AG , Haslett C , Hirani N , et al. Human circulating eosinophils secrete macrophage migration inhibitory factor (MIF). Potential role in asthma. J Clin Invest. 1998;101(12):2869‐2874. 9637721 10.1172/JCI1524 PMC508878

[63] Bloom BR , Bennett B . Mechanism of a reaction in vitro associated with delayed‐type hypersensitivity. Science. 1966;153(3731):80‐82. 5938421 10.1126/science.153.3731.80

[64] David JR . Delayed hypersensitivity in vitro: its mediation by cell‐free substances formed by lymphoid cell‐antigen interaction. Proc Natl Acad Sci USA. 1966;56(1):72‐77. 5229858 10.1073/pnas.56.1.72 PMC285677

[65] Kang I , Bucala R . The immunobiology of MIF: function, genetics and prospects for precision medicine. Nat Rev Rheumatol. 2019;15(7):427‐437. 31197253 10.1038/s41584-019-0238-2

[66] Gordon‐Weeks AN , Lim SY , Yuzhalin AE , et al. Macrophage migration inhibitory factor: a key cytokine and therapeutic target in colon cancer. Cytokine Growth Factor Rev. 2015;26(4):451‐461. 25882738 10.1016/j.cytogfr.2015.03.002

[67] Penticuff JC , Woolbright BL , Sielecki TM , et al. MIF family proteins in genitourinary cancer: tumorigenic roles and therapeutic potential. Nat Rev Urol. 2019;16(5):318‐328. 30914802 10.1038/s41585-019-0171-9

[68] Oliveira CS , De Bock CE , Molloy TJ , et al. Macrophage migration inhibitory factor engages PI3K/Akt signalling and is a prognostic factor in metastatic melanoma. BMC Cancer. 2014;14:630. 25168062 10.1186/1471-2407-14-630 PMC4155090

[69] Dumitru CA , Gholaman H , Trellakis S , et al. Tumor‐derived macrophage migration inhibitory factor modulates the biology of head and neck cancer cells via neutrophil activation. Int J Cancer. 2011;129(4):859‐869. 21328346 10.1002/ijc.25991

[70] Alban TJ , Bayik D , Otvos B , et al. Glioblastoma myeloid‐derived suppressor cell subsets express differential macrophage migration inhibitory factor receptor profiles that can be targeted to reduce immune suppression. Front Immunol. 2020;11:1191. 32625208 10.3389/fimmu.2020.01191 PMC7315581

[71] Xu X , Wang B , Ye C , et al. Overexpression of macrophage migration inhibitory factor induces angiogenesis in human breast cancer. Cancer Lett. 2008;261(2):147‐157. 18171602 10.1016/j.canlet.2007.11.028

[72] Kamimura A , Kamachi M , Nishihira J , et al. Intracellular distribution of macrophage migration inhibitory factor predicts the prognosis of patients with adenocarcinoma of the lung. Cancer. 2000;89(2):334‐341. 10918163

[73] Tomiyasu M , Yoshino I , Suemitsu R , et al. Quantification of macrophage migration inhibitory factor mRNA expression in non‐small cell lung cancer tissues and its clinical significance. Clin Cancer Res. 2002;8(12):3755‐3760. 12473586

[74] Huang WC , Kuo KT , Wang CH , et al. Cisplatin resistant lung cancer cells promoted M2 polarization of tumor‐associated macrophages via the Src/CD155/MIF functional pathway. J Exp Clin Cancer Res. 2019;38(1):180. 31036057 10.1186/s13046-019-1166-3 PMC6489343

[75] Li J , Zhang J , Xie F , et al. Macrophage migration inhibitory factor promotes Warburg effect via activation of the NF‐κB/HIF‐1α pathway in lung cancer. Int J Mol Med. 2018;41(2):1062‐1068. 29207023 10.3892/ijmm.2017.3277

[76] Jäger B , Klatt D , Plappert L , et al. CXCR4/MIF axis amplifies tumor growth and epithelial‐mesenchymal interaction in non‐small cell lung cancer. Cell Signal. 2020;73:109672. 32428553 10.1016/j.cellsig.2020.109672

[77] Wei L , Huang Y , Zhao R , et al. Detection of promoter methylation status of suppressor of cytokine signaling 3 (SOCS3) in tissue and plasma from Chinese patients with different hepatic diseases. Clin Exp Med. 2018;18(1):79‐87. 28951980 10.1007/s10238-017-0473-2

[78] Lua I , Li Y , Zagory JA , et al. Characterization of hepatic stellate cells, portal fibroblasts, and mesothelial cells in normal and fibrotic livers. J Hepatol. 2016;64(5):1137‐1146. 26806818 10.1016/j.jhep.2016.01.010 PMC4834254

[79] Buffon A , Wink MR , Ribeiro BV , et al. NTPDase and 5’ ecto‐nucleotidase expression profiles and the pattern of extracellular ATP metabolism in the Walker 256 tumor. Biochim Biophys Acta. 2007;1770(8):1259‐1265. 17574764 10.1016/j.bbagen.2007.05.004

[80] Bertoni APS , De Campos RP , Tsao M , et al. Extracellular ATP is differentially metabolized on papillary thyroid carcinoma cells surface in comparison to normal cells. Cancer Microenviron. 2018;11(1):61‐70. 29455338 10.1007/s12307-018-0206-4 PMC6008267

[81] Braganhol E , Zanin RF , Bernardi A , et al. Overexpression of NTPDase2 in gliomas promotes systemic inflammation and pulmonary injury. Purinergic Signal. 2012;8(2):235‐243. 22038661 10.1007/s11302-011-9276-1 PMC3350588

[82] Chiu DK , Tse AP , Xu IM , et al. Hypoxia inducible factor HIF‐1 promotes myeloid‐derived suppressor cells accumulation through ENTPD2/CD39L1 in hepatocellular carcinoma. Nat Commun. 2017;8(1):517. 28894087 10.1038/s41467-017-00530-7 PMC5593860

[83] Santos Jr. AA , Cappellari AR , De Marchi FO , et al. Potential role of P2X7R in esophageal squamous cell carcinoma proliferation. Purinergic Signal. 2017;13(3):279‐292. 28397110 10.1007/s11302-017-9559-2 PMC5563289

[84] Baram D , Vaday GG , Salamon P , et al. Human mast cells release metalloproteinase‐9 on contact with activated T cells: juxtacrine regulation by TNF‐alpha. J Immunol. 2001;167(7):4008‐4016. 11564820 10.4049/jimmunol.167.7.4008

[85] Lilis I , Ntaliarda G , Papaleonidopoulos V , et al. Interleukin‐1β provided by KIT‐competent mast cells is required for KRAS‐mutant lung adenocarcinoma. Oncoimmunology. 2019;8(7):1593802. 31143511 10.1080/2162402X.2019.1593802 PMC6527299

[86] Kurebayashi Y , Emoto K , Hayashi Y , et al. Comprehensive immune profiling of lung adenocarcinomas reveals four immunosubtypes with plasma cell subtype a negative indicator. Cancer Immunol Res. 2016;4(3):234‐247. 26787825 10.1158/2326-6066.CIR-15-0214

[87] Guo D , Wang M , Shen Z , et al. A new immune signature for survival prediction and immune checkpoint molecules in lung adenocarcinoma. J Transl Med. 2020;18(1):123. 32143735 10.1186/s12967-020-02286-z PMC7060601

