# Supplementary material for: Immune landscape and a promising immune prognostic model associated with TP53 in early‐stage lung adenocarcinoma
Source: Cancer Med. 2020 Dec 12;10(3):806–23. doi: 10.1002/cam4.3655 (PMC7897963; doi:10.1002/cam4.3655)
Supplement: Supplementary file 1 — Table S1 [file CAM4-10-806-s001.docx]

**Supplementary table 1**: The GSEA results of TP53^WT^ early-stage LUAD patients.

| NAME | SIZE | ES | NES | NOM p-val |
| --- | --- | --- | --- | --- |
| GO_REGULATION_OF_PROTEIN_LOCALIZATION_TO_CELL_SURFACE | 27 | 0.593506 | 1.913115 | 0 |
| GO_EPITHELIAL_TUBE_BRANCHING_INVOLVED_IN_LUNG_MORPHOGENESIS | 25 | 0.680378 | 1.878511 | 0.001988072 |
| GO_REGULATION_OF_POTASSIUM_ION_TRANSMEMBRANE_TRANSPORTER_ACTIVITY | 40 | 0.622608 | 1.87693 | 0.001886793 |
| **GO_ORGAN_OR_TISSUE_SPECIFIC_**  **IMMUNE_RESPONSE** | **15** | **0.736463** | **1.835784** | **0** |
| GO_REGULATION_OF_PROTEIN_ACTIVATION_CASCADE | 34 | 0.697178 | 1.829223 | 0.010060363 |
| GO_LIPID_HOMEOSTASIS | 96 | 0.498747 | 1.818678 | 0 |
| GO_POSITIVE_REGULATION_OF_LIPID_STORAGE | 17 | 0.662762 | 1.774443 | 0.005703422 |
| GO_LUNG_MORPHOGENESIS | 45 | 0.545983 | 1.771871 | 0.007968128 |
| GO_POSITIVE_REGULATION_OF_POTASSIUM_ION_TRANSPORT | 36 | 0.608415 | 1.766106 | 0.007766991 |
| GO_PROTEIN_ACTIVATION_CASCADE | 65 | 0.62524 | 1.756885 | 0.00589391 |
| GO_BROWN_FAT_CELL_DIFFERENTIATION | 26 | 0.622491 | 1.755698 | 0.002016129 |
| GO_REGULATION_OF_SODIUM_ION_TRANSMEMBRANE_TRANSPORT | 46 | 0.540234 | 1.745482 | 0.00617284 |
| **GO_REGULATION_OF_HUMORAL_IMMUNE_RESPONSE** | **47** | **0.626143** | **1.744821** | **0.01004016** |
| GO_FATTY_ACID_BETA_OXIDATION_USING_ACYL_COA_DEHYDROGENASE | 18 | 0.668422 | 1.744464 | 0.006 |
| GO_PHOSPHATIDYLGLYCEROL_ACYL_CHAIN_REMODELING | 16 | 0.692119 | 1.744346 | 0.010060363 |
| GO_EXCRETION | 38 | 0.631789 | 1.743455 | 0.006085193 |
| **GO_RESPONSE_TO_INTERLEUKIN_6** | **25** | **0.588886** | **1.730536** | **0.006263048** |
| GO_STEROL_HOMEOSTASIS | 51 | 0.549258 | 1.729584 | 0.00996016 |
| GO_REGULATION_OF_SODIUM_ION_TRANSMEMBRANE_TRANSPORTER_ACTIVITY | 36 | 0.576508 | 1.729036 | 0.004132231 |
| GO_RENAL_WATER_HOMEOSTASIS | 30 | 0.590689 | 1.726942 | 0.003861004 |
| GO_PHOSPHATIDYLGLYCEROL_METABOLIC_PROCESS | 30 | 0.556329 | 1.725 | 0.006 |
| GO_REGULATION_OF_POTASSIUM_ION_TRANSPORT | 78 | 0.54268 | 1.72215 | 0.007590133 |
| GO_FLUID_TRANSPORT | 23 | 0.660028 | 1.716062 | 0.001926782 |
| GO_PROTEIN_LOCALIZATION_TO_CELL_SURFACE | 21 | 0.658946 | 1.692954 | 0.017475728 |
| GO_REGULATION_OF_CALCIUM_ION_TRANSMEMBRANE_TRANSPORTER_ACTIVITY | 65 | 0.507422 | 1.692822 | 0.007782101 |
| GO_LIVER_REGENERATION | 19 | 0.571393 | 1.691268 | 0.009920635 |
| GO_SODIUM_ION_HOMEOSTASIS | 27 | 0.617109 | 1.681368 | 0.009940358 |
| GO_ECTODERMAL_PLACODE_DEVELOPMENT | 15 | 0.616995 | 1.676697 | 0.031558186 |
| GO_HYDROGEN_PEROXIDE_METABOLIC_PROCESS | 29 | 0.580263 | 1.674824 | 0.021825397 |
| GO_RIBONUCLEOTIDE_CATABOLIC_PROCESS | 28 | 0.57599 | 1.673474 | 0.017928287 |
| GO_RESPIRATORY_GASEOUS_EXCHANGE | 46 | 0.513393 | 1.671245 | 0.017307693 |
| GO_NEGATIVE_REGULATION_OF_HEART_CONTRACTION | 18 | 0.65641 | 1.671046 | 0.015748031 |
| GO_PHOSPHATIDIC_ACID_METABOLIC_PROCESS | 30 | 0.513842 | 1.670592 | 0.009746589 |
| GO_SMOOTH_MUSCLE_CELL_DIFFERENTIATION | 28 | 0.605591 | 1.669728 | 0.020637898 |
| GO_REGULATION_OF_STRIATED_MUSCLE_CELL_APOPTOTIC_PROCESS | 20 | 0.57344 | 1.664082 | 0.014705882 |
| GO_ALDITOL_PHOSPHATE_METABOLIC_PROCESS | 32 | 0.540348 | 1.662568 | 0.011976048 |
| GO_REACTIVE_OXYGEN_SPECIES_METABOLIC_PROCESS | 87 | 0.467249 | 1.662554 | 0.006 |
| GO_ORGANIC_HYDROXY_COMPOUND_TRANSPORT | 141 | 0.461664 | 1.660002 | 0.002008032 |
| GO_NEGATIVE_REGULATION_OF_POTASSIUM_ION_TRANSMEMBRANE_TRANSPORTER_ACTIVITY | 15 | 0.679679 | 1.659634 | 0.0078125 |
| GO_BICARBONATE_TRANSPORT | 43 | 0.580896 | 1.659522 | 0.011764706 |
| GO_REGULATION_OF_CARDIAC_MUSCLE_CONTRACTION_BY_CALCIUM_ION_SIGNALING | 23 | 0.582311 | 1.65859 | 0.033203125 |
| GO_REGULATION_OF_PROTEIN_EXIT_FROM_ENDOPLASMIC_RETICULUM | 18 | 0.494802 | 1.65801 | 0.04206501 |
| GO_REGULATION_OF_POTASSIUM_ION_TRANSMEMBRANE_TRANSPORT | 58 | 0.531099 | 1.651334 | 0.009541985 |
| GO_RENAL_SYSTEM_PROCESS | 92 | 0.508664 | 1.646619 | 0.01183432 |
| GO_DRUG_METABOLIC_PROCESS | 29 | 0.637363 | 1.646053 | 0.013487476 |
| GO_POSITIVE_REGULATION_OF_LIPID_BIOSYNTHETIC_PROCESS | 56 | 0.479172 | 1.642272 | 0.008196721 |
| **GO_CELLULAR_RESPONSE_TO_**  **INTERLEUKIN_6** | **21** | **0.556957** | **1.641384** | **0.016563147** |
| GO_HYDROGEN_PEROXIDE_CATABOLIC_PROCESS | 19 | 0.598439 | 1.640816 | 0.021611001 |
| **GO_COMPLEMENT_ACTIVATION** | **44** | **0.624068** | **1.632928** | **0.024439918** |
| GO_NEGATIVE_REGULATION_OF_POTASSIUM_ION_TRANSPORT | 29 | 0.600676 | 1.627447 | 0.013257576 |
| GO_ACTIVATION_OF_PROTEIN_KINASE_B_ACTIVITY | 20 | 0.573142 | 1.627273 | 0.025793651 |
| GO_REGULATION_OF_INSULIN_SECRETION_INVOLVED_IN_CELLULAR_RESPONSE_TO_GLUCOSE_STIMULUS | 46 | 0.477501 | 1.62398 | 0.017928287 |
| GO_REGULATION_OF_TRANSPORTER_ACTIVITY | 178 | 0.43662 | 1.62236 | 0.007936508 |
| GO_NEURON_MATURATION | 30 | 0.532974 | 1.619924 | 0.007722008 |
| GO_PANCREAS_DEVELOPMENT | 61 | 0.50275 | 1.61746 | 0.01171875 |
| GO_BLOOD_COAGULATION_FIBRIN_CLOT_FORMATION | 23 | 0.62739 | 1.615859 | 0.028056113 |
| GO_RENAL_SYSTEM_PROCESS_INVOLVED_IN_REGULATION_OF_SYSTEMIC_ARTERIAL_BLOOD_PRESSURE | 21 | 0.618279 | 1.61325 | 0.017857144 |
| GO_REGULATION_OF_CHOLESTEROL_EFFLUX | 19 | 0.565193 | 1.612816 | 0.026104419 |
| GO_NEGATIVE_REGULATION_OF_LIPID_TRANSPORT | 23 | 0.52071 | 1.611922 | 0.030120483 |
| GO_REGULATION_OF_SODIUM_ION_TRANSPORT | 74 | 0.453061 | 1.611114 | 0.013944224 |
| GO_SINGLE_ORGANISM_MEMBRANE_BUDDING | 71 | 0.342818 | 1.606428 | 0.037848607 |
| GO_TEMPERATURE_HOMEOSTASIS | 22 | 0.604153 | 1.606235 | 0.011976048 |
| GO_REGULATION_OF_CARDIAC_MUSCLE_CONTRACTION_BY_REGULATION_OF_THE_RELEASE_OF_SEQUESTERED_CALCIUM_ION | 19 | 0.609811 | 1.604147 | 0.03269231 |
| GO_NEGATIVE_REGULATION_OF_ORGAN_GROWTH | 21 | 0.530727 | 1.60316 | 0.042145595 |
| GO_TRACHEA_DEVELOPMENT | 20 | 0.600668 | 1.601074 | 0.023121387 |
| GO_SERTOLI_CELL_DIFFERENTIATION | 18 | 0.543194 | 1.595678 | 0.028409092 |
| GO_ETHANOLAMINE_CONTAINING_COMPOUND_METABOLIC_PROCESS | 80 | 0.458081 | 1.59482 | 0.01417004 |
| GO_PROTEIN_TARGETING_TO_PLASMA_MEMBRANE | 23 | 0.556119 | 1.59197 | 0.040076334 |
| GO_NEGATIVE_REGULATION_OF_PEPTIDE_SECRETION | 43 | 0.46724 | 1.589266 | 0.024 |
| GO_POSITIVE_REGULATION_OF_MACROPHAGE_DERIVED_FOAM_CELL_DIFFERENTIATION | 15 | 0.664232 | 1.587386 | 0.046692606 |
| GO_NEGATIVE_REGULATION_OF_POTASSIUM_ION_TRANSMEMBRANE_TRANSPORT | 19 | 0.618874 | 1.583795 | 0.01724138 |
| GO_MONOVALENT_INORGANIC_CATION_HOMEOSTASIS | 115 | 0.421598 | 1.580859 | 0.012024048 |
| GO_VASODILATION | 25 | 0.545105 | 1.579787 | 0.038854804 |
| GO_POSITIVE_REGULATION_OF_VASODILATION | 29 | 0.553064 | 1.576295 | 0.009505703 |
| GO_REGULATION_OF_LONG_TERM_SYNAPTIC_POTENTIATION | 19 | 0.586483 | 1.576267 | 0.040935673 |
| GO_POSITIVE_REGULATION_OF_SMALL_GTPASE_MEDIATED_SIGNAL_TRANSDUCTION | 36 | 0.522939 | 1.573038 | 0.026717557 |
| GO_SIALYLATION | 19 | 0.597223 | 1.571183 | 0.020120725 |
| **GO_REGULATION_OF_MACROPHAGE_**  **ACTIVATION** | **24** | **0.564455** | **1.569268** | **0.035196688** |
| GO_ADRENERGIC_RECEPTOR_SIGNALING_PATHWAY | 17 | 0.649543 | 1.567498 | 0.03088803 |
| GO_AMINE_METABOLIC_PROCESS | 121 | 0.420664 | 1.566711 | 0.021956088 |
| GO_RESPONSE_TO_MONOAMINE | 33 | 0.501711 | 1.565507 | 0.046332046 |
| GO_DIGESTION | 103 | 0.497655 | 1.564119 | 0.016359918 |
| GO_POSITIVE_REGULATION_OF_LIPID_TRANSPORT | 45 | 0.46913 | 1.562557 | 0.026209677 |
| GO_REGULATION_OF_MEMBRANE_REPOLARIZATION | 29 | 0.567168 | 1.562115 | 0.0256917 |
| GO_REGULATION_OF_RELEASE_OF_SEQUESTERED_CALCIUM_ION_INTO_CYTOSOL_BY_SARCOPLASMIC_RETICULUM | 24 | 0.571779 | 1.561506 | 0.043650795 |
| GO_SIGNAL_PEPTIDE_PROCESSING | 22 | 0.550009 | 1.55652 | 0.04819277 |
| GO_WATER_HOMEOSTASIS | 61 | 0.476805 | 1.556406 | 0.0251938 |
| GO_BLOOD_COAGULATION_INTRINSIC_PATHWAY | 16 | 0.634673 | 1.555581 | 0.04733728 |
| GO_POSITIVE_REGULATION_OF_LIPID_METABOLIC_PROCESS | 114 | 0.419915 | 1.554042 | 0.02173913 |
| GO_ORGANIC_HYDROXY_COMPOUND_METABOLIC_PROCESS | 444 | 0.364099 | 1.552261 | 0.014373717 |
| GO_REACTIVE_OXYGEN_SPECIES_BIOSYNTHETIC_PROCESS | 23 | 0.551278 | 1.550953 | 0.031746034 |
| GO_PRIMARY_ALCOHOL_METABOLIC_PROCESS | 44 | 0.511401 | 1.547675 | 0.032520324 |
| GO_REGULATION_OF_ORGANIC_ACID_TRANSPORT | 44 | 0.472205 | 1.54613 | 0.03206413 |
| GO_DRUG_TRANSMEMBRANE_TRANSPORT | 18 | 0.609547 | 1.545098 | 0.017892644 |
| GO_REGULATION_OF_HEAT_GENERATION | 15 | 0.635574 | 1.544736 | 0.037848607 |
| GO_BILE_ACID_AND_BILE_SALT_TRANSPORT | 27 | 0.516331 | 1.542969 | 0.034552846 |
| GO_PHOSPHATIDYLCHOLINE_METABOLIC_PROCESS | 59 | 0.468818 | 1.542639 | 0.020120725 |
| GO_CYCLIC_NUCLEOTIDE_CATABOLIC_PROCESS | 17 | 0.654159 | 1.538114 | 0.03508772 |
| GO_DIGESTIVE_SYSTEM_PROCESS | 54 | 0.51689 | 1.537798 | 0.034623217 |
| GO_RESPONSE_TO_THYROID_HORMONE | 21 | 0.482059 | 1.537751 | 0.042226486 |
| GO_ORGANOPHOSPHATE_CATABOLIC_PROCESS | 111 | 0.369937 | 1.537668 | 0.01443299 |
| GO_NEGATIVE_REGULATION_OF_MUSCLE_CONTRACTION | 20 | 0.603426 | 1.53712 | 0.033009708 |
| GO_REGULATION_OF_MACROPHAGE_DERIVED_FOAM_CELL_DIFFERENTIATION | 27 | 0.556281 | 1.536822 | 0.04743083 |
| GO_REGULATION_OF_SYSTEMIC_ARTERIAL_BLOOD_PRESSURE | 78 | 0.478168 | 1.536158 | 0.038910504 |
| GO_REGULATION_OF_CARDIAC_MUSCLE_CELL_MEMBRANE_REPOLARIZATION | 20 | 0.586362 | 1.535828 | 0.034351144 |
| GO_NEGATIVE_REGULATION_OF_CATECHOLAMINE_SECRETION | 15 | 0.670136 | 1.534937 | 0.03929273 |
| GO_RESPONSE_TO_CAMP | 99 | 0.418357 | 1.533374 | 0.030710172 |
| GO_RETINA_MORPHOGENESIS_IN_CAMERA_TYPE_EYE | 42 | 0.512666 | 1.532101 | 0.025641026 |
| GO_NEGATIVE_REGULATION_OF_MUSCLE_CELL_APOPTOTIC_PROCESS | 29 | 0.507481 | 1.53171 | 0.022727273 |
| GO_REGULATION_OF_FATTY_ACID_OXIDATION | 26 | 0.505587 | 1.526941 | 0.030364372 |
| GO_PHENOL_CONTAINING_COMPOUND_METABOLIC_PROCESS | 72 | 0.468799 | 1.523694 | 0.01814516 |
| GO_BONE_MINERALIZATION | 37 | 0.499343 | 1.523515 | 0.032388665 |
| **GO_ANTIMICROBIAL_HUMORAL_**  **RESPONSE** | **25** | **0.536277** | **1.522463** | **0.047034767** |
| GO_THYROID_HORMONE_METABOLIC_PROCESS | 15 | 0.649564 | 1.521236 | 0.04233871 |
| GO_WATER_TRANSPORT | 16 | 0.637216 | 1.520261 | 0.03550296 |
| GO_NEUROMUSCULAR_JUNCTION_DEVELOPMENT | 34 | 0.495848 | 1.519723 | 0.04789272 |
| GO_ECTODERM_DEVELOPMENT | 20 | 0.502406 | 1.519347 | 0.046184737 |
| GO_REGULATION_OF_DELAYED_RECTIFIER_POTASSIUM_CHANNEL_ACTIVITY | 17 | 0.606417 | 1.518602 | 0.023166023 |
| GO_LIPID_LOCALIZATION | 241 | 0.371287 | 1.517516 | 0.015384615 |
| GO_NEGATIVE_REGULATION_OF_GTPASE_ACTIVITY | 41 | 0.406045 | 1.517334 | 0.03846154 |
| GO_NEGATIVE_REGULATION_OF_STRIATED_MUSCLE_CELL_APOPTOTIC_PROCESS | 16 | 0.560536 | 1.517019 | 0.04914934 |
| GO_REGULATION_OF_SENSORY_PERCEPTION | 30 | 0.526232 | 1.516692 | 0.040899795 |
| GO_REGULATION_OF_PEPTIDE_SECRETION | 181 | 0.383959 | 1.516026 | 0.021526419 |
| GO_POSITIVE_REGULATION_OF_FATTY_ACID_BIOSYNTHETIC_PROCESS | 15 | 0.578047 | 1.515883 | 0.044680852 |
| GO_REGULATION_OF_CATION_TRANSMEMBRANE_TRANSPORT | 193 | 0.405561 | 1.514746 | 0.027504912 |
| GO_REGULATION_OF_LIPID_TRANSPORT | 84 | 0.402291 | 1.514269 | 0.026584867 |
| GO_NEUTRAL_LIPID_METABOLIC_PROCESS | 72 | 0.440732 | 1.514004 | 0.048076924 |
| GO_POSITIVE_REGULATION_OF_POTASSIUM_ION_TRANSMEMBRANE_TRANSPORT | 25 | 0.538787 | 1.509739 | 0.047528517 |
| GO_REGULATION_OF_LIPID_METABOLIC_PROCESS | 246 | 0.354347 | 1.508611 | 0.023904383 |
| GO_POSITIVE_REGULATION_OF_SODIUM_ION_TRANSPORT | 32 | 0.487035 | 1.504548 | 0.03976143 |
| GO_CARDIAC_MUSCLE_CELL_ACTION_POTENTIAL | 35 | 0.561133 | 1.503942 | 0.036363635 |
| GO_PHOSPHATIDYLCHOLINE_BIOSYNTHETIC_PROCESS | 25 | 0.504198 | 1.503306 | 0.04489796 |
| GO_STEROID_METABOLIC_PROCESS | 208 | 0.377071 | 1.502526 | 0.027290449 |
| GO_ACTIVATION_OF_PHOSPHOLIPASE_C_ACTIVITY | 27 | 0.582861 | 1.501448 | 0.046875 |
| GO_REGULATION_OF_BLOOD_PRESSURE | 149 | 0.43354 | 1.494779 | 0.043052837 |
| GO_PHOSPHOLIPID_TRANSPORT | 51 | 0.458446 | 1.492734 | 0.038910504 |
| GO_REGULATION_OF_NEUROLOGICAL_SYSTEM_PROCESS | 53 | 0.452347 | 1.492332 | 0.035643563 |
| GO_POSITIVE_REGULATION_OF_HEART_RATE | 22 | 0.546266 | 1.491798 | 0.032818533 |
| GO_POLYOL_TRANSPORT | 15 | 0.625995 | 1.49052 | 0.03937008 |
| GO_POSITIVE_REGULATION_OF_FATTY_ACID_METABOLIC_PROCESS | 28 | 0.4927 | 1.490006 | 0.04375 |
| GO_REGULATION_OF_CELL_FATE_COMMITMENT | 24 | 0.546061 | 1.488566 | 0.03777336 |
| GO_ALCOHOL_METABOLIC_PROCESS | 323 | 0.348808 | 1.486706 | 0.028513238 |
| GO_FATTY_ACID_METABOLIC_PROCESS | 268 | 0.359227 | 1.485806 | 0.038617887 |
| GO_FATTY_ACID_DERIVATIVE_METABOLIC_PROCESS | 87 | 0.456962 | 1.485429 | 0.039447732 |
| GO_REGULATION_OF_CARDIAC_CONDUCTION | 65 | 0.468407 | 1.484289 | 0.042718448 |
| GO_PURINE_CONTAINING_COMPOUND_CATABOLIC_PROCESS | 48 | 0.423991 | 1.483984 | 0.03869654 |
| GO_ARACHIDONIC_ACID_METABOLIC_PROCESS | 47 | 0.490033 | 1.482968 | 0.041501977 |
| GO_SECOND_MESSENGER_MEDIATED_SIGNALING | 144 | 0.436509 | 1.476087 | 0.037623763 |
| GO_REGULATION_OF_PEPTIDE_TRANSPORT | 225 | 0.362586 | 1.470459 | 0.017475728 |
| GO_NEGATIVE_REGULATION_OF_TRANSMEMBRANE_TRANSPORT | 77 | 0.375223 | 1.469439 | 0.031067962 |
| GO_NUCLEOSIDE_PHOSPHATE_CATABOLIC_PROCESS | 68 | 0.380703 | 1.46544 | 0.026584867 |
| GO_REGULATION_OF_BLOOD_CIRCULATION | 271 | 0.420132 | 1.464954 | 0.036608864 |
| GO_STEROL_TRANSPORT | 45 | 0.468029 | 1.464698 | 0.048543688 |
| GO_CELLULAR_MONOVALENT_INORGANIC_CATION_HOMEOSTASIS | 91 | 0.376537 | 1.463538 | 0.027777778 |
| GO_GLYCEROLIPID_BIOSYNTHETIC_PROCESS | 194 | 0.321746 | 1.458641 | 0.030549899 |
| GO_GLYCOSPHINGOLIPID_BIOSYNTHETIC_PROCESS | 22 | 0.511372 | 1.454472 | 0.039138943 |
| GO_CATECHOLAMINE_METABOLIC_PROCESS | 38 | 0.48296 | 1.447509 | 0.041322313 |
| GO_REGULATION_OF_CATION_CHANNEL_ACTIVITY | 81 | 0.41698 | 1.445977 | 0.046 |
| GO_NEGATIVE_REGULATION_OF_ION_TRANSPORT | 115 | 0.400582 | 1.437384 | 0.044401545 |
| GO_REGULATION_OF_HORMONE_LEVELS | 422 | 0.351744 | 1.424152 | 0.034343433 |
| GO_REGULATION_OF_HORMONE_SECRETION | 229 | 0.359889 | 1.420492 | 0.04040404 |
| GO_AMMONIUM_ION_METABOLIC_PROCESS | 157 | 0.362927 | 1.417379 | 0.033826638 |
| GO_OLIGODENDROCYTE_DIFFERENTIATION | 55 | 0.406634 | 1.407737 | 0.046875 |
| GO_REGULATION_OF_ANION_TRANSPORT | 124 | 0.357972 | 1.384185 | 0.044 |
| GO_ORGANIC_ANION_TRANSPORT | 341 | 0.328121 | 1.367015 | 0.031007752 |
| GO_ANION_TRANSPORT | 443 | 0.314321 | 1.339307 | 0.040697675 |
