# Supplementary material for: Immune landscape and a promising immune prognostic model associated with TP53 in early‐stage lung adenocarcinoma
Source: Cancer Med. 2020 Dec 12;10(3):806–23. doi: 10.1002/cam4.3655 (PMC7897963; doi:10.1002/cam4.3655)
Supplement: Supplementary file 2 — Table S2 [file CAM4-10-806-s002.docx]

**Supplementary table 2**: The GSEA results of TP53^MUT^ early-stage LUAD patients.

| NAME | SIZE | ES | NES | NOM p-val |
| --- | --- | --- | --- | --- |
| GO_REGULATION_OF_MICROTUBULE_POLYMERIZATION_OR_DEPOLYMERIZATION | 160 | -0.6345469 | -2.3398118 | 0 |
| GO_REGULATION_OF_MICROTUBULE_BASED_PROCESS | 221 | -0.5987523 | -2.297928 | 0 |
| **GO_REGULATION_OF_NUCLEAR_**  **DIVISION** | **149** | **-0.63383013** | **-2.2616107** | **0** |
| GO_REGULATION_OF_PROTEIN_COMPLEX_DISASSEMBLY | 193 | -0.5487575 | -2.2569644 | 0 |
| **GO_POSITIVE_REGULATION_OF_CELL_**  **CYCLE_PHASE_TRANSITION** | **65** | **-0.6662682** | **-2.233577** | **0** |
| GO_NEGATIVE_REGULATION_OF_CYTOSKELETON_ORGANIZATION | 199 | -0.5196312 | -2.2297618 | 0 |
| GO_NEGATIVE_REGULATION_OF_PROTEIN_COMPLEX_DISASSEMBLY | 151 | -0.57673883 | -2.220708 | 0 |
| **GO_CHROMOSOME_SEGREGATION** | **239** | **-0.74756354** | **-2.2201061** | **0** |
| GO_REGULATION_OF_SISTER_CHROMATID_SEGREGATION | 59 | -0.7454961 | -2.2162108 | 0 |
| GO_CELL_DIVISION | 414 | -0.61608356 | -2.2148895 | 0 |
| **GO_MITOTIC_NUCLEAR_DIVISION** | **331** | **-0.6809723** | **-2.2098193** | **0** |
| GO_REGULATION_OF_CYTOKINESIS | 52 | -0.6645198 | -2.205025 | 0 |
| GO_REGULATION_OF_CELL_DIVISION | 237 | -0.5569278 | -2.1893318 | 0 |
| GO_POSITIVE_REGULATION_OF_MITOTIC_CELL_CYCLE | 115 | -0.61720175 | -2.1844716 | 0 |
| GO_REGULATION_OF_CHROMOSOME_SEGREGATION | 75 | -0.75623363 | -2.180731 | 0 |
| GO_ORGANELLE_FISSION | 439 | -0.64030784 | -2.1777399 | 0 |
| GO_NEGATIVE_REGULATION_OF_CELL_DIVISION | 54 | -0.715157 | -2.177722 | 0 |
| GO_NUCLEAR_CHROMOSOME_SEGREGATION | 198 | -0.7501908 | -2.170517 | 0 |
| GO_NEGATIVE_REGULATION_OF_ORGANELLE_ORGANIZATION | 353 | -0.4643652 | -2.1688397 | 0 |
| GO_CYTOKINESIS | 79 | -0.60315335 | -2.1542103 | 0 |
| GO_SPINDLE_ASSEMBLY | 65 | -0.6633083 | -2.1267571 | 0 |
| GO_MITOTIC_SPINDLE_ORGANIZATION | 62 | -0.7629524 | -2.1256685 | 0 |
| GO_CHROMATIN_ASSEMBLY_OR_DISASSEMBLY | 93 | -0.6386911 | -2.1229305 | 0 |
| GO_POSITIVE_REGULATION_OF_CELL_CYCLE_PROCESS | 219 | -0.54368526 | -2.121913 | 0 |
| GO_REGULATION_OF_SPINDLE_ORGANIZATION | 19 | -0.7502795 | -2.119689 | 0 |
| GO_SISTER_CHROMATID_SEGREGATION | 159 | -0.77653843 | -2.1184196 | 0 |
| GO_DOUBLE_STRAND_BREAK_REPAIR | 129 | -0.65639985 | -2.1172602 | 0 |
| GO_NEGATIVE_REGULATION_OF_CHROMOSOME_ORGANIZATION | 91 | -0.5710656 | -2.1162045 | 0 |
| GO_DNA_PACKAGING | 104 | -0.69986475 | -2.1144319 | 0 |
| GO_MITOTIC_SISTER_CHROMATID_SEGREGATION | 85 | -0.8001563 | -2.1118622 | 0 |
| GO_NEGATIVE_REGULATION_OF_NUCLEAR_DIVISION | 41 | -0.70755845 | -2.1056747 | 0 |
| GO_NEGATIVE_REGULATION_OF_MITOTIC_NUCLEAR_DIVISION | 31 | -0.7569862 | -2.101556 | 0 |
| GO_DNA_RECOMBINATION | 181 | -0.64851123 | -2.1004717 | 0 |
| GO_DNA_CONFORMATION_CHANGE | 178 | -0.65421766 | -2.1003413 | 0 |
| **GO_DNA_REPAIR** | **411** | **-0.5501519** | **-2.100261** | **0** |
| GO_CHROMOSOME_LOCALIZATION | 56 | -0.7205784 | -2.0998795 | 0 |
| GO_MITOTIC_SPINDLE_ASSEMBLY | 37 | -0.7499715 | -2.0970647 | 0 |
| GO_REGULATION_OF_MITOTIC_CELL_CYCLE | 439 | -0.4716544 | -2.0968835 | 0 |
| GO_MICROTUBULE_CYTOSKELETON_ORGANIZATION | 307 | -0.523364 | -2.0930917 | 0.001876173 |
| GO_TELOMERE_ORGANIZATION | 73 | -0.6682684 | -2.0864165 | 0 |
| GO_DNA_REPLICATION | 190 | -0.68731034 | -2.0788019 | 0 |
| GO_CELL_CYCLE_CHECKPOINT | 175 | -0.60640776 | -2.0765092 | 0 |
| GO_PROTEIN_DNA_COMPLEX_SUBUNIT_ORGANIZATION | 146 | -0.5742757 | -2.0681047 | 0 |
| GO_RETROGRADE_VESICLE_MEDIATED_TRANSPORT_GOLGI_TO_ER | 74 | -0.5396102 | -2.0680578 | 0 |
| GO_CELL_CYCLE_PHASE_TRANSITION | 247 | -0.62334585 | -2.0637157 | 0 |
| GO_NEGATIVE_REGULATION_OF_CELLULAR_PROTEIN_CATABOLIC_PROCESS | 60 | -0.50542957 | -2.0556512 | 0 |
| GO_REGULATION_OF_SIGNAL_TRANSDUCTION_BY_P53_CLASS_MEDIATOR | 155 | -0.52150714 | -2.040929 | 0 |
| GO_MICROTUBULE_POLYMERIZATION_OR_DEPOLYMERIZATION | 37 | -0.60590047 | -2.0409112 | 0.001919386 |
| GO_MEIOTIC_CELL_CYCLE_PROCESS | 125 | -0.62852734 | -2.0324507 | 0 |
| GO_DNA_INTEGRITY_CHECKPOINT | 132 | -0.57015884 | -2.032266 | 0 |
| GO_POSITIVE_REGULATION_OF_CELL_CYCLE | 299 | -0.47352993 | -2.0316024 | 0 |
| GO_DNA_SYNTHESIS_INVOLVED_IN_DNA_REPAIR | 67 | -0.7055941 | -2.0296817 | 0 |
| GO_REGULATION_OF_CELL_CYCLE_PHASE_TRANSITION | 302 | -0.47481266 | -2.0279877 | 0 |
| GO_DNA_BIOSYNTHETIC_PROCESS | 104 | -0.660045 | -2.0260704 | 0 |
| GO_ATP_DEPENDENT_CHROMATIN_REMODELING | 53 | -0.7178217 | -2.0222096 | 0 |
| GO_NEGATIVE_REGULATION_OF_CELL_CYCLE_PROCESS | 193 | -0.50164914 | -2.0196788 | 0 |
| GO_MEIOTIC_CELL_CYCLE | 155 | -0.6105011 | -2.01848 | 0 |
| GO_NEGATIVE_REGULATION_OF_MITOTIC_CELL_CYCLE | 182 | -0.49335706 | -2.0077255 | 0 |
| GO_G2_DNA_DAMAGE_CHECKPOINT | 30 | -0.7550978 | -2.0066452 | 0 |
| GO_REGULATION_OF_CHROMOSOME_ORGANIZATION | 252 | -0.4357455 | -1.9998515 | 0 |
| GO_CHROMATIN_REMODELING | 126 | -0.5146786 | -1.9956504 | 0.001901141 |
| GO_REGULATION_OF_CELL_AGING | 32 | -0.6179728 | -1.995382 | 0 |
| GO_PROTEIN_DEPOLYMERIZATION | 22 | -0.7285957 | -1.9953499 | 0 |
| GO_NEGATIVE_REGULATION_OF_PROTEIN_MODIFICATION_BY_SMALL_PROTEIN_CONJUGATION_OR_REMOVAL | 133 | -0.5004574 | -1.9946238 | 0.005586592 |
| GO_MITOTIC_CELL_CYCLE_CHECKPOINT | 128 | -0.5599561 | -1.9931612 | 0 |
| GO_RECOMBINATIONAL_REPAIR | 65 | -0.7245881 | -1.9913108 | 0 |
| GO_CELL_CYCLE_G1_S_PHASE_TRANSITION | 107 | -0.7012154 | -1.985943 | 0.001984127 |
| GO_POSTREPLICATION_REPAIR | 48 | -0.6065808 | -1.9827083 | 0.001912046 |
| GO_NEGATIVE_REGULATION_OF_GENE_EXPRESSION_EPIGENETIC | 49 | -0.4943214 | -1.9813062 | 0.001855288 |
| GO_CYTOSKELETON_DEPENDENT_CYTOKINESIS | 37 | -0.68376166 | -1.9788711 | 0 |
| GO_METAPHASE_PLATE_CONGRESSION | 41 | -0.75139725 | -1.9782628 | 0 |
| GO_REGULATION_OF_PROTEASOMAL_UBIQUITIN_DEPENDENT_PROTEIN_CATABOLIC_PROCESS | 140 | -0.42274043 | -1.9771537 | 0 |
| GO_MICROTUBULE_ORGANIZING_CENTER_ORGANIZATION | 78 | -0.62415135 | -1.9746054 | 0 |
| GO_MICROTUBULE_BASED_PROCESS | 470 | -0.46101975 | -1.9723498 | 0.003766478 |
| GO_REGULATION_OF_CELLULAR_SENESCENCE | 26 | -0.6276112 | -1.9672676 | 0 |
| GO_NEGATIVE_REGULATION_OF_CHROMOSOME_SEGREGATION | 24 | -0.80107045 | -1.965852 | 0 |
| GO_DNA_GEOMETRIC_CHANGE | 76 | -0.64406025 | -1.9651738 | 0.002 |
| GO_DNA_DEPENDENT_DNA_REPLICATION | 89 | -0.754345 | -1.9637201 | 0 |
| GO_SPINDLE_LOCALIZATION | 36 | -0.6061068 | -1.9629294 | 0.00189394 |
| GO_SISTER_CHROMATID_COHESION | 97 | -0.76630783 | -1.9606259 | 0 |
| GO_MITOTIC_CYTOKINESIS | 30 | -0.71266866 | -1.9604082 | 0.001956947 |
| GO_POSITIVE_REGULATION_OF_MITOTIC_NUCLEAR_DIVISION | 44 | -0.64015216 | -1.9573437 | 0.001912046 |
| **GO_CELL_CYCLE_G2_M_PHASE_**  **TRANSITION** | **134** | **-0.5588566** | **-1.9541446** | **0** |
| GO_REGULATION_OF_CHROMATIN_SILENCING | 20 | -0.6949056 | -1.9541205 | 0 |
| GO_REGULATION_OF_CELL_CYCLE_G2_M_PHASE_TRANSITION | 54 | -0.60008407 | -1.9503516 | 0.001923077 |
| GO_NUCLEAR_ENVELOPE_ORGANIZATION | 74 | -0.48934042 | -1.9500363 | 0.009727626 |
| GO_MISMATCH_REPAIR | 28 | -0.6854289 | -1.9495639 | 0.004048583 |
| GO_REGULATION_OF_DNA_REPAIR | 65 | -0.5344432 | -1.9493235 | 0.001976285 |
| GO_INTERSTRAND_CROSS_LINK_REPAIR | 36 | -0.70997405 | -1.9461981 | 0.007797271 |
| GO_NUCLEUS_ORGANIZATION | 120 | -0.42450714 | -1.9441522 | 0.007766991 |
| GO_PROTEIN_SUMOYLATION | 111 | -0.5021781 | -1.9391584 | 0.005802708 |
| GO_RESPONSE_TO_IONIZING_RADIATION | 134 | -0.5006905 | -1.9381264 | 0.001937985 |
| GO_MITOTIC_G2_M_TRANSITION_CHECKPOINT | 16 | -0.7787992 | -1.9341712 | 0 |
| GO_CENTROSOME_CYCLE | 44 | -0.7042789 | -1.933479 | 0 |
| GO_NEGATIVE_REGULATION_OF_CELL_CYCLE_PHASE_TRANSITION | 133 | -0.48402402 | -1.9325347 | 0 |
| GO_SPINDLE_CHECKPOINT | 23 | -0.82740396 | -1.9258454 | 0 |
| GO_TELOMERE_MAINTENANCE_VIA_RECOMBINATION | 32 | -0.8142429 | -1.9256624 | 0.001886793 |
| GO_MEIOSIS_I | 70 | -0.6459178 | -1.9254913 | 0 |
| GO_MITOTIC_RECOMBINATION | 41 | -0.8066693 | -1.9208522 | 0.001901141 |
| GO_REGULATION_OF_CENTROSOME_CYCLE | 38 | -0.7004889 | -1.9207134 | 0.001960784 |
| GO_CENTROSOME_LOCALIZATION | 17 | -0.63706684 | -1.9186203 | 0.005836576 |
| GO_MEIOTIC_CHROMOSOME_SEGREGATION | 47 | -0.6764379 | -1.9145225 | 0 |
| GO_TRANSLESION_SYNTHESIS | 36 | -0.59773594 | -1.9110224 | 0.001949318 |
| GO_CHROMOSOME_ORGANIZATION_INVOLVED_IN_MEIOTIC_CELL_CYCLE | 39 | -0.66630137 | -1.9101247 | 0 |
| GO_PROTEIN_LOCALIZATION_TO_CHROMOSOME | 33 | -0.72115993 | -1.907171 | 0.001937985 |
| GO_HISTONE_PHOSPHORYLATION | 23 | -0.729887 | -1.9071591 | 0 |
| GO_NUCLEAR_EXPORT | 136 | -0.42198208 | -1.9062738 | 0.03307393 |
| GO_MAINTENANCE_OF_LOCATION_IN_CELL | 93 | -0.43366897 | -1.9040625 | 0.002057613 |
| GO_POSITIVE_REGULATION_OF_CYTOKINESIS | 25 | -0.7058599 | -1.9002038 | 0.005769231 |
| GO_CHROMOSOME_SEPARATION | 16 | -0.7784905 | -1.8930486 | 0 |
| GO_CHROMOSOME_CONDENSATION | 23 | -0.83007234 | -1.8918614 | 0 |
| GO_DNA_REPLICATION_INDEPENDENT_NUCLEOSOME_ORGANIZATION | 32 | -0.8287096 | -1.8895577 | 0 |
| GO_REGULATION_OF_UBIQUITIN_PROTEIN_LIGASE_ACTIVITY | 17 | -0.7262243 | -1.8891047 | 0.003710575 |
| GO_MITOTIC_DNA_INTEGRITY_CHECKPOINT | 92 | -0.5102208 | -1.8890376 | 0.003913894 |
| GO_MEMBRANE_DISASSEMBLY | 43 | -0.60485625 | -1.8890003 | 0.003898636 |
| GO_REGULATION_OF_DOUBLE_STRAND_BREAK_REPAIR | 32 | -0.6417347 | -1.8864878 | 0.001964637 |
| GO_ESTABLISHMENT_OF_SPINDLE_ORIENTATION | 24 | -0.58952075 | -1.8863508 | 0.009345794 |
| GO_RIBONUCLEOPROTEIN_COMPLEX_LOCALIZATION | 109 | -0.45912996 | -1.8824385 | 0.034351144 |
| GO_DNA_DAMAGE_RESPONSE_SIGNAL_TRANSDUCTION_RESULTING_IN_TRANSCRIPTION | 15 | -0.7094124 | -1.8810058 | 0 |
| GO_CENTROSOME_DUPLICATION | 31 | -0.6824746 | -1.8728768 | 0 |
| GO_POSITIVE_REGULATION_OF_CELL_CYCLE_G1_S_PHASE_TRANSITION | 28 | -0.58297664 | -1.8716084 | 0.005964215 |
| GO_REGULATION_OF_DNA_DEPENDENT_DNA_REPLICATION | 38 | -0.7190547 | -1.8693067 | 0.001996008 |
| GO_DNA_DAMAGE_RESPONSE_DETECTION_OF_DNA_DAMAGE | 36 | -0.6342117 | -1.8668976 | 0.001814882 |
| GO_NEGATIVE_REGULATION_OF_CELL_CYCLE_G2_M_PHASE_TRANSITION | 21 | -0.648346 | -1.8645927 | 0.001926782 |
| GO_HISTONE_EXCHANGE | 32 | -0.80051935 | -1.8607248 | 0 |
| GO_ESTABLISHMENT_OF_MITOTIC_SPINDLE_LOCALIZATION | 23 | -0.6149898 | -1.8599399 | 0.00742115 |
| GO_NUCLEOTIDE_EXCISION_REPAIR_DNA_GAP_FILLING | 24 | -0.6848643 | -1.8541296 | 0.003752345 |
| GO_HOMOLOGOUS_CHROMOSOME_SEGREGATION | 35 | -0.68169737 | -1.853224 | 0 |
| GO_MULTI_ORGANISM_LOCALIZATION | 64 | -0.47636542 | -1.8509356 | 0.01778656 |
| GO_REGULATION_OF_DNA_METABOLIC_PROCESS | 304 | -0.42840892 | -1.8506452 | 0 |
| GO_REGULATION_OF_RESPONSE_TO_DNA_DAMAGE_STIMULUS | 131 | -0.43294734 | -1.8442285 | 0.003913894 |
| GO_NEGATIVE_REGULATION_OF_CELL_CYCLE | 394 | -0.3890149 | -1.844197 | 0 |
| GO_ERROR_PRONE_TRANSLESION_SYNTHESIS | 19 | -0.71096206 | -1.8412932 | 0.00189394 |
| GO_RECIPROCAL_DNA_RECOMBINATION | 33 | -0.6591365 | -1.8394785 | 0.003968254 |
| GO_REGULATION_OF_PROTEASOMAL_PROTEIN_CATABOLIC_PROCESS | 172 | -0.36511862 | -1.8385404 | 0.001862197 |
| GO_NEGATIVE_REGULATION_OF_CELL_AGING | 16 | -0.6731208 | -1.8384173 | 0.001923077 |
| GO_REGULATION_OF_CYCLIN_DEPENDENT_PROTEIN_KINASE_ACTIVITY | 92 | -0.48599136 | -1.8370937 | 0 |
| GO_NON_RECOMBINATIONAL_REPAIR | 46 | -0.5405135 | -1.8282835 | 0.024809161 |
| GO_BLASTOCYST_DEVELOPMENT | 58 | -0.48490608 | -1.822673 | 0.003809524 |
| GO_REGULATION_OF_PROTEIN_MODIFICATION_BY_SMALL_PROTEIN_CONJUGATION_OR_REMOVAL | 264 | -0.37464973 | -1.8217161 | 0.011342155 |
| GO_REGULATION_OF_CYTOSKELETON_ORGANIZATION | 458 | -0.38477692 | -1.820317 | 0.002061856 |
| GO_PORE_COMPLEX_ASSEMBLY | 15 | -0.57877064 | -1.8184838 | 0.017928287 |
| GO_REGULATION_OF_DNA_REPLICATION | 148 | -0.46575025 | -1.8171095 | 0.004032258 |
| GO_DNA_REPLICATION_INITIATION | 29 | -0.8538434 | -1.8170968 | 0 |
| GO_POSITIVE_REGULATION_OF_NUCLEAR_DIVISION | 54 | -0.56457925 | -1.8167558 | 0.003913894 |
| GO_BLASTOCYST_GROWTH | 15 | -0.74666536 | -1.8157566 | 0.003976143 |
| GO_HISTONE_MRNA_METABOLIC_PROCESS | 24 | -0.6208587 | -1.8119972 | 0.0056926 |
| GO_REGULATION_OF_GENE_SILENCING | 39 | -0.51057965 | -1.8105297 | 0.003831418 |
| GO_CENTROMERE_COMPLEX_ASSEMBLY | 29 | -0.8685366 | -1.7995017 | 0 |
| GO_POSITIVE_REGULATION_OF_CELL_CYCLE_ARREST | 79 | -0.47000945 | -1.7975377 | 0.003891051 |
| GO_POSITIVE_REGULATION_OF_GENE_EXPRESSION_EPIGENETIC | 47 | -0.49592564 | -1.7931036 | 0.013565891 |
| GO_REGULATION_OF_GENE_EXPRESSION_EPIGENETIC | 156 | -0.37942404 | -1.7891709 | 0.026022306 |
| GO_SIGNAL_TRANSDUCTION_IN_RESPONSE_TO_DNA_DAMAGE | 91 | -0.46449655 | -1.7879503 | 0.001890359 |
| GO_POSITIVE_REGULATION_OF_CHROMOSOME_SEGREGATION | 22 | -0.7244163 | -1.7879359 | 0.009578544 |
| GO_BASE_EXCISION_REPAIR | 39 | -0.58490795 | -1.786321 | 0.009416196 |
| GO_NEGATIVE_REGULATION_OF_CHROMATIN_MODIFICATION | 43 | -0.47463846 | -1.7790337 | 0.017612524 |
| GO_DNA_STRAND_ELONGATION | 29 | -0.7816623 | -1.7741026 | 0.003738318 |
| GO_REGULATION_OF_CELL_CYCLE_G1_S_PHASE_TRANSITION | 138 | -0.40286484 | -1.7723118 | 0.006072875 |
| GO_REGULATION_OF_CELL_CYCLE_ARREST | 98 | -0.4571365 | -1.7680124 | 0.005905512 |
| GO_CELL_DIFFERENTIATION_INVOLVED_IN_EMBRYONIC_PLACENTA_DEVELOPMENT | 25 | -0.5899992 | -1.767117 | 0.009746589 |
| GO_REGULATION_OF_LIGASE_ACTIVITY | 124 | -0.47801855 | -1.7670078 | 0.02851711 |
| GO_CORTICAL_CYTOSKELETON_ORGANIZATION | 34 | -0.52311313 | -1.7660712 | 0.010162601 |
| GO_POSITIVE_REGULATION_OF_CHROMOSOME_ORGANIZATION | 133 | -0.3791248 | -1.7645708 | 0.01532567 |
| GO_POSITIVE_REGULATION_OF_PROTEIN_MODIFICATION_BY_SMALL_PROTEIN_CONJUGATION_OR_REMOVAL | 183 | -0.39213145 | -1.7641594 | 0.020952381 |
| GO_PROTEIN_POLYUBIQUITINATION | 234 | -0.336086 | -1.7627983 | 0.01724138 |
| GO_NEGATIVE_REGULATION_OF_DNA_METABOLIC_PROCESS | 100 | -0.4265864 | -1.7612077 | 0.013435701 |
| GO_CENTRIOLE_ASSEMBLY | 18 | -0.7152694 | -1.7582533 | 0.008064516 |
| GO_NEGATIVE_REGULATION_OF_PROTEIN_CATABOLIC_PROCESS | 102 | -0.3552996 | -1.7527673 | 0.001968504 |
| GO_ANAPHASE_PROMOTING_COMPLEX_DEPENDENT_CATABOLIC_PROCESS | 75 | -0.61550266 | -1.7511805 | 0.027777778 |
| GO_REGULATION_OF_CENTROSOME_DUPLICATION | 31 | -0.58429843 | -1.7505678 | 0.011811024 |
| GO_FEMALE_MEIOTIC_DIVISION | 21 | -0.67185855 | -1.7491484 | 0.00998004 |
| GO_POSITIVE_REGULATION_OF_TELOMERE_MAINTENANCE | 42 | -0.46721557 | -1.747931 | 0.02366864 |
| GO_RNA_POLYADENYLATION | 27 | -0.5271558 | -1.7471336 | 0.039923955 |
| GO_NEGATIVE_REGULATION_OF_DNA_REPLICATION | 48 | -0.50289816 | -1.7462021 | 0.01984127 |
| GO_POSITIVE_REGULATION_OF_G1_S_TRANSITION_OF_MITOTIC_CELL_CYCLE | 24 | -0.54190236 | -1.744025 | 0.017892644 |
| GO_RNA_LOCALIZATION | 169 | -0.37013897 | -1.741823 | 0.04528302 |
| GO_DNA_STRAND_ELONGATION_INVOLVED_IN_DNA_REPLICATION | 25 | -0.794668 | -1.7339776 | 0.003759399 |
| GO_ORGANELLE_LOCALIZATION | 390 | -0.33129835 | -1.7310216 | 0 |
| GO_STRAND_DISPLACEMENT | 25 | -0.8040322 | -1.7305461 | 0 |
| GO_POSITIVE_REGULATION_OF_DNA_BIOSYNTHETIC_PROCESS | 57 | -0.46863246 | -1.729786 | 0.023529412 |
| GO_NEGATIVE_REGULATION_OF_HISTONE_METHYLATION | 15 | -0.6200555 | -1.7259741 | 0.015779093 |
| GO_POSITIVE_REGULATION_OF_DNA_METABOLIC_PROCESS | 165 | -0.4042051 | -1.7195657 | 0.002012072 |
| GO_NUCLEOTIDE_EXCISION_REPAIR_DNA_INCISION | 39 | -0.50949925 | -1.7168323 | 0.03314917 |
| GO_ERROR_FREE_TRANSLESION_SYNTHESIS | 17 | -0.67763746 | -1.712771 | 0.01682243 |
| GO_POSITIVE_REGULATION_OF_CELL_CYCLE_G2_M_PHASE_TRANSITION | 17 | -0.5674387 | -1.7083726 | 0.021153847 |
| GO_POSITIVE_REGULATION_OF_CELL_DIVISION | 107 | -0.48908064 | -1.7068553 | 0.001988072 |
| GO_MALE_MEIOSIS | 33 | -0.619789 | -1.7049869 | 0.006012024 |
| GO_POSITIVE_REGULATION_OF_TELOMERE_CAPPING | 15 | -0.5869517 | -1.7041389 | 0.01996008 |
| GO_REGULATION_OF_DNA_RECOMBINATION | 49 | -0.5467267 | -1.7031666 | 0.026156941 |
| GO_SYNAPTONEMAL_COMPLEX_ORGANIZATION | 18 | -0.6901225 | -1.7022282 | 0.013487476 |
| GO_PEPTIDYL_LYSINE_MODIFICATION | 273 | -0.35446882 | -1.700698 | 0.040618956 |
| GO_NUCLEIC_ACID_PHOSPHODIESTER_BOND_HYDROLYSIS | 223 | -0.38956767 | -1.7006539 | 0.02264151 |
| GO_SYNAPSIS | 27 | -0.6290744 | -1.6984136 | 0.013513514 |
| GO_PROTEIN_UBIQUITINATION_INVOLVED_IN_UBIQUITIN_DEPENDENT_PROTEIN_CATABOLIC_PROCESS | 126 | -0.38382673 | -1.6982111 | 0.02366864 |
| GO_PROTEASOMAL_PROTEIN_CATABOLIC_PROCESS | 260 | -0.35393998 | -1.6969198 | 0.0415879 |
| GO_NEGATIVE_REGULATION_OF_HISTONE_MODIFICATION | 34 | -0.4427296 | -1.6930442 | 0.027722772 |
| GO_POSITIVE_REGULATION_OF_RESPONSE_TO_DNA_DAMAGE_STIMULUS | 55 | -0.43315163 | -1.6902695 | 0.007797271 |
| GO_DNA_DOUBLE_STRAND_BREAK_PROCESSING | 17 | -0.66872627 | -1.6898859 | 0.022944551 |
| GO_TELOMERE_MAINTENANCE_VIA_TELOMERE_LENGTHENING | 24 | -0.5625111 | -1.6870826 | 0.02964427 |
| GO_SOMATIC_CELL_DNA_RECOMBINATION | 31 | -0.5298299 | -1.6776072 | 0.019193858 |
| GO_POSITIVE_REGULATION_OF_HISTONE_METHYLATION | 25 | -0.5233452 | -1.6773574 | 0.036893204 |
| GO_REGULATION_OF_TRANSCRIPTION_INVOLVED_IN_G1_S_TRANSITION_OF_MITOTIC_CELL_CYCLE | 25 | -0.76893467 | -1.6740521 | 0.009689922 |
| GO_DNA_DEPENDENT_DNA_REPLICATION_MAINTENANCE_OF_FIDELITY | 18 | -0.7420729 | -1.6655046 | 0.021956088 |
| GO_ANTIGEN_PROCESSING_AND_PRESENTATION_OF_PEPTIDE_OR_POLYSACCHARIDE_ANTIGEN_VIA_MHC_CLASS_II | 86 | -0.43383107 | -1.655918 | 0.0332681 |
| GO_CYTOPLASMIC_SEQUESTERING_OF_PROTEIN | 40 | -0.45491776 | -1.6531233 | 0.046692606 |
| GO_REGULATION_OF_DOUBLE_STRAND_BREAK_REPAIR_VIA_HOMOLOGOUS_RECOMBINATION | 16 | -0.7134759 | -1.6514646 | 0.015810277 |
| GO_RESPONSE_TO_X_RAY | 30 | -0.5630802 | -1.6459798 | 0.035785288 |
| GO_POSITIVE_REGULATION_OF_DNA_REPLICATION | 82 | -0.4042701 | -1.6446731 | 0.009689922 |
| GO_CELLULAR_RESPONSE_TO_IONIZING_RADIATION | 49 | -0.45385554 | -1.631832 | 0.027237354 |
| GO_CELL_SEPARATION_AFTER_CYTOKINESIS | 17 | -0.5754076 | -1.6253787 | 0.03663004 |
| GO_RESPONSE_TO_GAMMA_RADIATION | 48 | -0.47517863 | -1.6250004 | 0.01996008 |
| GO_POSITIVE_REGULATION_OF_TELOMERE_MAINTENANCE_VIA_TELOMERE_LENGTHENING | 32 | -0.47552243 | -1.6249145 | 0.037401576 |
| GO_ESTABLISHMENT_OF_MITOTIC_SPINDLE_ORIENTATION | 19 | -0.5296979 | -1.6222882 | 0.03868472 |
| GO_NUCLEAR_IMPORT | 125 | -0.3346193 | -1.6206826 | 0.031894933 |
| GO_G1_DNA_DAMAGE_CHECKPOINT | 68 | -0.4201567 | -1.6136433 | 0.036608864 |
| GO_PROTEIN_LOCALIZATION_TO_CYTOSKELETON | 28 | -0.54136986 | -1.6112503 | 0.032128513 |
| GO_TRNA_METHYLATION | 19 | -0.60349107 | -1.6111431 | 0.040665433 |
| GO_NUCLEOSIDE_MONOPHOSPHATE_BIOSYNTHETIC_PROCESS | 57 | -0.45991907 | -1.6006256 | 0.04859813 |
| GO_REGULATION_OF_CELLULAR_PROTEIN_CATABOLIC_PROCESS | 260 | -0.3144975 | -1.5995458 | 0.049056605 |
| GO_POSITIVE_REGULATION_OF_DNA_REPAIR | 31 | -0.4560948 | -1.5959101 | 0.030710172 |
| GO_MAINTENANCE_OF_LOCATION | 132 | -0.36179858 | -1.5886865 | 0.014198783 |
| GO_CELL_AGING | 63 | -0.3883657 | -1.563304 | 0.030710172 |
| GO_HIPPOCAMPUS_DEVELOPMENT | 64 | -0.42272943 | -1.5623801 | 0.029761905 |
| GO_REGULATION_OF_HISTONE_H3_K4_METHYLATION | 22 | -0.49618706 | -1.5619197 | 0.04389313 |
| GO_ORGANELLE_ASSEMBLY | 439 | -0.32357973 | -1.5607114 | 0.03327172 |
| GO_N_TERMINAL_PROTEIN_AMINO_ACID_MODIFICATION | 23 | -0.45196563 | -1.5545529 | 0.046783626 |
| GO_MITOCHONDRIAL_FUSION | 15 | -0.5527616 | -1.5427046 | 0.043396227 |
| GO_PEPTIDYL_SERINE_MODIFICATION | 143 | -0.34587568 | -1.5426476 | 0.03285421 |
| GO_RESPONSE_TO_RADIATION | 370 | -0.31794706 | -1.540938 | 0.007905139 |
| GO_NEGATIVE_REGULATION_OF_GENE_SILENCING | 18 | -0.5161232 | -1.5298076 | 0.042226486 |
| GO_SIGNAL_TRANSDUCTION_BY_P53_CLASS_MEDIATOR | 119 | -0.36165652 | -1.5292908 | 0.03244275 |
