# Supplementary material for: Immune landscape and a promising immune prognostic model associated with TP53 in early‐stage lung adenocarcinoma
Source: Cancer Med. 2020 Dec 12;10(3):806–23. doi: 10.1002/cam4.3655 (PMC7897963; doi:10.1002/cam4.3655)
Supplement: Supplementary file 3 — Table S3 [file CAM4-10-806-s003.docx]

**Supplementary table 3**: Immune-associated DEGs between TP53^MUT^ and TP53^WT^ early-stage LUAD.

| Gene | logFC | logCPM | LR | P Value | FDR |
| --- | --- | --- | --- | --- | --- |
| *FCN3* | -4.45659 | 4.995506 | 797.1073 | 2.3E-175 | 2.8E-172 |
| *MASP1* | -2.63665 | 2.062195 | 308.3499 | 5E-69 | 3.53E-67 |
| *C5AR1* | -1.89702 | 5.085967 | 276.7835 | 3.77E-62 | 2.13E-60 |
| *CFP* | -2.0206 | 2.270324 | 274.3019 | 1.31E-61 | 7.23E-60 |
| *VSIG4* | -2.32027 | 6.195385 | 267.4541 | 4.07E-60 | 2.03E-58 |
| *A2M* | -1.81442 | 10.44656 | 247.5851 | 8.73E-56 | 3.63E-54 |
| *CFD* | -2.28312 | 5.09762 | 245.8443 | 2.09E-55 | 8.46E-54 |
| *IL6* | -2.7664 | 3.770806 | 242.7745 | 9.77E-55 | 3.86E-53 |
| *HLA-E* | -1.2739 | 10.01279 | 220.1104 | 8.56E-50 | 2.69E-48 |
| *GAB1* | -1.27634 | 4.833444 | 206.1176 | 9.66E-47 | 2.64E-45 |
| *PID1* | -1.87156 | 4.333933 | 198.2277 | 5.09E-45 | 1.31E-43 |
| *GCNT3* | 5.222148 | 5.670853 | 173.7781 | 1.11E-39 | 2.24E-38 |
| *IL33* | -2.01858 | 5.211516 | 173.1004 | 1.56E-39 | 3.12E-38 |
| *PROS1* | -1.44469 | 5.762932 | 171.0231 | 4.42E-39 | 8.65E-38 |
| *CAMP* | -2.57481 | -0.19857 | 169.7189 | 8.52E-39 | 1.64E-37 |
| *SELPLG* | -1.3977 | 5.505538 | 167.758 | 2.28E-38 | 4.33E-37 |
| *SERPING1* | -1.33379 | 8.485946 | 164.7271 | 1.05E-37 | 1.93E-36 |
| *IL1RL1* | -2.77974 | 3.690823 | 163.1607 | 2.31E-37 | 4.2E-36 |
| *HSPD1* | 1.379921 | 8.379241 | 153.9315 | 2.4E-35 | 3.96E-34 |
| *C1QA* | -1.50784 | 7.922877 | 128.2526 | 9.88E-30 | 1.19E-28 |
| *SNCA* | -1.4421 | 1.769554 | 128.0815 | 1.08E-29 | 1.29E-28 |
| *C1QB* | -1.5317 | 8.208129 | 122.729 | 1.6E-28 | 1.79E-27 |
| *IL31RA* | 4.134883 | 1.882694 | 120.0096 | 6.3E-28 | 6.79E-27 |
| *FCN1* | -1.67234 | 4.047597 | 111.1974 | 5.36E-26 | 5.08E-25 |
| *IL36RN* | 6.255154 | 1.652024 | 108.17 | 2.47E-25 | 2.26E-24 |
| *ZP3* | 2.047095 | 2.723659 | 107.1011 | 4.23E-25 | 3.82E-24 |
| *CCL2* | -1.43414 | 5.765496 | 105.6177 | 8.94E-25 | 7.88E-24 |
| *CFB* | 1.957073 | 5.075325 | 90.09797 | 2.27E-21 | 1.56E-20 |
| *IGLL5* | 2.509872 | 7.020516 | 88.13889 | 6.1E-21 | 4.07E-20 |
| *C8B* | -2.56856 | 2.047458 | 87.7957 | 7.26E-21 | 4.82E-20 |
| *FGB* | 9.028662 | 7.922696 | 87.46692 | 8.57E-21 | 5.65E-20 |
| *C7* | -1.73653 | 6.827827 | 83.87761 | 5.26E-20 | 3.27E-19 |
| *VIP* | -1.477 | -2.01009 | 82.41507 | 1.1E-19 | 6.7E-19 |
| *COLEC10* | -2.00081 | -0.44919 | 77.17283 | 1.57E-18 | 8.76E-18 |
| *PLA2G1B* | -2.11429 | 2.581503 | 74.22194 | 6.98E-18 | 3.7E-17 |
| *IL13* | -1.69123 | -2.86293 | 71.51915 | 2.75E-17 | 1.39E-16 |
| ***ENTPD2*** | **2.230584** | **2.089113** | **70.36701** | **4.92E-17** | **2.42E-16** |
| *CALCA* | 8.635325 | 7.851691 | 69.56766 | 7.38E-17 | 3.57E-16 |
| *MUC5B* | 4.043683 | 9.4728 | 67.52849 | 2.08E-16 | 9.68E-16 |
| ***MIF*** | **1.299094** | **5.5596** | **65.48533** | **5.85E-16** | **2.63E-15** |
| *IGLL1* | 2.850902 | -1.32032 | 64.05129 | 1.21E-15 | 5.3E-15 |
| *C6* | -1.9599 | 2.384918 | 61.6331 | 4.14E-15 | 1.73E-14 |
| *BPIFA1* | 5.251588 | 8.83006 | 59.81418 | 1.04E-14 | 4.22E-14 |
| *PTGIS* | -1.19551 | 5.086644 | 55.32364 | 1.02E-13 | 3.8E-13 |
| *C1QC* | -1.04062 | 7.851077 | 54.96831 | 1.22E-13 | 4.53E-13 |
| *KRT1* | -1.7249 | -1.01577 | 51.91644 | 5.79E-13 | 2.02E-12 |
| *LBP* | 4.991776 | 3.048047 | 51.79502 | 6.16E-13 | 2.14E-12 |
| *C4BPB* | 2.29192 | 3.442515 | 49.31397 | 2.18E-12 | 7.28E-12 |
| *C8G* | 1.827055 | 0.192616 | 46.56181 | 8.88E-12 | 2.82E-11 |
| *FGA* | 3.974277 | 8.567229 | 44.47199 | 2.58E-11 | 7.89E-11 |
| *SUSD4* | 1.319121 | 4.021371 | 36.33787 | 1.66E-09 | 4.35E-09 |
| *HPX* | 1.748101 | 1.259665 | 33.93867 | 5.69E-09 | 1.42E-08 |
| *CXCL13* | 1.630149 | 5.06517 | 30.7358 | 2.96E-08 | 6.93E-08 |
| *CHI3L1* | 1.499975 | 7.647903 | 29.52282 | 5.53E-08 | 1.27E-07 |
| *ALDOB* | 1.955338 | 1.634453 | 23.29751 | 1.39E-06 | 2.8E-06 |
| *PLA2G10* | 1.371341 | 2.528903 | 22.19062 | 2.47E-06 | 4.86E-06 |
| *SPINK5* | 1.510266 | 4.837879 | 21.01178 | 4.56E-06 | 8.77E-06 |
| *RNASE7* | 2.10505 | -1.67855 | 20.71857 | 5.32E-06 | 1.01E-05 |
| *SPACA3* | 1.619928 | -1.97962 | 20.71503 | 5.33E-06 | 1.02E-05 |
| *VTN* | 2.073064 | 3.721103 | 20.02557 | 7.64E-06 | 1.44E-05 |
| *SLPI* | -1.00538 | 8.907574 | 19.29995 | 1.12E-05 | 2.07E-05 |
| *C8A* | 2.402154 | 0.315797 | 19.28102 | 1.13E-05 | 2.09E-05 |
| *DEFB1* | 1.659533 | 2.996057 | 18.73997 | 1.5E-05 | 2.74E-05 |
| *LTF* | 1.322917 | 6.911477 | 17.63171 | 2.68E-05 | 4.8E-05 |
| *SLC7A2* | 1.103524 | 6.940835 | 14.66712 | 0.000128 | 0.000215 |
| *PGC* | 1.662069 | 10.55642 | 10.60052 | 0.001131 | 0.001727 |
| *CFHR1* | 1.014242 | -2.79198 | 6.053458 | 0.013879 | 0.01877 |
