# Supplementary material for: Immune landscape and a promising immune prognostic model associated with TP53 in early‐stage lung adenocarcinoma
Source: Cancer Med. 2020 Dec 12;10(3):806–23. doi: 10.1002/cam4.3655 (PMC7897963; doi:10.1002/cam4.3655)
Supplement: Supplementary file 4 — Table S4 [file CAM4-10-806-s004.docx]

**Supplementary table 4:** The GSEA results of early-stage LUAD patients with low risk score.

| NAME | SIZE | ES | NES | NOM p-val |
| --- | --- | --- | --- | --- |
| GO_CELLULAR_EXTRAVASATION | 24 | 0.6994414 | 1.7168324 | 0.008016032 |
| GO_NEGATIVE_REGULATION_OF_POTASSIUM_ION_TRANSPORT | 29 | 0.6566993 | 1.6808568 | 0.007590133 |
| GO_LIPID_PHOSPHORYLATION | 93 | 0.47204596 | 1.6548797 | 0.0056926 |
| GO_CELLULAR_RESPONSE_TO_GROWTH_HORMONE_STIMULUS | 17 | 0.6169484 | 1.6535225 | 0.024 |
| GO_STRIATED_MUSCLE_ADAPTATION | 21 | 0.60985297 | 1.6534896 | 0.015503876 |
| GO_POSITIVE_REGULATION_OF_NEUROLOGICAL_SYSTEM_PROCESS | 17 | 0.64323187 | 1.6350522 | 0.011881189 |
| GO_REGULATION_OF_CARDIAC_MUSCLE_CONTRACTION_BY_CALCIUM_ION_SIGNALING | 23 | 0.62127745 | 1.6271236 | 0.019267824 |
| GO_RESPONSE_TO_STEROL | 22 | 0.5863357 | 1.6154684 | 0.018480493 |
| GO_RESPONSE_TO_CAFFEINE | 17 | 0.59089845 | 1.6123197 | 0.017821781 |
| GO_PHOSPHATIDYLINOSITOL_DEPHOSPHORYLATION | 21 | 0.54039013 | 1.6102316 | 0.016563147 |
| GO_REGULATION_OF_RELEASE_OF_SEQUESTERED_CALCIUM_ION_INTO_CYTOSOL_BY_SARCOPLASMIC_RETICULUM | 24 | 0.6335522 | 1.6063207 | 0.020833334 |
| GO_PHOSPHATIDYLINOSITOL_METABOLIC_PROCESS | 181 | 0.3950608 | 1.605532 | 0.003921569 |
| GO_MYOFIBRIL_ASSEMBLY | 41 | 0.58719265 | 1.6030327 | 0.019417476 |
| GO_POSITIVE_REGULATION_OF_SMALL_GTPASE_MEDIATED_SIGNAL_TRANSDUCTION | 36 | 0.52053344 | 1.5974947 | 0.017374517 |
| GO_CORONARY_VASCULATURE_DEVELOPMENT | 35 | 0.5580075 | 1.5955647 | 0.038240917 |
| GO_PHOSPHOLIPID_DEPHOSPHORYLATION | 22 | 0.53716666 | 1.594782 | 0.023206752 |
| GO_NEGATIVE_REGULATION_OF_CATECHOLAMINE_SECRETION | 15 | 0.73105454 | 1.5911468 | 0.010121457 |
| GO_PURINERGIC_NUCLEOTIDE_RECEPTOR_SIGNALING_PATHWAY | 22 | 0.69961977 | 1.5880477 | 0.034068137 |
| GO_REGULATION_OF_CARDIAC_MUSCLE_CONTRACTION_BY_REGULATION_OF_THE_RELEASE_OF_SEQUESTERED_CALCIUM_ION | 19 | 0.6655127 | 1.5821333 | 0.027131783 |
| GO_CELLULAR_RESPONSE_TO_PROSTAGLANDIN_E_STIMULUS | 18 | 0.60038775 | 1.5769377 | 0.03696498 |
| **GO_REGULATION_OF_ADAPTIVE_**  **IMMUNE_RESPONSE** | **33** | **0.608928** | **1.5760256** | **0.040733196** |
| **GO_CYTOKINE_PRODUCTION_**  **INVOLVED_IN_IMMUNE_RESPONSE** | **16** | **0.61213475** | **1.573144** | **0.03448276** |
| GO_MODULATION_OF_GROWTH_OF_SYMBIONT_INVOLVED_IN_INTERACTION_WITH_HOST | 15 | 0.6946171 | 1.5654048 | 0.04375 |
| GO_NEUROMUSCULAR_JUNCTION_DEVELOPMENT | 34 | 0.5382322 | 1.5631363 | 0.036053132 |
| GO_REGULATION_OF_CALCIUM_MEDIATED_SIGNALING | 74 | 0.5196852 | 1.5571829 | 0.040899795 |
| GO_ACTIVATION_OF_PHOSPHOLIPASE_C_ACTIVITY | 27 | 0.63509816 | 1.5549109 | 0.026565464 |
| **GO_REGULATION_OF_PRODUCTION_OF_MOLECULAR_MEDIATOR_OF_IMMUNE_RESPONSE** | **26** | **0.5830748** | **1.5539079** | **0.037181996** |
| GO_REGULATION_OF_PLATELET_AGGREGATION | 16 | 0.64799476 | 1.5513546 | 0.033962265 |
| GO_PURINERGIC_RECEPTOR_SIGNALING_PATHWAY | 27 | 0.6559757 | 1.5490649 | 0.044715445 |
| **GO_THYMIC_T_CELL_SELECTION** | **19** | **0.6944178** | **1.5487921** | **0.04809619** |
| GO_REGULATION_OF_POTASSIUM_ION_TRANSPORT | 78 | 0.52867395 | 1.5469522 | 0.036538463 |
| GO_HETEROPHILIC_CELL_CELL_ADHESION_VIA_PLASMA_MEMBRANE_CELL_ADHESION_MOLECULES | 35 | 0.6039673 | 1.5459442 | 0.04109589 |
| GO_MUSCLE_CELL_CELLULAR_HOMEOSTASIS | 16 | 0.5447507 | 1.541906 | 0.045714285 |
| GO_REGULATION_OF_NON_CANONICAL_WNT_SIGNALING_PATHWAY | 19 | 0.5767834 | 1.5411949 | 0.039215688 |
| GO_CYTOSOLIC_CALCIUM_ION_TRANSPORT | 50 | 0.55685925 | 1.5406502 | 0.046277665 |
| GO_FOREBRAIN_CELL_MIGRATION | 54 | 0.50808007 | 1.539326 | 0.04158416 |
| GO_POSITIVE_REGULATION_OF_VASODILATION | 29 | 0.5623141 | 1.5386207 | 0.02661597 |
| GO_LONG_TERM_MEMORY | 27 | 0.55412865 | 1.5369678 | 0.032323234 |
| GO_LONG_TERM_SYNAPTIC_POTENTIATION | 37 | 0.4951399 | 1.5349671 | 0.03448276 |
| GO_POSITIVE_REGULATION_OF_MYOBLAST_DIFFERENTIATION | 15 | 0.5889709 | 1.5333481 | 0.045833334 |
| GO_MUSCLE_ADAPTATION | 26 | 0.5366054 | 1.524637 | 0.036217302 |
| GO_NEGATIVE_REGULATION_OF_AUTOPHAGY | 51 | 0.37529007 | 1.5177611 | 0.034343433 |
| GO_RESPONSE_TO_PROSTAGLANDIN_E | 25 | 0.5775362 | 1.5158395 | 0.045081966 |
| GO_SEGMENT_SPECIFICATION | 15 | 0.6291253 | 1.5151693 | 0.041420117 |
| GO_REGULATION_OF_PHOSPHOLIPASE_C_ACTIVITY | 39 | 0.6050057 | 1.5099168 | 0.049407113 |
| GO_REGULATION_OF_POTASSIUM_ION_TRANSMEMBRANE_TRANSPORTER_ACTIVITY | 40 | 0.54309916 | 1.5072174 | 0.046277665 |
| GO_CALCIUM_MEDIATED_SIGNALING | 82 | 0.4845448 | 1.5003713 | 0.04183267 |
| GO_REGULATION_OF_ADENYLATE_CYCLASE_ACTIVITY | 63 | 0.50824493 | 1.4997973 | 0.02734375 |
| GO_LYSOSOME_LOCALIZATION | 22 | 0.5031485 | 1.498667 | 0.04117647 |
| GO_REGULATION_OF_TYROSINE_PHOSPHORYLATION_OF_STAT5_PROTEIN | 16 | 0.5866637 | 1.4944315 | 0.04743083 |
| GO_NEGATIVE_REGULATION_OF_MUSCLE_CONTRACTION | 20 | 0.62008333 | 1.4942276 | 0.04761905 |
| GO_SECOND_MESSENGER_MEDIATED_SIGNALING | 144 | 0.46403012 | 1.4887296 | 0.044061303 |
| GO_INOSITOL_LIPID_MEDIATED_SIGNALING | 110 | 0.44256774 | 1.488576 | 0.04054054 |
| GO_ACTOMYOSIN_STRUCTURE_ORGANIZATION | 69 | 0.45176914 | 1.4855317 | 0.049309663 |
| GO_POSITIVE_REGULATION_OF_ADENYLATE_CYCLASE_ACTIVITY | 45 | 0.5216218 | 1.4799372 | 0.031434186 |
| GO_ACTIVATION_OF_ADENYLATE_CYCLASE_ACTIVITY | 38 | 0.5369286 | 1.4797553 | 0.03816794 |
| GO_STRIATED_MUSCLE_CELL_DIFFERENTIATION | 152 | 0.41320285 | 1.4790281 | 0.039848197 |
| GO_NEGATIVE_REGULATION_OF_SYNAPTIC_TRANSMISSION | 57 | 0.4746818 | 1.474319 | 0.047904193 |
| GO_G_PROTEIN_COUPLED_RECEPTOR_SIGNALING_PATHWAY_COUPLED_TO_CYCLIC_NUCLEOTIDE_SECOND_MESSENGER | 136 | 0.48657894 | 1.4717147 | 0.042168673 |
| GO_NEGATIVE_REGULATION_OF_CALCIUM_ION_TRANSPORT | 46 | 0.44148815 | 1.4648854 | 0.041015625 |
| GO_MUSCLE_CELL_DEVELOPMENT | 109 | 0.4420727 | 1.4578233 | 0.04761905 |
| GO_MEMBRANE_DEPOLARIZATION_DURING_ACTION_POTENTIAL | 37 | 0.5540192 | 1.4565132 | 0.049808428 |
| GO_REGULATION_OF_DELAYED_RECTIFIER_POTASSIUM_CHANNEL_ACTIVITY | 17 | 0.5927854 | 1.4556389 | 0.047244094 |
| GO_MEMBRANE_DEPOLARIZATION | 54 | 0.50894326 | 1.4483399 | 0.049309663 |
| GO_POSITIVE_REGULATION_OF_DENDRITE_DEVELOPMENT | 62 | 0.41108721 | 1.4358665 | 0.048780486 |
| GO_LIPID_MODIFICATION | 189 | 0.33401972 | 1.4335953 | 0.026768642 |
| GO_GLYCEROPHOSPHOLIPID_METABOLIC_PROCESS | 276 | 0.31000203 | 1.3855147 | 0.0332681 |
