# Supplementary material for: Immune landscape and a promising immune prognostic model associated with TP53 in early‐stage lung adenocarcinoma
Source: Cancer Med. 2020 Dec 12;10(3):806–23. doi: 10.1002/cam4.3655 (PMC7897963; doi:10.1002/cam4.3655)
Supplement: Supplementary file 5 — Table S5 [file CAM4-10-806-s005.docx]

**Supplementary table 5:** The GSEA results of early-stage LUAD patients with high risk score.

| NAME | SIZE | ES | NES | NOM p-val |
| --- | --- | --- | --- | --- |
| GO_ESTABLISHMENT_OF_PROTEIN_LOCALIZATION_TO_ORGANELLE | 339 | -0.43671986 | -2.180267 | 0.001915709 |
| GO_VIRION_ASSEMBLY | 36 | -0.57177365 | -2.1597857 | 0 |
| GO_NUCLEOSIDE_TRIPHOSPHATE_METABOLIC_PROCESS | 194 | -0.5437376 | -2.1242087 | 0 |
| GO_PROTEIN_TARGETING | 384 | -0.38756326 | -2.1203206 | 0.001984127 |
| GO_ESTABLISHMENT_OF_PROTEIN_LOCALIZATION_TO_MEMBRANE | 256 | -0.4083171 | -2.1078227 | 0.00814664 |
| GO_NUCLEOSIDE_MONOPHOSPHATE_METABOLIC_PROCESS | 206 | -0.5301576 | -2.0840886 | 0 |
| GO_MACROMOLECULAR_COMPLEX_DISASSEMBLY | 172 | -0.5620455 | -2.0509608 | 0 |
| GO_MULTI_ORGANISM_ORGANELLE_ORGANIZATION | 23 | -0.64899886 | -2.0429537 | 0 |
| GO_MULTIVESICULAR_BODY_ORGANIZATION | 30 | -0.60041106 | -2.0406363 | 0.001956947 |
| GO_MITOCHONDRIAL_TRANSPORT | 142 | -0.48493496 | -2.02734 | 0 |
| GO_AMIDE_BIOSYNTHETIC_PROCESS | 459 | -0.5041179 | -2.023401 | 0.001949318 |
| GO_MITOCHONDRIAL_MEMBRANE_ORGANIZATION | 87 | -0.48440495 | -2.0208197 | 0 |
| GO_PROTEIN_TARGETING_TO_MEMBRANE | 152 | -0.4929498 | -1.9973695 | 0.022312373 |
| GO_CELLULAR_PROTEIN_COMPLEX_DISASSEMBLY | 118 | -0.6395203 | -1.9773935 | 0 |
| GO_PROTEIN_LOCALIZATION_TO_MITOCHONDRION | 62 | -0.57010424 | -1.9586916 | 0 |
| GO_TRANSLATIONAL_ELONGATION | 108 | -0.70641875 | -1.9518907 | 0 |
| GO_MONOSACCHARIDE_BIOSYNTHETIC_PROCESS | 50 | -0.5385338 | -1.9471498 | 0.005859375 |
| GO_GLYCOSYL_COMPOUND_METABOLIC_PROCESS | 324 | -0.43523154 | -1.9423306 | 0 |
| GO_KETONE_BIOSYNTHETIC_PROCESS | 21 | -0.6815334 | -1.9399235 | 0 |
| GO_GENERATION_OF_PRECURSOR_METABOLITES_AND_ENERGY | 262 | -0.4357321 | -1.9362174 | 0.005928854 |
| GO_PROTEIN_TRANSMEMBRANE_TRANSPORT | 49 | -0.54808664 | -1.9350243 | 0.001937985 |
| GO_RRNA_METABOLIC_PROCESS | 240 | -0.5892994 | -1.9328356 | 0.001992032 |
| GO_REGULATION_OF_MEMBRANE_PERMEABILITY | 67 | -0.4580407 | -1.9209408 | 0.005649718 |
| GO_RRNA_TRANSCRIPTION | 17 | -0.7046458 | -1.9205549 | 0 |
| GO_CELLULAR_RESPIRATION | 135 | -0.5848795 | -1.9174558 | 0.004123712 |
| GO_GLYCOSYL_COMPOUND_BIOSYNTHETIC_PROCESS | 87 | -0.48987046 | -1.9163587 | 0 |
| GO_RIBOSOME_BIOGENESIS | 287 | -0.5815444 | -1.899694 | 0.002 |
| GO_NUCLEOSIDE_TRIPHOSPHATE_BIOSYNTHETIC_PROCESS | 38 | -0.5862607 | -1.8985143 | 0.001923077 |
| GO_RNA_CATABOLIC_PROCESS | 209 | -0.4650031 | -1.8905344 | 0.035019454 |
| GO_TRANSLATIONAL_INITIATION | 142 | -0.60702634 | -1.8826852 | 0.021400778 |
| GO_PROTEIN_TARGETING_TO_MITOCHONDRION | 46 | -0.6228913 | -1.8796335 | 0.003968254 |
| GO_RIBOSOME_ASSEMBLY | 50 | -0.6288698 | -1.8794944 | 0.003898636 |
| GO_ELECTRON_TRANSPORT_CHAIN | 90 | -0.6378518 | -1.8791525 | 0.004081633 |
| GO_OXIDATIVE_PHOSPHORYLATION | 77 | -0.7098713 | -1.8777425 | 0.002004008 |
| GO_MULTI_ORGANISM_METABOLIC_PROCESS | 135 | -0.5978281 | -1.8756309 | 0.015810277 |
| GO_MITOCHONDRIAL_FUSION | 15 | -0.6488837 | -1.8728144 | 0.005813954 |
| GO_RIBONUCLEOPROTEIN_COMPLEX_BIOGENESIS | 408 | -0.52876514 | -1.8697454 | 0.002016129 |
| GO_TRANSLATIONAL_TERMINATION | 91 | -0.7118842 | -1.8627386 | 0 |
| GO_DEOXYRIBONUCLEOTIDE_METABOLIC_PROCESS | 33 | -0.6537641 | -1.8616717 | 0 |
| GO_NCRNA_METABOLIC_PROCESS | 470 | -0.5357605 | -1.8616679 | 0.002004008 |
| GO_NCRNA_PROCESSING | 349 | -0.5694179 | -1.8579805 | 0.002020202 |
| GO_NUCLEOSIDE_MONOPHOSPHATE_BIOSYNTHETIC_PROCESS | 57 | -0.5586562 | -1.8553387 | 0 |
| GO_DNA_DAMAGE_RESPONSE_DETECTION_OF_DNA_DAMAGE | 36 | -0.65065134 | -1.8550059 | 0.001941748 |
| GO_PURINE_NUCLEOSIDE_BIOSYNTHETIC_PROCESS | 61 | -0.47361153 | -1.8495297 | 0.001886793 |
| GO_PROTEIN_LOCALIZATION_TO_ENDOPLASMIC_RETICULUM | 122 | -0.5951588 | -1.8475533 | 0.02970297 |
| GO_MITOCHONDRIAL_TRANSLATION | 103 | -0.6969431 | -1.8458806 | 0 |
| GO_ENDOSOME_ORGANIZATION | 57 | -0.39121854 | -1.8453145 | 0.02226345 |
| GO_PYRIMIDINE_NUCLEOTIDE_METABOLIC_PROCESS | 43 | -0.57858586 | -1.8315434 | 0.001941748 |
| GO_ENERGY_DERIVATION_BY_OXIDATION_OF_ORGANIC_COMPOUNDS | 202 | -0.44162574 | -1.8244916 | 0.022 |
| GO_NUCLEOTIDE_PHOSPHORYLATION | 54 | -0.50903285 | -1.8196357 | 0.005802708 |
| GO_RIBONUCLEOSIDE_TRIPHOSPHATE_BIOSYNTHETIC_PROCESS | 26 | -0.5966735 | -1.8164937 | 0.003816794 |
| GO_POSITIVE_REGULATION_OF_MITOCHONDRIAL_MEMBRANE_PERMEABILITY | 17 | -0.6137267 | -1.8118106 | 0 |
| GO_MITOCHONDRIAL_TRANSMEMBRANE_TRANSPORT | 36 | -0.57880676 | -1.8095403 | 0.01004016 |
| GO_NUCLEOTIDE_SUGAR_METABOLIC_PROCESS | 33 | -0.54939646 | -1.8079369 | 0.019646365 |
| GO_REGULATION_OF_LIGASE_ACTIVITY | 124 | -0.48293275 | -1.8043716 | 0.011538462 |
| GO_MONOSACCHARIDE_CATABOLIC_PROCESS | 54 | -0.52345884 | -1.8010931 | 0 |
| GO_MITOCHONDRIAL_RESPIRATORY_CHAIN_COMPLEX_ASSEMBLY | 63 | -0.6827672 | -1.8006945 | 0 |
| GO_CELLULAR_COMPONENT_DISASSEMBLY | 475 | -0.35767138 | -1.79889 | 0.001923077 |
| GO_NUCLEAR_TRANSCRIBED_MRNA_CATABOLIC_PROCESS_NONSENSE_MEDIATED_DECAY | 117 | -0.5742221 | -1.7986083 | 0.04117647 |
| GO_APOPTOTIC_MITOCHONDRIAL_CHANGES | 55 | -0.4749039 | -1.7940058 | 0.004 |
| GO_TRANSCRIPTION_COUPLED_NUCLEOTIDE_EXCISION_REPAIR | 72 | -0.5077203 | -1.7928036 | 0.017307693 |
| GO_NEGATIVE_REGULATION_OF_PROTEIN_MODIFICATION_BY_SMALL_PROTEIN_CONJUGATION_OR_REMOVAL | 133 | -0.43415475 | -1.7927424 | 0.01908397 |
| GO_POSITIVE_REGULATION_OF_VIRAL_TRANSCRIPTION | 35 | -0.45382583 | -1.7904311 | 0.020952381 |
| GO_SPLICEOSOMAL_SNRNP_ASSEMBLY | 37 | -0.63178474 | -1.7874163 | 0.00591716 |
| GO_RETROGRADE_VESICLE_MEDIATED_TRANSPORT_GOLGI_TO_ER | 74 | -0.49265864 | -1.7862052 | 0.009596929 |
| GO_CELL_SEPARATION_AFTER_CYTOKINESIS | 17 | -0.6555901 | -1.7847549 | 0.003883495 |
| GO_RNA_CAPPING | 33 | -0.57225317 | -1.7830472 | 0.013384321 |
| GO_PEPTIDYL_PROLINE_MODIFICATION | 45 | -0.5104029 | -1.7806683 | 0.019723866 |
| GO_REGULATION_OF_INTRINSIC_APOPTOTIC_SIGNALING_PATHWAY | 131 | -0.3882447 | -1.7804694 | 0.00390625 |
| GO_RIBONUCLEOPROTEIN_COMPLEX_SUBUNIT_ORGANIZATION | 184 | -0.44917107 | -1.779212 | 0.029940119 |
| GO_RESPONSE_TO_TOPOLOGICALLY_INCORRECT_PROTEIN | 150 | -0.3514647 | -1.7788478 | 0.012371134 |
| GO_POSITIVE_REGULATION_OF_LIGASE_ACTIVITY | 104 | -0.51159036 | -1.7744864 | 0.019379845 |
| GO_PROTEIN_FOLDING | 192 | -0.38488162 | -1.7727687 | 0.01814516 |
| GO_PYRIMIDINE_NUCLEOTIDE_BIOSYNTHETIC_PROCESS | 28 | -0.59171146 | -1.7690036 | 0.003853565 |
| GO_DEOXYRIBOSE_PHOSPHATE_CATABOLIC_PROCESS | 21 | -0.66039604 | -1.7671653 | 0.002020202 |
| GO_RELEASE_OF_CYTOCHROME_C_FROM_MITOCHONDRIA | 22 | -0.59767175 | -1.7637658 | 0.006072875 |
| GO_RNA_PHOSPHODIESTER_BOND_HYDROLYSIS_ENDONUCLEOLYTIC | 53 | -0.5337363 | -1.7625529 | 0.005952381 |
| GO_PYRIMIDINE_NUCLEOSIDE_TRIPHOSPHATE_BIOSYNTHETIC_PROCESS | 17 | -0.6783576 | -1.7613645 | 0.005714286 |
| GO_NUCLEOBASE_CONTAINING_SMALL_MOLECULE_INTERCONVERSION | 21 | -0.6551506 | -1.7575297 | 0.005976096 |
| GO_MITOCHONDRIAL_RESPIRATORY_CHAIN_COMPLEX_I_BIOGENESIS | 51 | -0.6984929 | -1.7516501 | 0 |
| GO_POSITIVE_REGULATION_OF_RELEASE_OF_CYTOCHROME_C_FROM_MITOCHONDRIA | 26 | -0.5959228 | -1.7496715 | 0.005791506 |
| GO_NUCLEOSIDE_DIPHOSPHATE_METABOLIC_PROCESS | 77 | -0.45478278 | -1.7478802 | 0.003921569 |
| GO_RIBOSOMAL_SMALL_SUBUNIT_BIOGENESIS | 53 | -0.6114489 | -1.746912 | 0.018480493 |
| GO_NEGATIVE_REGULATION_OF_INTRINSIC_APOPTOTIC_SIGNALING_PATHWAY | 80 | -0.3942142 | -1.7468715 | 0.007766991 |
| GO_REGULATION_OF_PROTEIN_UBIQUITINATION_INVOLVED_IN_UBIQUITIN_DEPENDENT_PROTEIN_CATABOLIC_PROCESS | 101 | -0.46832052 | -1.7453924 | 0.032136105 |
| GO_PYRIMIDINE_NUCLEOSIDE_TRIPHOSPHATE_METABOLIC_PROCESS | 19 | -0.65032214 | -1.7448905 | 0.007707129 |
| GO_MEMBRANE_BUDDING | 110 | -0.33084348 | -1.743697 | 0.026217228 |
| GO_MITOCHONDRIAL_ELECTRON_TRANSPORT_CYTOCHROME_C_TO_OXYGEN | 16 | -0.7207506 | -1.743487 | 0.010141988 |
| GO_QUINONE_METABOLIC_PROCESS | 27 | -0.6876109 | -1.7430571 | 0.027237354 |
| GO_NUCLEOBASE_CONTAINING_SMALL_MOLECULE_METABOLIC_PROCESS | 466 | -0.3638683 | -1.7385923 | 0.001923077 |
| GO_CYTOPLASMIC_TRANSLATION | 40 | -0.60815626 | -1.733721 | 0.01980198 |
| GO_PROTEASOMAL_PROTEIN_CATABOLIC_PROCESS | 260 | -0.36957198 | -1.7299522 | 0.0251938 |
| GO_RIBOSOMAL_SMALL_SUBUNIT_ASSEMBLY | 16 | -0.7385778 | -1.7284448 | 0.01417004 |
| GO_OXIDOREDUCTION_COENZYME_METABOLIC_PROCESS | 95 | -0.43123853 | -1.7265272 | 0.009727626 |
| GO_HEXOSE_CATABOLIC_PROCESS | 45 | -0.5108651 | -1.725729 | 0.001941748 |
| GO__DE_NOVO_PROTEIN_FOLDING | 17 | -0.56725806 | -1.725212 | 0.031311154 |
| GO_NUCLEAR_TRANSCRIBED_MRNA_CATABOLIC_PROCESS_EXONUCLEOLYTIC | 30 | -0.5767851 | -1.724762 | 0.019880716 |
| GO_RESPONSE_TO_ENDOPLASMIC_RETICULUM_STRESS | 213 | -0.33384907 | -1.7236882 | 0.014 |
| GO_MATURATION_OF_SSU_RRNA_FROM_TRICISTRONIC_RRNA_TRANSCRIPT_SSU_RRNA_5_8S_RRNA_LSU_RRNA_ | 32 | -0.636336 | -1.7207667 | 0.010121457 |
| GO_CHAPERONE_MEDIATED_PROTEIN_FOLDING | 43 | -0.46101317 | -1.7194867 | 0.020283977 |
| GO_REGULATION_OF_MITOCHONDRIAL_OUTER_MEMBRANE_PERMEABILIZATION_INVOLVED_IN_APOPTOTIC_SIGNALING_PATHWAY | 41 | -0.46583793 | -1.7193435 | 0.013435701 |
| GO_CELLULAR_RESPONSE_TO_TOPOLOGICALLY_INCORRECT_PROTEIN | 110 | -0.36008957 | -1.7176745 | 0.030864198 |
| GO_REGULATION_OF_MITOCHONDRIAL_MEMBRANE_PERMEABILITY_INVOLVED_IN_APOPTOTIC_PROCESS | 21 | -0.53511393 | -1.7161154 | 0.017307693 |
| GO_RIBOSOMAL_LARGE_SUBUNIT_BIOGENESIS | 47 | -0.6293346 | -1.7130759 | 0.010060363 |
| GO_ORGANIC_CYCLIC_COMPOUND_CATABOLIC_PROCESS | 393 | -0.3396772 | -1.7114345 | 0.021868788 |
| GO_REGULATION_OF_RNA_STABILITY | 134 | -0.3257395 | -1.7108243 | 0.030425964 |
| GO_INNER_MITOCHONDRIAL_MEMBRANE_ORGANIZATION | 15 | -0.6850422 | -1.7069887 | 0.005905512 |
| GO_REGULATION_OF_NECROTIC_CELL_DEATH | 24 | -0.5123019 | -1.705948 | 0.02819549 |
| GO_NUCLEOTIDE_EXCISION_REPAIR_DNA_INCISION | 39 | -0.49503508 | -1.700283 | 0.03219697 |
| GO_ADP_METABOLIC_PROCESS | 42 | -0.47951603 | -1.698676 | 0.019305019 |
| GO_MULTI_ORGANISM_MEMBRANE_ORGANIZATION | 29 | -0.4512712 | -1.6967071 | 0.026209677 |
| GO_REGULATION_OF_RELEASE_OF_CYTOCHROME_C_FROM_MITOCHONDRIA | 40 | -0.5061333 | -1.6965123 | 0.013487476 |
| GO_DEOXYRIBONUCLEOSIDE_TRIPHOSPHATE_METABOLIC_PROCESS | 16 | -0.64977026 | -1.6949668 | 0.01532567 |
| GO_POSITIVE_REGULATION_OF_PROTEIN_OLIGOMERIZATION | 17 | -0.64588267 | -1.6940048 | 0.020449897 |
| GO_PYRIMIDINE_DEOXYRIBONUCLEOTIDE_METABOLIC_PROCESS | 16 | -0.69087726 | -1.692732 | 0.00990099 |
| GO_ESTABLISHMENT_OF_PROTEIN_LOCALIZATION_TO_ENDOPLASMIC_RETICULUM | 103 | -0.6303741 | -1.6910722 | 0.037698414 |
| GO_PYRIMIDINE_CONTAINING_COMPOUND_BIOSYNTHETIC_PROCESS | 37 | -0.53175527 | -1.6862488 | 0.009596929 |
| GO_NUCLEOTIDE_EXCISION_REPAIR | 110 | -0.4338997 | -1.6856054 | 0.032945737 |
| GO_RRNA_METHYLATION | 15 | -0.6517733 | -1.68456 | 0.013513514 |
| GO_PURINE_CONTAINING_COMPOUND_METABOLIC_PROCESS | 343 | -0.35236043 | -1.6839337 | 0.00967118 |
| GO_MONOSACCHARIDE_METABOLIC_PROCESS | 172 | -0.3896351 | -1.6772646 | 0.005825243 |
| GO_BASE_EXCISION_REPAIR | 39 | -0.5849665 | -1.675959 | 0.038610037 |
| GO_NCRNA_TRANSCRIPTION | 80 | -0.4764245 | -1.674302 | 0.033663366 |
| GO_POSITIVE_REGULATION_OF_MITOCHONDRIAL_OUTER_MEMBRANE_PERMEABILIZATION_INVOLVED_IN_APOPTOTIC_SIGNALING_PATHWAY | 35 | -0.46757418 | -1.6726748 | 0.030303031 |
| GO_TRNA_PROCESSING | 100 | -0.5735847 | -1.6722318 | 0.02964427 |
| GO_HEXOSE_METABOLIC_PROCESS | 141 | -0.37548482 | -1.6696138 | 0.013435701 |
| GO_RIBOSOMAL_LARGE_SUBUNIT_ASSEMBLY | 23 | -0.6262737 | -1.6609788 | 0.021568628 |
| GO_REGULATION_OF_VIRAL_TRANSCRIPTION | 57 | -0.3782486 | -1.6584032 | 0.03307393 |
| GO_COFACTOR_BIOSYNTHETIC_PROCESS | 148 | -0.41399235 | -1.6559806 | 0.013944224 |
| GO_NEGATIVE_REGULATION_OF_CELL_CYCLE_ARREST | 17 | -0.54770637 | -1.6549686 | 0.011952192 |
| GO_PURINE_NUCLEOSIDE_MONOPHOSPHATE_BIOSYNTHETIC_PROCESS | 37 | -0.5130845 | -1.654217 | 0.024390243 |
| GO_PROTEIN_K11_LINKED_UBIQUITINATION | 26 | -0.553424 | -1.6540573 | 0.01372549 |
| GO_TRNA_METABOLIC_PROCESS | 144 | -0.5632717 | -1.653993 | 0.03807615 |
| GO_POSITIVE_REGULATION_OF_PROTEIN_MODIFICATION_BY_SMALL_PROTEIN_CONJUGATION_OR_REMOVAL | 183 | -0.3538775 | -1.6520398 | 0.04660194 |
| GO_CYTOPLASMIC_SEQUESTERING_OF_PROTEIN | 40 | -0.41861764 | -1.6511897 | 0.032015067 |
| GO_RRNA_MODIFICATION | 23 | -0.59206957 | -1.6501875 | 0.032818533 |
| GO_RNA_PHOSPHODIESTER_BOND_HYDROLYSIS | 105 | -0.43309918 | -1.647568 | 0.025948104 |
| GO_UBIQUITIN_DEPENDENT_PROTEIN_CATABOLIC_PROCESS_VIA_THE_MULTIVESICULAR_BODY_SORTING_PATHWAY | 16 | -0.51706237 | -1.6460382 | 0.045009784 |
| GO_RNA_MODIFICATION | 103 | -0.49138227 | -1.6446973 | 0.036679536 |
| GO_REGULATION_OF_SPINDLE_ORGANIZATION | 19 | -0.58920264 | -1.6442975 | 0.04024145 |
| GO_REGULATION_OF_PROTEIN_MODIFICATION_BY_SMALL_PROTEIN_CONJUGATION_OR_REMOVAL | 264 | -0.32323548 | -1.6419375 | 0.039622642 |
| GO_PYRIMIDINE_NUCLEOSIDE_BIOSYNTHETIC_PROCESS | 27 | -0.5620675 | -1.6398096 | 0.02534113 |
| GO_PYRIMIDINE_RIBONUCLEOTIDE_METABOLIC_PROCESS | 22 | -0.5309124 | -1.638853 | 0.017612524 |
| GO_TETRAPYRROLE_METABOLIC_PROCESS | 51 | -0.43935 | -1.6351553 | 0.03359684 |
| GO_RESPONSE_TO_ARSENIC_CONTAINING_SUBSTANCE | 29 | -0.49626133 | -1.630911 | 0.03187251 |
| GO_PIGMENT_BIOSYNTHETIC_PROCESS | 43 | -0.4555317 | -1.6306273 | 0.020952381 |
| GO_TRANSCRIPTION_FROM_RNA_POLYMERASE_III_PROMOTER | 37 | -0.48541486 | -1.6293437 | 0.037698414 |
| GO_ANAPHASE_PROMOTING_COMPLEX_DEPENDENT_CATABOLIC_PROCESS | 75 | -0.5862918 | -1.6193306 | 0.038610037 |
| GO_ATP_GENERATION_FROM_ADP | 34 | -0.485384 | -1.6141101 | 0.03777336 |
| GO_GLYCERALDEHYDE_3_PHOSPHATE_METABOLIC_PROCESS | 16 | -0.62301135 | -1.6102706 | 0.035019454 |
| GO_NUCLEOTIDE_SUGAR_BIOSYNTHETIC_PROCESS | 19 | -0.56021273 | -1.6102378 | 0.043052837 |
| GO_PROTEIN_HYDROXYLATION | 15 | -0.5677345 | -1.6083629 | 0.033398822 |
| GO_BLASTOCYST_DEVELOPMENT | 58 | -0.4263645 | -1.6077529 | 0.036679536 |
| GO_REGULATION_OF_CELL_CYCLE_ARREST | 98 | -0.40143597 | -1.6013043 | 0.044401545 |
| GO_NCRNA_3_END_PROCESSING | 20 | -0.6295827 | -1.6004649 | 0.027613413 |
| GO_REGULATION_OF_ENDOPLASMIC_RETICULUM_STRESS_INDUCED_INTRINSIC_APOPTOTIC_SIGNALING_PATHWAY | 27 | -0.4470378 | -1.5984024 | 0.034274194 |
| GO_POSITIVE_REGULATION_OF_MITOCHONDRION_ORGANIZATION | 147 | -0.31263977 | -1.5967361 | 0.013409962 |
| GO_MITOCHONDRION_MORPHOGENESIS | 18 | -0.48473105 | -1.5950645 | 0.041257367 |
| GO_MATURATION_OF_SSU_RRNA | 39 | -0.57454693 | -1.5919036 | 0.049586777 |
| GO_PROTEIN_IMPORT | 149 | -0.3013056 | -1.589071 | 0.021782178 |
| GO_CELLULAR_PROTEIN_COMPLEX_ASSEMBLY | 309 | -0.3008453 | -1.5889106 | 0.012048192 |
| GO_REGULATION_OF_MITOCHONDRION_ORGANIZATION | 193 | -0.2995658 | -1.5885999 | 0.015655577 |
| GO_PTERIDINE_CONTAINING_COMPOUND_BIOSYNTHETIC_PROCESS | 15 | -0.679846 | -1.5884225 | 0.03875969 |
| GO_ALDITOL_METABOLIC_PROCESS | 17 | -0.6246969 | -1.586439 | 0.027777778 |
| GO_ENDOPLASMIC_RETICULUM_ORGANIZATION | 32 | -0.40604973 | -1.5821205 | 0.048681542 |
| GO_COFACTOR_METABOLIC_PROCESS | 305 | -0.37553358 | -1.5806143 | 0.023715414 |
| GO_NUCLEOSIDE_SALVAGE | 16 | -0.61183625 | -1.5782264 | 0.042307694 |
| GO_NUCLEOSIDE_PHOSPHATE_BIOSYNTHETIC_PROCESS | 143 | -0.3604391 | -1.576695 | 0.007858546 |
| GO_CELLULAR_METABOLIC_COMPOUND_SALVAGE | 35 | -0.48491094 | -1.5740808 | 0.043560605 |
| GO_HYDROGEN_ION_TRANSMEMBRANE_TRANSPORT | 77 | -0.3862648 | -1.5730457 | 0.04901961 |
| GO_REGULATION_OF_TELOMERASE_RNA_LOCALIZATION_TO_CAJAL_BODY | 15 | -0.66727895 | -1.5676436 | 0.036437247 |
| GO_CELLULAR_MODIFIED_AMINO_ACID_BIOSYNTHETIC_PROCESS | 49 | -0.46585664 | -1.5665842 | 0.03929273 |
| GO_METALLO_SULFUR_CLUSTER_ASSEMBLY | 15 | -0.57880044 | -1.5580999 | 0.049242426 |
| GO_PSEUDOURIDINE_SYNTHESIS | 17 | -0.671103 | -1.5502665 | 0.0407767 |
| GO_CARBOHYDRATE_CATABOLIC_PROCESS | 101 | -0.3656259 | -1.5442616 | 0.016728625 |
| GO_PROTEIN_IMPORT_INTO_NUCLEUS_TRANSLOCATION | 28 | -0.44074193 | -1.5408123 | 0.04715128 |
| GO_REGULATION_OF_CELLULAR_PROTEIN_CATABOLIC_PROCESS | 260 | -0.28942275 | -1.5377556 | 0.04696673 |
| GO_RIBONUCLEOSIDE_DIPHOSPHATE_METABOLIC_PROCESS | 59 | -0.3972287 | -1.533108 | 0.042307694 |
| GO_GLUCOSE_METABOLIC_PROCESS | 107 | -0.34704506 | -1.5295683 | 0.046153847 |
| GO_O_GLYCAN_PROCESSING | 54 | -0.48120078 | -1.5268134 | 0.028169014 |
| GO_DNA_STRAND_ELONGATION | 29 | -0.7005203 | -1.5265545 | 0.046692606 |
| GO_COENZYME_METABOLIC_PROCESS | 241 | -0.3553836 | -1.5225337 | 0.033333335 |
| GO_PIGMENT_METABOLIC_PROCESS | 52 | -0.4105953 | -1.5120357 | 0.031809144 |
| GO_REGULATION_OF_PROTEIN_OLIGOMERIZATION | 28 | -0.46161595 | -1.4917506 | 0.034412954 |
| GO_NUCLEOSIDE_PHOSPHATE_CATABOLIC_PROCESS | 68 | -0.3897585 | -1.4818515 | 0.026515152 |
| GO_CARBOHYDRATE_BIOSYNTHETIC_PROCESS | 114 | -0.34213677 | -1.4767277 | 0.03952569 |
| GO_REGULATION_OF_PROTEIN_STABILITY | 206 | -0.25397512 | -1.4681896 | 0.036960986 |
