# Supplementary material for: Immune landscape and a promising immune prognostic model associated with TP53 in early‐stage lung adenocarcinoma
Source: Cancer Med. 2020 Dec 12;10(3):806–23. doi: 10.1002/cam4.3655 (PMC7897963; doi:10.1002/cam4.3655)
Supplement: Supplementary file 6 — Table S6 [file CAM4-10-806-s006.docx]

**Supplementary table 6**: The GO analysis results of risk score-associated immune DEGs in early-stage LUAD patients.

| **Gene function** | **Gene count** | **Genes** | **P-value** | **FDR** |
| --- | --- | --- | --- | --- |
| Humoral immune response | 11 | PRSS3/C7/CTSG/REG1A/  CPB2/SFTPD/KRT6A/  BPIFA2/PGC/FGB/ENTPD2 | 9.680987e-14 | 4.874377e-11 |
| Antimicrobial humoral  response | 8 | PRSS3/CTSG/REG1A/  SFTPD/KRT6A/BPIFA2/  PGC/FGB | 6.096761e-14 | 4.874377e-11 |
| Defense response to  bacterium | 6 | CTSG/SFTPD/KRT6A/  BPIFA2/PGC/FGB | 3.060381e-07 | 6.163607e-05 |
| Regulation of immune effector  process | 5 | MIF/C7/CPB2/AGER/  PGC | 4.214230e-05 | 2.028716e-03 |
| Antibacterial humoral  response | 4 | CTSG/SFTPD/PGC/FGB | 7.496536e-08 | 2.516337e-05 |
| Positive regulation of  cytokine secretion | 4 | MIF/LRRK2/AGER/PAEP | 6.494874e-06 | 5.945762e-04 |
| Regulation of inflammatory  response | 4 | C7/LRRK2/CPB2/AGER | 8.179368e-04 | 1.218108e-02 |
